# Election cycles and global religious intolerance

**DOI:** 10.1073/pnas.2213198120

**Published:** 2022-12-29

**Authors:** Gareth Nellis

**Affiliations:** ^a^Department of Political Science, University of California, San Diego, La Jolla, CA 92093

**Keywords:** political science, religion, intolerance, prejudice, elections

## Abstract

Mass elections are key mechanisms for collective decision-making. But they are also blamed for creating intergroup enmity, particularly while they are underway; politicians use polarizing campaign strategies, and losing sides feel resentful and marginalized after results are announced. I investigate the impact of election proximity–that is, closeness to elections in time–on social cleavages related to religion, a salient form of group identity worldwide. Integrating data from ∼1.2 million respondents across 25 cross-country survey series, I find no evidence that people interviewed shortly before or after national elections are more likely to express negative attitudes toward religious outgroups than those interviewed at other times. Subgroup analysis reveals little heterogeneity, including by levels of political competition. Generalized social trust, too, is unaffected by election calendars. Elections may not pose as great a risk to social cohesion as is commonly feared.

## Significance

There is widespread concern that elections polarize societies along group lines. Meanwhile, enmity between religious communities is rising globally—a phenomenon often attributed to the dynamics of electoral competition. I examine one implication of this broader hypothesis by testing for the presence of electoral cycles in religious intolerance. Pooling responses from 1,086 country-level “values and attitudes” surveys, I evaluate whether people interviewed shortly before or after national elections are more likely to express intolerant attitudes toward those belonging to different religions or sects than people interviewed outside of election seasons. I find no statistical evidence that this is the case, either overall or among theoretically relevant subpopulations. Individuals interviewed close to elections also do not voice greater distrust in other people generally.

Do elections strengthen or undermine social cohesion? On the one hand, political scientists have long argued that inclusive democratic institutions represent humanity’s best mechanism for collective decision-making. They enable disagreements between individuals and communities to be resolved peacefully, and in a manner broadly acknowledged as legitimate (1–4). They can also spur the creation of new social-group alliances, as politicians compete to assemble winning coalitions of voters (5).

Yet, others stress the potential for mass elections, and the popularity campaigns they necessitate, to become dangerous flashpoints—especially during the period when they are happening (6–8). “[E]lections often have the effect of highlighting societal fault-lines and hence laying bare very deep social divisions” (9). Commentators in post-war Iraq observed that the 2005 election “seems to be sharpening sectarian and ethnic differences” (10), while during the 2014 Indonesian presidential race, a leading contender was repeatedly branded a “kafir” (heathen) by opponents, who alleged his mother was Christian (11). Such accounts point the finger at political elites. Candidates and parties frequently mobilize voters by appealing to citizens’ ascriptive group traits, “playing the ethnic card” and stoking fears about ethnic “others,” rather than focusing on economics and governance issues (12, 13). After election results have been announced, groups that won may do little to placate groups that lost, breeding alienation and bitterness. Elections in diverse societies are said to be marked by “tribalism” (14). Holding them may be a “great experiment” (15).

This paper aims to rigorously test, for a wide spectrum of cases, whether the time periods immediately surrounding elections see greater religious intolerance than other time periods. In particular, I investigate whether election campaigns and their aftermath catalyze popular hostility toward religious outgroups, meaning those who subscribe to a religion different from one’s own. Religion, definable as “a unified system of beliefs and practices relative to sacred things” (16), is far from the only identity category with which individuals may affiliate (17, 18). But it is arguably the most universal. While religiosity is declining, only a small minority of the world’s population professes no religious faith.^*^

A 2012 Pew estimate suggests that 16% of the global population is unaffiliated with a formal religion, although even “many of the unaffiliated hold some religious or spiritual beliefs;” see http://www.pewrsr.ch/3H9tLJ5. Religious conflict structures political competition in both low- and high-income settings. Violence has been perpetrated in the name of God for millennia (19).

Case studies give ample reason to suspect that election seasons exacerbate interreligious group tensions. Poland’s first postcommunist polls saw a surge in antisemitism (20). Hindu–Muslim riots in India are more likely to erupt in the 6 mo that precede state elections (21). Successive rounds of voting in Bosnia and Herzegovina have come to resemble ethnoreligious “censuses” following the passage of the Dayton Agreements (22). In many western countries, antiimmigrant invective, sometimes laced with references to newcomers’ “alien” religious backgrounds, is trumpeted loudly as elections approach (23).

Empirically, there are challenges in ascertaining whether a systematic relationship exists between elections and religious intolerance. Few countries keep a running tally of the attitudes of their citizens toward various religious communities, and isolating a causal connection is a tall order. In seeking to overcome these hurdles, this paper asks whether levels of religious intolerance fluctuate within countries when elections are near in time. Importantly, I do not set out to estimate the effect of elections per se. Since most countries today hold elections, a key question is how, counterfactually, religious intolerance would look had elections never been instituted. Unfortunately, this question is unlikely to be answerable: comparing average intolerance rates across countries that do and do not host elections runs clear risks of confounding and reverse causality, and, so far, no credible natural experiment has been proposed. I later discuss how learning about election cycles in religious intolerance might nevertheless shed some light on the matter of elections’ overall impacts.

I identify and harmonize 25 multinational “attitudes and values” survey series that measure intolerance toward religious outgroups (*SI Appendix*, Tables S8). I collect responses to all questions in which sampled individuals were asked to cast a personal judgment about a member of a religious or sectarian group different from their own. Questions cover trust in members of religious outgroups, favorability toward them, levels of comfort or discomfort in their presence, and willingness to have them as neighbors, friends, work acquaintances, citizens, or family members. To standardize, I recode all answers to take the value of one if the respondent offered an intolerant attitude toward the religious group they were asked about and zero if they offered a neutral or tolerant attitude (*SI Appendix*, Table S12). This yields approximately 2.6 million date-stamped, consistently coded question-items, which come from 1,225,173 unique interviews conducted in 150 countries—1,086 country-level surveys in total—between the years 1982 and 2022. To my knowledge, this constitutes the largest integrated body of microdata on religious intolerance compiled to date.

Statistically, I test for the presence of electoral cycles in religious intolerance, exploiting temporal variation within countries. The core idea is straightforward: i) socially divisive campaigning concentrates in the months just before an election takes place, as politicians try to sway voters’ minds; and ii) feelings of resentment and exclusion among losing-side supporters—as well as any residual bad blood from campaigns—will be most intense in the months just after the declaration of a winner. If elections engender group-wise animus, those interviewed shortly before or after an election can thus be expected to express more negative sentiments toward religious outgroups than those interviewed further from an election in time.

For the analysis, I run a set of weighted least-squares regressions of the dichotomous measure of religious intolerance on a treatment indicator denoting whether a respondent was interviewed 3, 6, or 9 mo before and/or after a national election. [The National Elections Across Democracy and Autocracy dataset (24) used to construct these indicators provides a comprehensive list of executive, legislative, and constituent assembly national elections and their dates up to the end of the year 2020 (*SI Appendix*, Table S11)]. Observations are respondent/question-items. The benchmark model controls for country, question-type, and question-target fixed effects,^†^ weights country/election-cycles equally, and clusters standard errors by country/election-cycle. Under the assumption that election timing is conditionally independent of individuals’ potential outcomes, the results have a causal interpretation. Reassuringly, I find that election proximity is unpredictive of survey respondents’ “pretreatment” characteristics (*SI Appendix*, Table S3) as well as rates of nonresponse to questions about religious tolerance (*SI Appendix*, Fig. S6). While estimates are valid only for the sample at hand, the data are encompassing. There is no within-country evidence that elections marred by violence and civilian deaths are either more or less likely to have had surveys fielded close to them in time than peaceful elections (*SI Appendix*, Table S10). I later show that the results are unchanged after omitting elections whose timing may have been strategic (Fig. 3).

It is important to clarify that stated attitudes about religious outgroups enfold two components: an individual’s internal attitude about that group and her perception of prevailing norms about the acceptability of articulating that attitude to other people (e.g., to a survey enumerator). While the present design cannot disentangle these parts, both are of interest, and both may be shifted by the election context. Perceptions of social norms have been shown to influence behaviors (25, 26). Historians have singled out the mounting dehumanization of Jews in European public discourse in the 1920s as a contributing factor to the Holocaust (27). One perpetrator of the Rwandan genocide later told an ethnographer that hate speech on the radio “encouraged people to participate because it said ‘the enemy is the Tutsi”’ (28). Intuitively, the more permissible it is to vocalize dislike of an outgroup, the more the security and well-being of that outgroup are jeopardized.

## Results

**Fig. 1.**
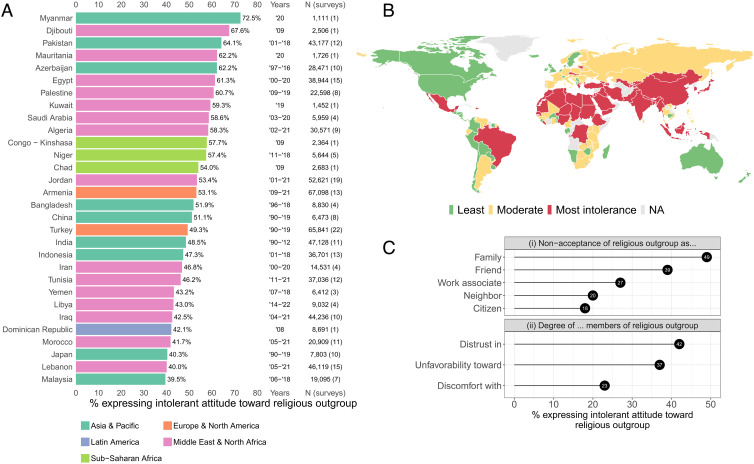
Average unweighted survey responses to questions about intolerance toward religious outgroups across 25 multinational survey series. Note, surveys pose different questions about religious intolerance across countries, as well as within countries over time; further, the number and timing of surveys varies cross-nationally. Panel *A* plots intolerance rates for the 30 countries with the largest share of intolerant responses; the Years column provides the range of years covered by the integrated surveys for a given country. Panel *B* maps average intolerance, split into terciles, by country. Panel *C* shows average intolerance rates by question-type in the full sample.

Fig. 1 graphically summarizes my measure of religious intolerance. Fig. 1*A* shows the 30 countries in which the share of negative attitudes offered about religious outgroups is largest in the sample. Myanmar, whose military orchestrated an ethnic cleansing against the country’s Muslim Rohingya population in 2017, sits at the top, based on a survey taken in 2020.^‡^ Fig. 1*B* maps the country averages of the measure, split by tercile. Sub-Saharan Africa, the MENA region, and the Asia-Pacific display the highest levels of intolerance (although a caveat is that the number of surveys taken, their time span, and the specific questions asked vary across countries). Fig. 1*C* plots the overall percentages of intolerant attitudes offered in response to each of the question-types represented in the sample. People prove most averse to having members of religious outgroups as family members: 49% are unwilling to accept them in that role (e.g., as sons- or daughters-in-law). Worldwide, 42% of respondents say they are distrustful of people belonging to religions or sects not their own.

**Fig. 2.**
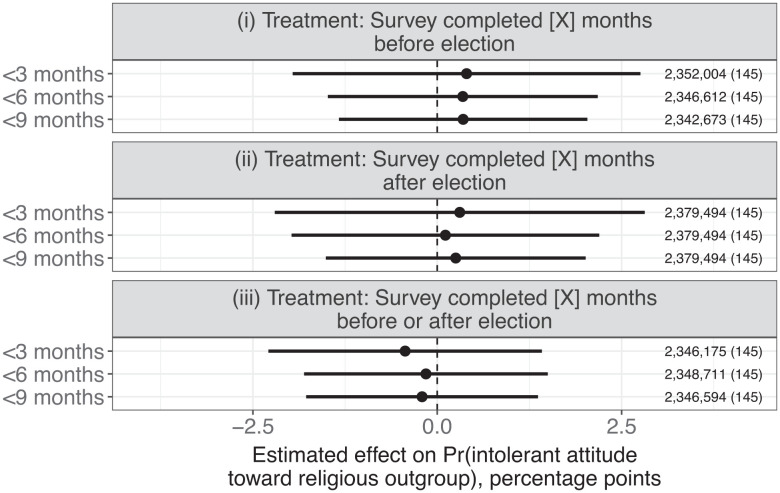
Coefficient plots of estimates from nine weighted least-squares regressions. Each regression employs a differently specified treatment variable, described in the panel titles and on the vertical axis. The unit of analysis is the respondent/question-item. Models include country, question-type, and question-target fixed effects. Only responses for which the survey date can be accurately pinpointed to within a 6-mo time bracket are included. Observations are weighted such that each country/election cycle contributes equally in estimation. 95% confidence intervals are based on robust standard errors clustered by country/election cycle. The number of observations, with the number of countries in parentheses, is displayed on the right-hand side of the plot.

Do individuals express more intolerant attitudes toward religious outgroups when elections are close in time? Across a raft of specifications, I find no evidence to support this claim. Fig. 2 visualizes the coefficients and 95% confidence intervals for analyses using the benchmark specification and the nine versions of the treatment variable. Point estimates are inconsistently signed and do not surpass 0.4% points in absolute magnitude. Given the large number of observations and clusters, the estimated confidence intervals are narrow, allowing me to rule out effects larger than 1.4 to 2.8% points, depending on the model (*SI Appendix*, Table S5).^§^ For all tests, I fail to reject the null hypothesis of no effect at the 95% level.

I assess the sensitivity of the results to key analytical decisions. In a specification curve analysis, I show that the study’s conclusions do not hinge on the choice of weighting scheme, fixed effects, standard-error clustering level, or on the exclusion of less precisely dated observations (*SI Appendix*, Fig. S1). I estimate the effects for the four largest global survey series separately; strikingly, there are statistically significant effects in opposite directions for two of the biggest series (*SI Appendix*, Fig. S2), underlining the value of meta-studies like this one. Results do not appreciably vary across global regions (*SI Appendix*, Fig. S3) nor across the religious groups detailed in the question-text (*SI Appendix*, Fig. S4) Conclusions are unchanged when entering pre- and post-election period indicators jointly into the regression (*SI Appendix*, Table S4). There is no perceptible relationship between the outcome and a continuous coding of the treatment variable (*SI Appendix*, Fig. S9).

**Fig. 3.**
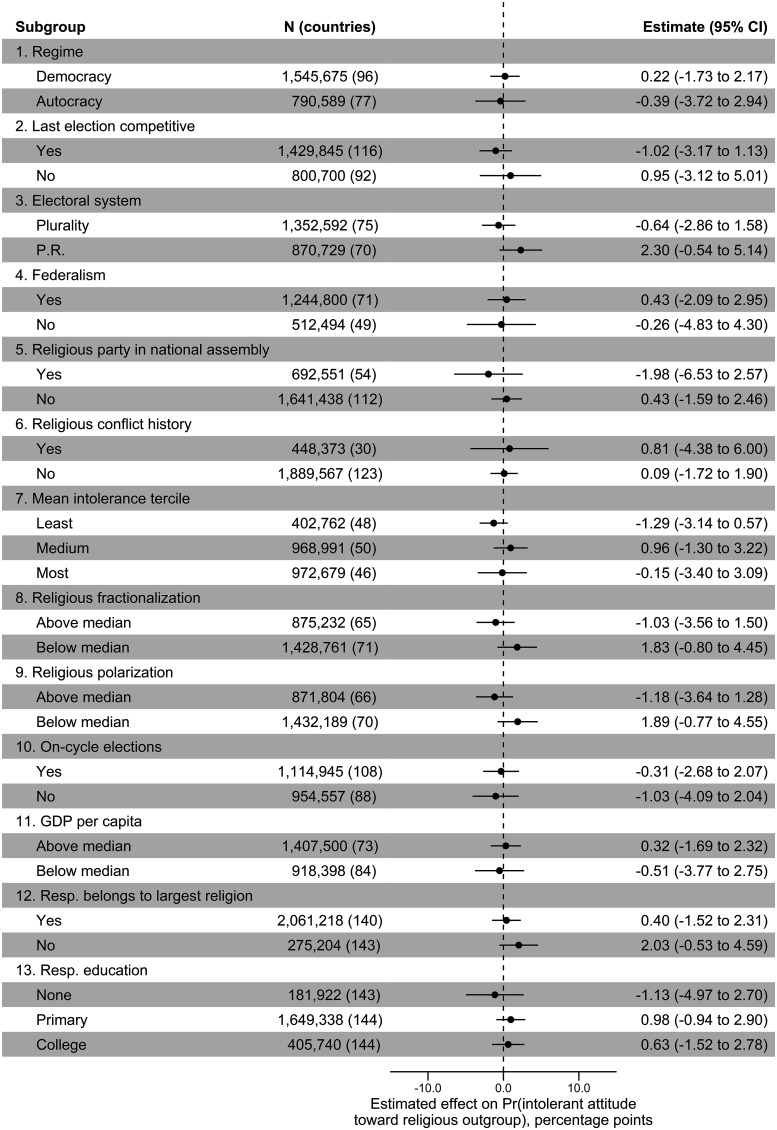
Subgroup effects. Estimates from weighted least-squares regressions that include country, question-type, and question-target fixed effects. The treatment variable is an indicator for whether the respondent was surveyed within 6 mo prior to a national election. The unit of analysis is the respondent/question-item. Only responses for which the survey date can be accurately pinpointed to within a 6-mo time bracket are included. Observations are weighted such that each country/election cycle contributes equally in estimation. 95% confidence intervals are based on robust standard errors clustered by country/election cycle.

The results show that attitudes toward religious outgroups do not track elections. But the aggregate findings may mask subgroup variability. Fig. 3 considers 13 dimensions of potential heterogeneity, focusing on the treatment indicator for whether a respondent was interviewed 6 mo prior to an election. (The construction of the moderating variables is described in *SI Appendix*, Table S2). I now summarize the findings in accordance with the numbered headings provided in Fig. 3:

1. We might expect election cycles in religious intolerance to be manifest in democracies, where opposition parties have the opportunity to contest freely, and not in electoral autocracies, where campaigns tend to be tightly choreographed by authoritarian incumbents. However, the estimated effect of election proximity is null for democracies and autocracies.
2. By a similar logic, election proximity effects may be present in electorally competitive environments, where campaign stakes are high and elite incentives to lean on polarization strategies are strongest, but not in uncompetitive ones. Yet, the results turn out to be null for both subgroups.
3. Proportional representation has been portrayed as an election rule that fosters consensus-building, whereas plurality voting systems are said to encourage majoritarianism (29). Election cycles in religious intolerance are not observable under either system.
4. Political federalism might promote the emergence of regional identities and so lessen the significance of “macro cleavages” like religion (30). Alternatively, elites holding positions of power at lower elected tiers might come to the aid of copartisans in national races by engaging in divisive campaigning. Still, the results are not moderated by this institutional feature.
5. The existence of major religious parties within the national political system might bolster the likelihood that religion becomes politicized in the lead-up to elections; it could also proxy for the greater salience of religion in politics generally. No such pattern is evident in the data, however.
6. Countries with a previous history of violent religious conflict—involving 25 or more battle deaths—do not see electoral cycles in religious intolerance nor do countries without such a history.
7. Results are similar when grouping countries by the terciles of intolerance depicted in Fig. 1*B*.
8. Election proximity effects are not apparent in countries with either higher or lower degrees of religious fractionalization (the probability that two randomly selected people from a country do not belong to the same religion) suggesting that the demographic balance between religious groups is not a conditioning factor.
9. The same is true when partitioning the sample according to a measure of religious polarization, for which higher values indicate that the distribution of religious groups is more bipolar.
10. The coefficient on the treatment indicator remains statistically insignificant when restricting the sample to individuals interviewed between two elections that were both held on time, according to a preset calendar (usually constitutionally mandated). This analysis addresses the possibility that election timing could be endogenous to the occurrence of waves of religious intolerance in certain cases—e.g., if elections are postponed because of social conflict or, inversely, called early because far-right incumbents think they might profit from religious unrest.
11. Being surveyed 6 mo before an election does not measurably increase expressions of religious intolerance in either higher- or lower-income countries.
12. There are no statistically significant effects of being interviewed just before an election, regardless of whether the respondent is affiliated with their country’s plurality religion or not.
13. There are no discernible effects of electoral proximity for respondents with more or less education.

Heterogeneous treatment effects for the other treatment indicators are displayed in *SI Appendix*, Fig. S11, while *SI Appendix*, Table S7 presents estimates of subgroup effects for democratic countries only. The null results persist in virtually all models.

One explanation for the slate of overwhelmingly insignificant effects is that religious divisions are not sufficiently entrenched in enough countries for elections to make a difference. On this theory, cyclical effects would be apparent in an investigation centered on other, more locally relevant ethnic cleavages (e.g., race in Brazil, language in Belgium, and cousinage relations in Mali).

I underscore that this possibility does not undermine my central finding of elections’ noneffect on religious intolerance, a domain of foremost policy interest, notably within the United Nations and the US government.^¶^ But to address it holistically, I generate an additional outcome—generalized social distrust—retrieved from the same set of 25 multinational surveys described above. Most survey instruments include a question of the form: “Generally speaking, would you say that most people can be trusted or that you need to be very careful in dealing with people?” I code a binary variable that takes one when the respondent offered a negative answer (i.e., stated generalized distrust) and zero otherwise. If elections erode intergroup relations—whatever the politically relevant cleavages might be on the ground—it stands to reason that those interviewed closer to an election-day will express diminished trust in “most” other people.

**Fig. 4.**
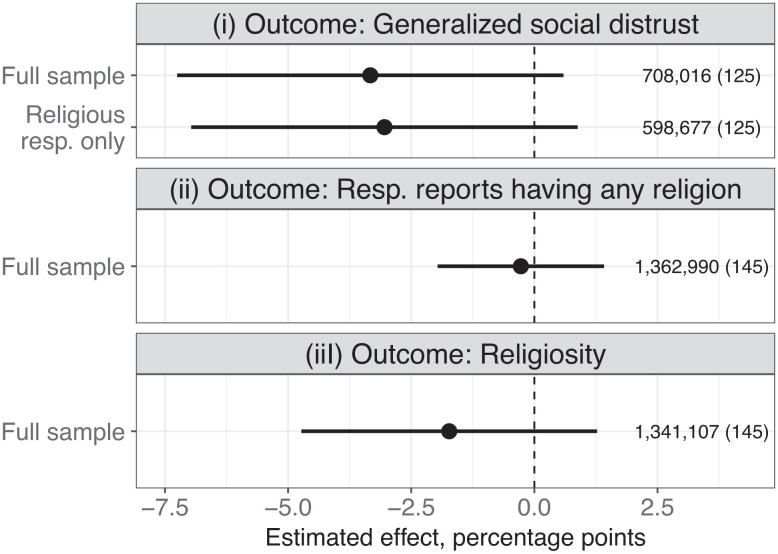
Coefficient plots of four weighted least-squares regressions that include country fixed effects. The treatment variable is an indicator of whether the respondent was surveyed within 6 mo prior to a national election. The unit of analysis is the respondent. Only responses for which the survey date can be accurately pinpointed to within a 6-mo time bracket are included. Observations are weighted such that each country/election cycle contributes equally in estimation. 95% confidence intervals are based on robust standard errors clustered by country/election cycle. The number of observations, with the number of countries in parentheses, is displayed on the right-hand side of the plot.

Fig. 4*i* runs the same specification as that employed for Fig. 2, now plugging in this alternative outcome. Results are displayed both for analyses incorporating responses from all interviewees in the compiled surveys and for the subset of interviewees who declared a religious faith (to facilitate comparison with the earlier findings). Ultimately, Fig. 4*i* uncovers no statistically significant effect of being surveyed close to an election on generalized social distrust. *SI Appendix*, Fig. S8 further demonstrates either no effects or scattered negative effects for subgroups based on higher (versus lower) levels of ethnic, linguistic, and religious fractionalization, answering the concern that generalized social distrust may pick up attitudes toward in-groups in places with clear ethnic majorities. Taken together, the evidence on this extra outcome broadens the case for elections’ minimal social effects.

The middle and bottom panels of Fig. 4 explore, respectively, whether people are more likely to report having any religion or greater religiosity in the time-window surrounding elections. Prior literature finds that electoral proximity strengthens in-group attachments in some settings (31–33). But using both a binary variable for whether a respondent declared having a religion during the survey, as well as a consolidated measure of personal religiosity (*SI Appendix*, Table S2), I find no backing for that hypothesis here.

## Discussion

Tajfel famously concluded that “[o]utgroup discrimination is extraordinarily easy to trigger” (34). As focal events for heated ethnic rhetoric and the propagation of group stereotypes, elections have been cited as a potent threat to intergroup harmony in diverse societies. Such concerns have propelled ambitious schemes to mitigate elections’ centrifugal impacts: power-sharing arrangements that guarantee all major social groups a seat at the governing table, and “integrationist” institutions that encourage elites to vie for support across group lines (35–37). Yet, these apprehensions and remedies presuppose that elections do in fact sow discord, a claim for which the evidence—beyond single-country studies—is surprisingly thin. By testing for the presence of election cycles in religious intolerance, I help bridge this evidentiary gap.

What are we to make of the clear if unexpected finding that election timing is unrelated to hostility toward people aligned with other religions? It could be that outgroup prejudice is “hardwired”: the product of childhood socialization and thus not amenable to short-term flux. However, this account is hard to square with randomized controlled trials showing that individual prejudice against outgroups responds quickly, inter alia, to brief in-person conversations (38), radio shows (39), participation in mixed sports teams (40, 41), and perspective-taking exercises (42). Other interventions further attest to the malleability of prejudice, demonstrating only fleeting effects that last days or even hours (43). Real-world elections far exceed these microinterventions in visibility and reach and yet produce no analogous impacts.

Another explanation is that, in an era of mass communications, electioneering—and exposure to it—is not confined to the immediate election period but is a near-permanent fixture of daily life in highly connected societies. Accordingly, the absence of cyclical patterns in attitudes toward religious outgroups may reflect a ceiling effect. “Ethnic entrepreneurs” are perennially fanning the flames of communal animosity with the next election in mind, and grievances from past group disputes do not dim. Note, this line of argument would also be consistent with the view that prolonged experience of elections may aggravate religious intolerance—compared to a world with no elections—even though within-country election cycles in intolerance do not occur. There are reasons to be skeptical, however. Cutting against the claim, the mean rate at which individuals offer a religiously intolerant attitude is 31% in the sample, indicating plentiful room for growth. Next, even in the most politicized countries, there is a step-change in the volume of campaigning that goes on just before the polling day; rates of partisan self-identification rise around that time too (32). If elections were religiously polarizing events and individual prejudice is at least partly susceptible to change over time, it would be odd to see no movement in religious intolerance at these moments, but this is what I find.

The significance of political party bases also merits consideration. For societies characterized by cross-cutting cleavages, Posner (44) theorizes that which axis of identity (e.g., language or tribe) is salient at a given time is a function of the current party system. In other words, parties and their candidates play a critical role in activating certain social-identity categories and not others. If religion rarely forms the basis for partisan cleavages in actuality, then that may be what drives the null result seen here. But the available evidence does not give much credence to this notion. Expressly religious parties with constituencies constructed around religious appeals hold more than 5% of national assembly seats in 27% of country/election years in my analysis dataset (*SI Appendix*, Table S1). *SI Appendix*, Fig. S13 plots histograms from a granular investigation of parties in 88 countries, from which I estimate that the mean national vote share secured by parties dependent on various forms of religious mobilization lay between 25% and 30% when the expert survey was taken in 2008-09. Religion, then, structures partisan coalitions in numerous countries. In conjunction with the lack of any consistent pattern of subgroup effects according to the variables just noted (see Fig. 4 and *SI Appendix*, Fig. S12), we are left with the conclusion that election cycles in religious intolerance do not systematically materialize even in circumstances where religious and partisan cleavages align.

A simpler rationalization is that most elections in most countries are not searingly contentious events. For better or worse, many citizens take little interest in formal politics. Those who cast ballots routinely do so for moderate parties—not always because of group animosity but because of “bread and butter” issues. While religious parties flourish in many countries, other states enforce laws that ban ethnoreligious parties and identity-based campaign messaging altogether (45). Authoritarian regimes may be especially eager to ensure that rigged elections are not socially destabilizing. Religious leaders can step in to calm communal tensions, not only inflame them (46). Elections held in the shadow of ethnic conflict may be greeted by war-weary populations as far preferable to violence. In democracies, elections may embody proud national traditions about how a country is to be governed and thus serve as moments when inclusive civic nationalism comes to the fore. By zeroing in on fraught races, global news coverage may present a distorted picture of how the lion’s share of elections operates, perhaps shaping common beliefs about their pitfalls.

To be sure, the null estimated effects should not be taken to imply that elections never have adverse social ramifications. Studies have shown that victory by extremist candidates can embolden attitudes against minorities (47, 48). Narratives from particular settings also make plain that election processes are capable of driving a wedge through society. But the findings adduced here indicate that such negative side effects may be atypical, at minimum with regard to overtime changes in religious group relations.^#^ This is a meaningful lesson for policymakers skeptical about the wisdom of mass voting.

## Materials and Methods

I describe the empirical strategy for estimating the relationship between temporal proximity to elections and expressions of religious intolerance.

### Survey Coverage.

I combine data from (to the best of my knowledge) all multinational “attitudes and values” survey series that a) have asked questions about individuals’ attitudes toward members of a religious community that is not their own; b) make respondent-level microdata freely and publicly available online; and c) gathered survey data after the year 1980. Included survey series, along with brief descriptions, are listed in *SI Appendix*, Table S8. To find such surveys, I relied on the “Historical Overview of International Data Resources” maintained by the Gesellschaft Sozialwissenschaftlicher Infrastruktureinrichtungen (available at: bit.ly/3QYO3JM, filtering the list to all surveys that fall under the category, “Attitudes, Values, Beliefs”) and the extensive survey list maintained by the Association of Religion Data Archives (available at: bit.ly/3yt1Q3T). Surveys that were considered but not included are listed in *SI Appendix*, Table S9, which also gives the reasons for surveys’ exclusion.

Most included surveys were fielded among nationally representative samples of adults; others were administered to representative samples of well-defined demographic subpopulations (e.g., citizens belonging to the country’s majority religion, or young people). I opted not to include surveys or survey series carried out in a single country owing to concerns about data consistency and quality. I excluded respondents below the age of 15. Eighty-nine percent of subjects in the integrated dataset were interviewed in-person; the rest were interviewed by phone, mail, or through the internet.

### Outcome Measure.

I found that attitudes and values surveys have quizzed respondents about personal tolerance toward religious outgroups in four ways: i) by asking about respondents’ willingness to accept a member of a religious outgroup occupying a social role near to or far from them—as a family member, friend, neighbor, work-acquaintance, or citizen (e.g., “Would you be willing to have a neighbor of a different religion?”); or by asking about respondents’ ii) trust in, iii) favorability toward, and iv) level of comfort with members of other religious groups. The analysis dataset pools together all four question types.

Whereas some question-wordings inquire about respondents’ attitudes toward “people of a different religion,” others specify a “target religion” (e.g., “How pleasant do you find these contacts with Muslims?”). In instances where a target religion is stated, I only code outcomes for individuals who personally identify with a religion that is different from the target religion mentioned, ensuring that observations always capture judgments about religious outgroups (from the respondent’s perspective). I omit from the main estimation sample individuals who state that they have no religion—including atheists and agnostics—since it does not make sense for them to have attitudes toward someone of a different faith.

A small minority of questions measure attitudes toward religious sects/denominations (e.g., Roman Catholics and Sunnis) rather than whole religions. Since those questions still pertain to views about religious outgroups, I include them in the dataset.

Many surveys queried respondents about their attitudes toward multiple religious outgroups (e.g., asking about an individual’s trust in Christians, Buddhists, and Muslims separately). Each such response is assigned its own row in the analysis dataset; thus the unit of analysis in the main statistical model is the respondent/question-item. Analysis weights and standard-error clustering account for this feature of the dataset construction.

### Defining Treatment.

The main analyses use binary treatments built from two data sources. First, election dates come from the National Elections Across Democracy and Autocracy (NELDA) dataset, which provides a complete list of all national-level executive, legislative, and constituent assembly elections held between 1945 and 2020. Both democratic elections as well as elections held under autocratic or hybrid regimes appear on the list. I consider all national elections in the main analysis. Subgroup tests break down estimated effects by regime type.

Second, survey dates come from the surveys themselves. There is variability in the precision with which individual survey dates are recorded, both across and within survey series. For 58% of respondents in the sample, the original datasets give either the exact day or month on which a survey was administered. For the remaining cases, the survey organizations’ technical documentation only provides the range of dates between which surveys were fielded in each country (e.g. Wave 4 of the World Values Survey was run in Uganda between March 3 and March 18, 2001). Where I lack the exact individual dates, therefore, I merge this information into the dataset and impute the individual survey date using the midpoint of the given date-range. Note, the median size of the date ranges for this segment of the sample is just 30 d, meaning that any imprecision is quite minimal. Nonetheless, I probe the sensitivity of the results to dropping observations associated with wider date ranges (*SI Appendix*, Fig. S1).

Finally, I calculate the number of days between the date on which an individual was surveyed and a) the date of the next upcoming national election, and b) the date of the most recent (prior) national election. Using these quantities, I define nine treatment variables: indicators for whether an individual was surveyed 3, 6, or 9 mo i) before an election, ii) after an election, and iii) before or after an election. I also code a continuous measure describing what percentage way through the current election cycle the interview took place (see *SI Appendix*, Table S2 for further details on how this was computed).

### Statistical Model.

For the benchmark statistical model, I fit linear regressions of the following form:

[1] $$\begin{array}{c} Y_{q,a,b,c,t}=\beta T_{q,c,t}+\gamma_{c}+\theta_{a}+\zeta_{b}+\epsilon_{c,e}. \end{array}$$

Here, *Y* is the outcome recorded for question-item *q*, of question-type *a*, pertaining to question-target *b*, measured in country *c* at time *t*; *γ*_*c*_ represents country fixed effects, *θ*_*a*_ represents question-type fixed effects, *ζ*_*b*_ represents question-target fixed effects, and *ϵ*_*c*, *e*_ is the idiosyncratic error term, where *e* indexes election cycle (operationally, the date of the most recent prior election in country *c*). *T* is the (variously specified) treatment indicator for whether the question-response was recorded within a given time-window surrounding an election, and *β* is the estimated effect of election proximity, under the identifying assumptions. Standard errors are clustered at the level of the country/election cycle. Importantly, the number of outcomes measured per country/election cycle varies by how many surveys happen to have been administered at a given time and place, and how many questions about religious outgroups happen to have been asked. I weight each observation by 1 divided by the number of complete question item-level outcomes from that country/election cycle, such that each country/election cycle contributes equally in estimation.

## Supplementary Material

Appendix 01 (PDF)

## Data Availability

Previously published data were used for this work (multiple; see *SI Appendix*, Tables S2 and S8 for lists and descriptions). Replication data and code are available on the Harvard Dataverse: https://doi.org/10.7910/DVN/S1T3AF.

## Footnotes

^*^A 2012 Pew estimate suggests that 16% of the global population is unaffiliated with a formal religion, although even “many of the unaffiliated hold some religious or spiritual beliefs;” see pewrsr.ch/3H9tLJ5.

^†^Question-type fixed effects are binary variables for whether the question asked about trust, favorability, discomfort, or willingness to have religious outgroups occupy various social roles; question-target fixed effects are binary variables for the religious outgroup specified in the question text (e.g., “Different religion,” “Buddhist,” “Sunni,” and so forth).

^‡^There are several countries where religious intolerance is high, even though religious minorities comprise a small share of the national population. Scapegoating small minorities may be electorally advantageous for majoritarian elites, insofar as it involves alienating fewer voters.

^§^*SI Appendix*, Fig. S10 plots the distribution of unadjusted country-level differences in means of intolerance rates according to election proximity. The distribution centers near-symmetrically around zero.

^¶^The issue’s prominence is echoed in the International Religious Freedom Act, signed into law by President Bill Clinton in 1998, and in Article 18 of the Universal Declaration of Human Rights.

^#^The estimated null effect dovetails with the finding that ethnic-group cooperation is far more the norm than ethnic-group conflict (49).
