# Supplementary material for: Election cycles and global religious intolerance
Source: Proc Natl Acad Sci U S A. 2022 Dec 29;120(1):e2213198120. doi: 10.1073/pnas.2213198120 (PMC9910473; doi:10.1073/pnas.2213198120)
Supplement: Supplementary file 1 — Appendix 01 (PDF) [file pnas.2213198120.sapp.pdf]

SUPPORTING INFORMATION

“Election Cycles and Global Religious Intolerance”

Gareth Nellis*

**Department of Political Science, University of California, San Diego*

December 2022

Table of Contents

A Additional figures **3**

B Additional tables **16**

A Additional figures

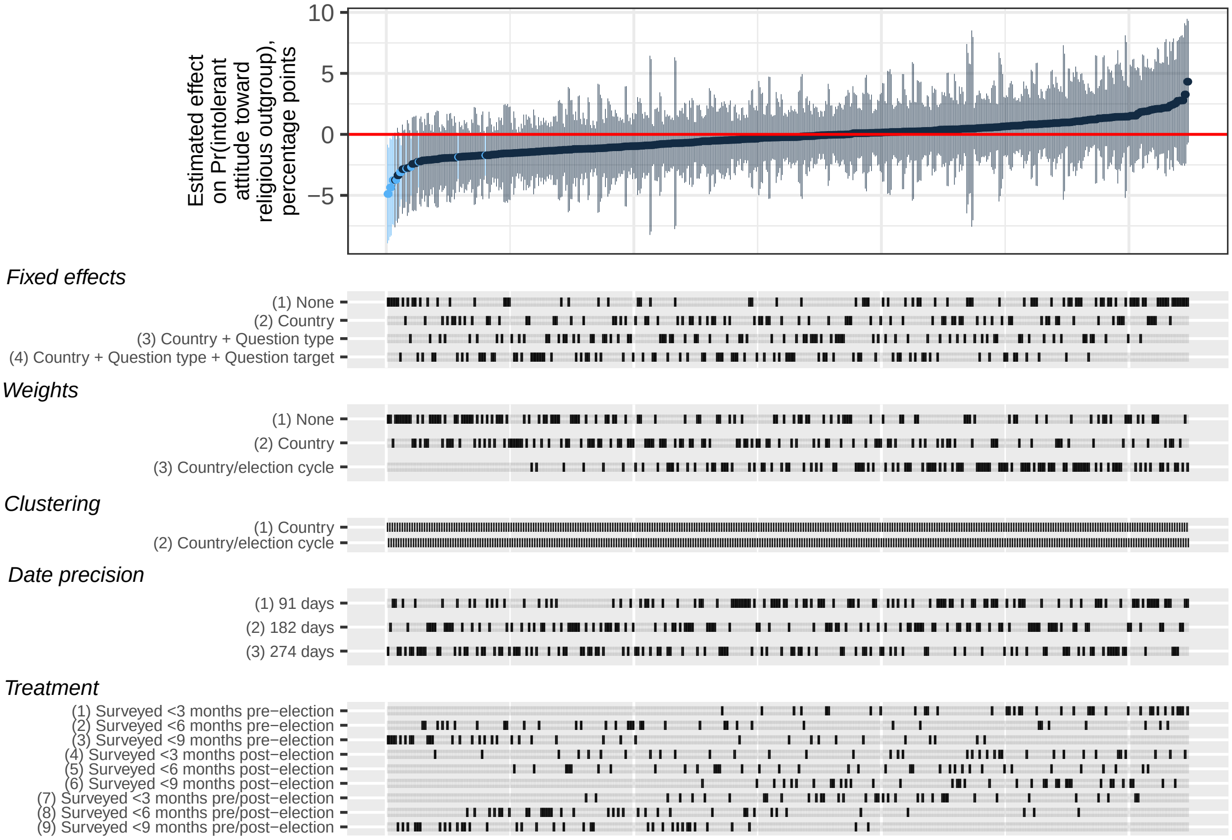

Figure S1: Specification curve analysis assessing the robustness of the primary results to all combinations of modeling choices. The upper coefficient plot shows point estimates, ordered by magnitude, and 95 percent confidence intervals. Light blue coefficients are those for which the null hypothesis of no effect is rejected at the 95 percent confidence level. (In total, 14 out of 648, or 2 percent of estimates are statistically significant at the 95 percent level, and all significant effects are associated with negatively signed coefficients.) The lower plots show the analytical choices made—with each affirmative choice denoted by a black tick—in the generation of each vertically aligned coefficient in the upper plot. *Date precision* refers to the size of the time bracket within which an individual's survey date can be accurately pinpointed. *Clustering* refers to the level at which standard errors are clustered.

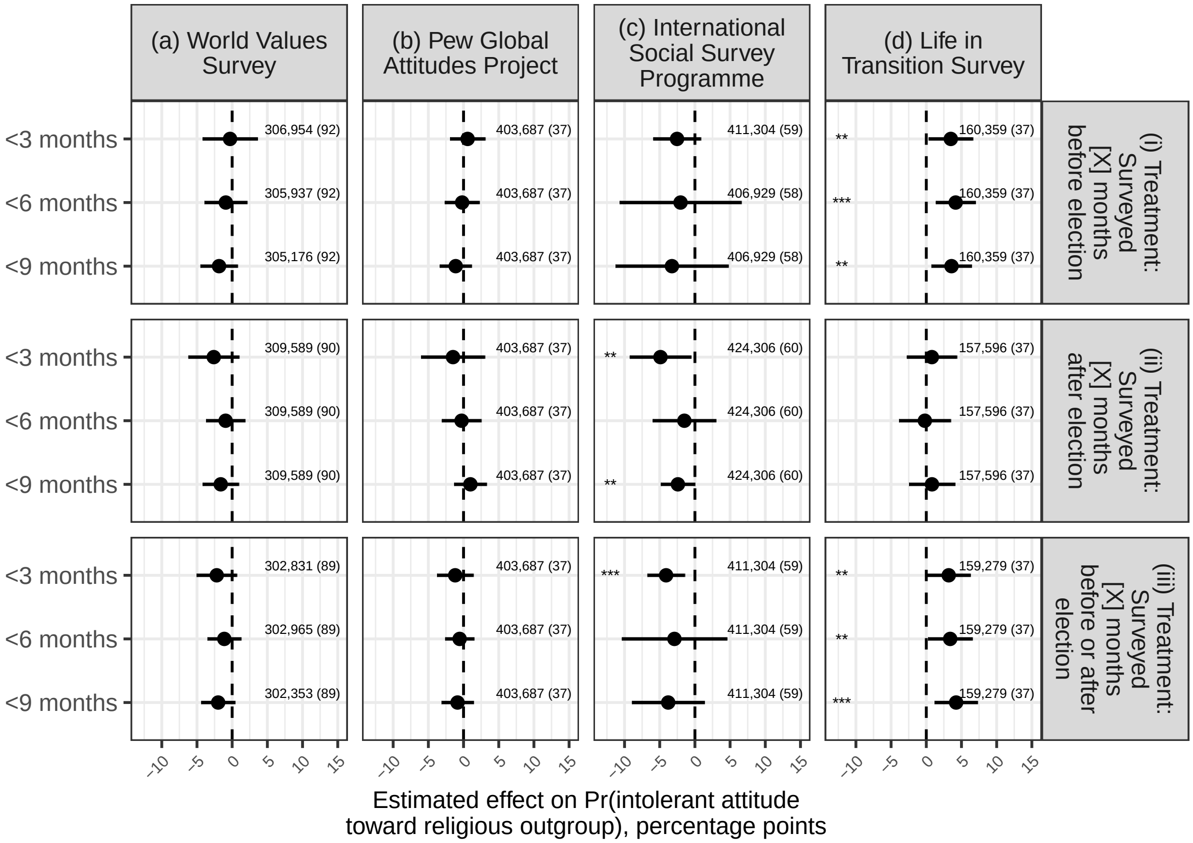

Figure S2: Coefficient plots of estimates from weighted least-squares regressions for the four cross-regional survey series that have the largest overall samples. Regressions employ variously specified treatment variables, described on the right and left vertical axes. The unit of analysis is the respondent/question-item. Models include country, question-type, and question-target fixed effects. Only responses for which the survey date can be accurately pintpointed to within a six-month time bracket are included. Observations are weighted such that each country/election cycle contributes equally in estimation. 95 percent confidence intervals are based on robust standard errors clustered by country/election cycle. The number of observations, with the number of countries in parentheses, is displayed on the right-hand side of the plots. Statistical significance is indicated on the left-hand sides of the plots: *p<0.1; **p<0.05; ***p<0.01.

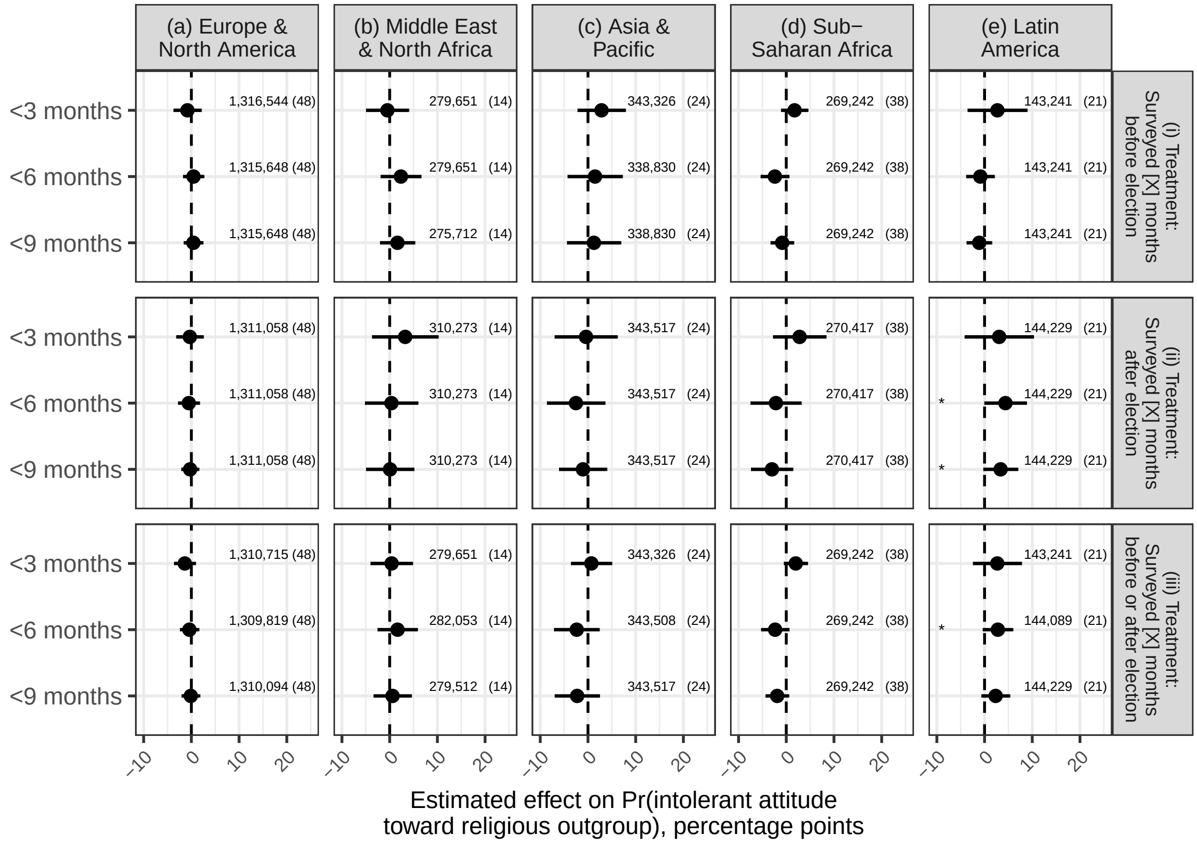

Figure S3: Coefficient plots of estimates from weighted least-squares regressions for each global region. Regressions employ variously specified treatment variables, described on the right and left vertical axes. The unit of analysis is the respondent/question-item. Models include country, question-type, and question-target fixed effects. Only responses for which the survey date can be accurately pintpointed to within a six-month time bracket are included. Observations are weighted such that each country/election cycle contributes equally in estimation. 95 percent confidence intervals are based on robust standard errors clustered by country/election cycle. The number of observations, with the number of countries in parentheses, is displayed on the right-hand side of the plots. Statistical significance is indicated on the left-hand sides of the plots: *p<0.1; **p<0.05; ***p<0.01.

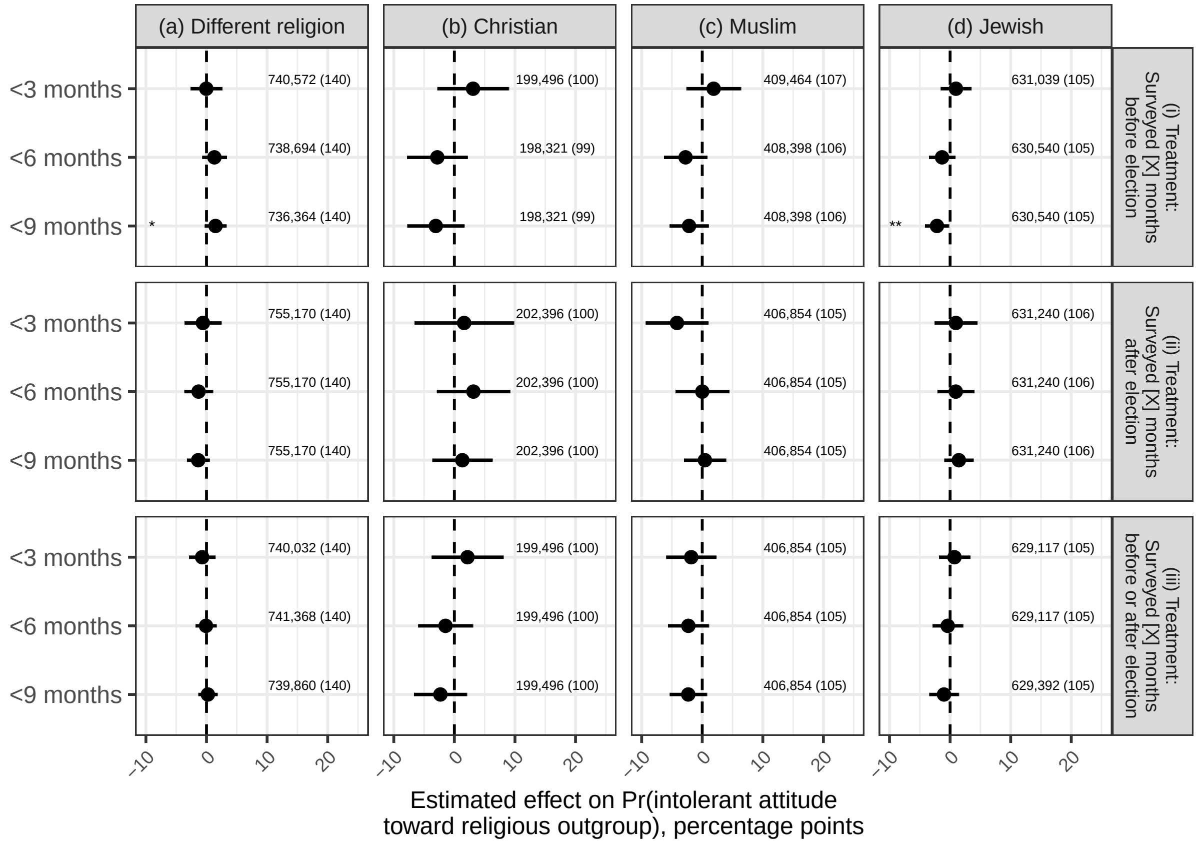

Figure S4: Coefficient plots of estimates from weighted least-squares regressions for the four most numerous "target" religious groups (including "Different religion") specified in the question-text. Regressions employ variously specified treatment variables, described on the right and left vertical axes. The unit of analysis is the respondent/question-item. Models include country and question-type fixed effects. Only responses for which the survey date can be accurately pintpointed to within a six-month time bracket are included. Observations are weighted such that each country/election cycle contributes equally in estimation. 95 percent confidence intervals are based on robust standard errors clustered by country/election cycle. The number of observations, with the number of countries in parentheses, is displayed on the right-hand side of the plots. Statistical significance is indicated on the left-hand sides of the plots: *p<0.1; **p<0.05; ***p<0.01.

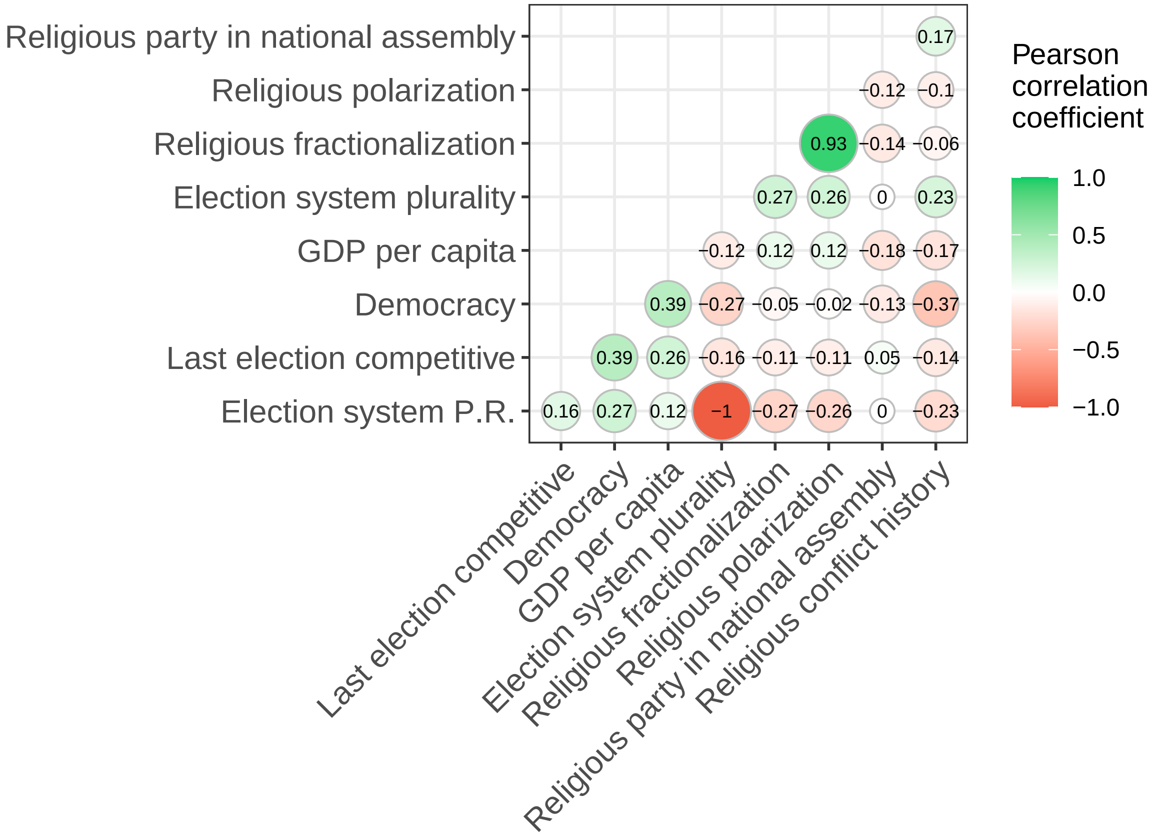

Figure S5: Correlation plot visualizing the bivariate correlation coefficients between all pairs of nine country- or country/election-level variables used in the analysis of subgroup effects. The unit of analysis is the country/election year. There are 525 observations. For comparability, only country/election years that contain non-missing data for all nine variables are analyzed. *Democracy*, *Election system plurality*, *Election system P.R.*, *Religious conflict history*, *Last election competitive*, and *Religious party in national assembly* are the same dichotomous indicators employed in Fig. 3. *GDP per capita*, *Religious fractionalization*, *Religious polarization* are contininuous measures: the underlying variables from which dichotomous subgroups are generated in Fig. 3. *Federalism* is not included due to high levels of missingness in the original measure.

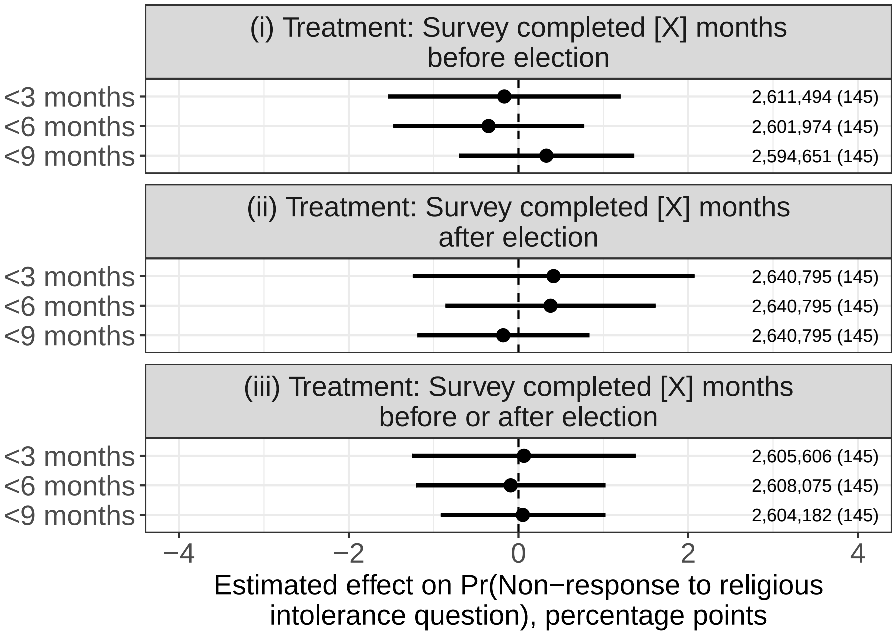

Figure S6: Coefficient plots of estimates from nine weighted least-squares regressions analyzing the effect of election proximity on the probability of respondents offering a non-response to a question about religious intolerance. Each regression employs a differently specified treatment variable, described in the panel titles and on the vertical axis. The unit of analysis is the respondent/question-item. Models include country, question-type, and question-target fixed effects. Only responses for which the survey date can be accurately pintpointed to within a six-month time bracket are included. Observations are weighted such that each country/election cycle contributes equally in estimation. 95 percent confidence intervals are based on robust standard errors clustered by country/election cycle. The number of observations, with the number of countries in parentheses, is displayed on the right-hand side of the plot.

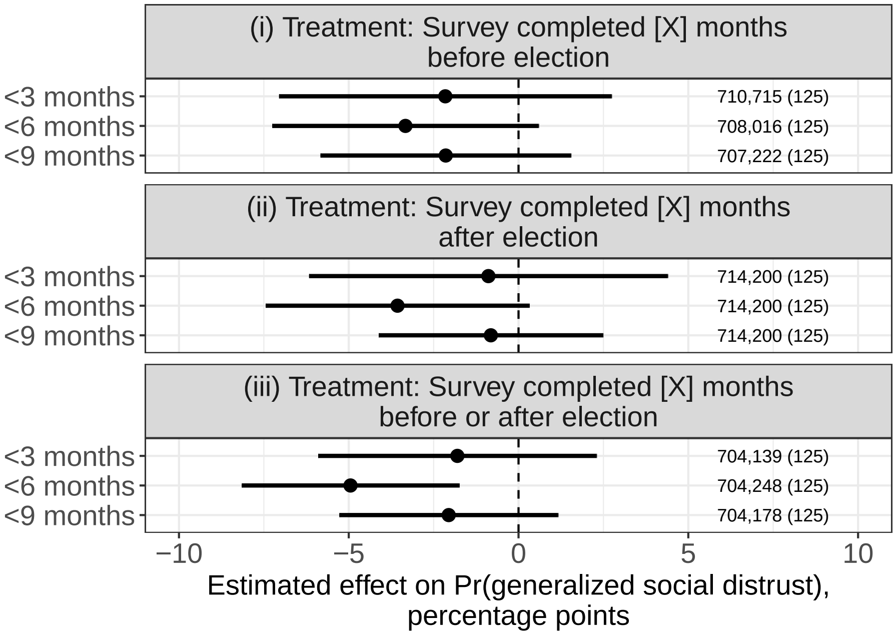

Figure S7: Complete results for generalized social distrust, for the full sample of respondents. Coefficient plots of estimates from nine weighted least-squares regressions. Each regression employs a differently specified treatment variable, described in the panel titles and on the vertical axis. The unit of analysis is the respondent. Models include country fixed effects. Only responses for which the survey date can be accurately pintpointed to within a six-month time bracket are included. Observations are weighted such that each country/election cycle contributes equally in estimation. 95 percent confidence intervals are based on robust standard errors clustered by country/election cycle. The number of observations, with the number of countries in parentheses, is displayed on the right-hand side of the plot.

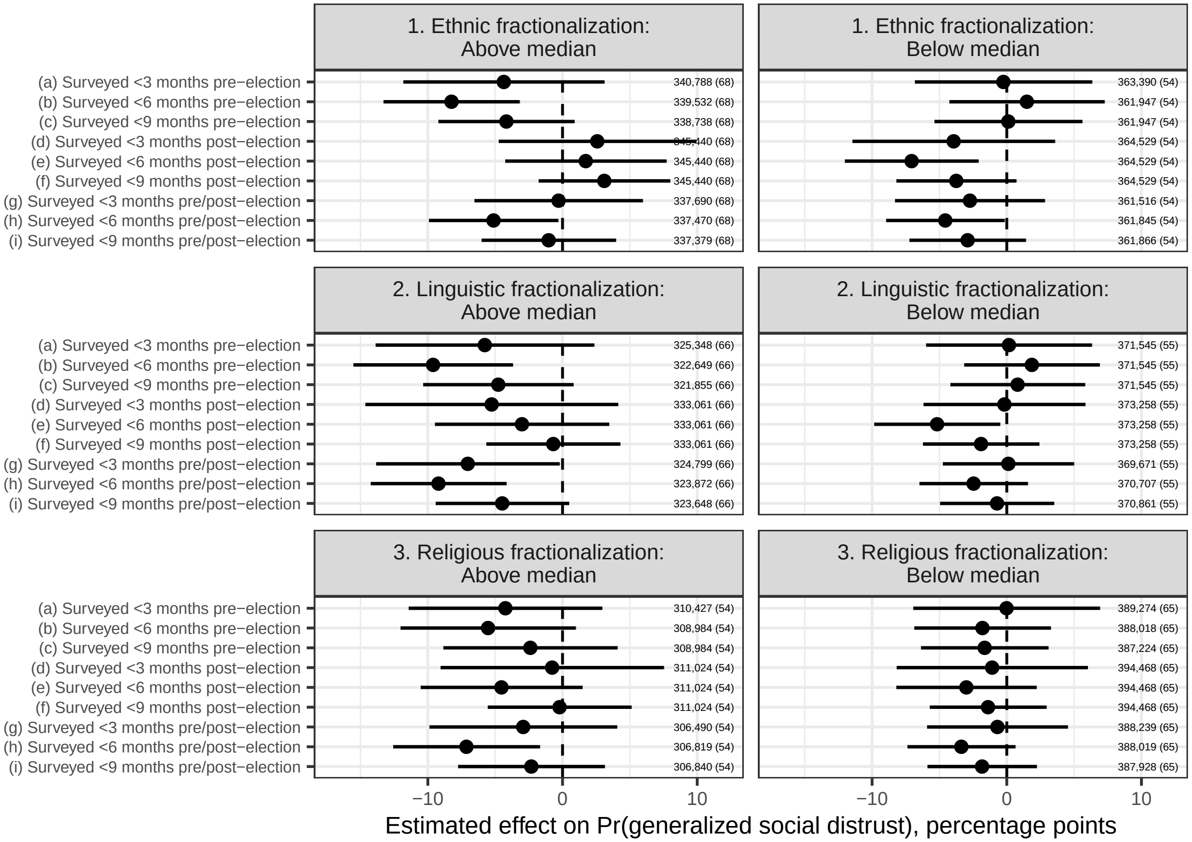

Figure S8: Subgroup effects for the generalized social distrust outcome. Coefficient plots of estimates from weighted least-squares regressions. Each regression employs a differently specified treatment variable, described on the vertical axis. The unit of analysis is the respondent. Models include country fixed effects. Only responses for which the survey date can be accurately pintpointed to within a six-month time bracket are included. Observations are weighted such that each country/election cycle contributes equally in estimation. 95 percent confidence intervals are based on robust standard errors clustered by country/election cycle. The number of observations, with the number of countries in parentheses, is displayed on the right-hand side of the plots.

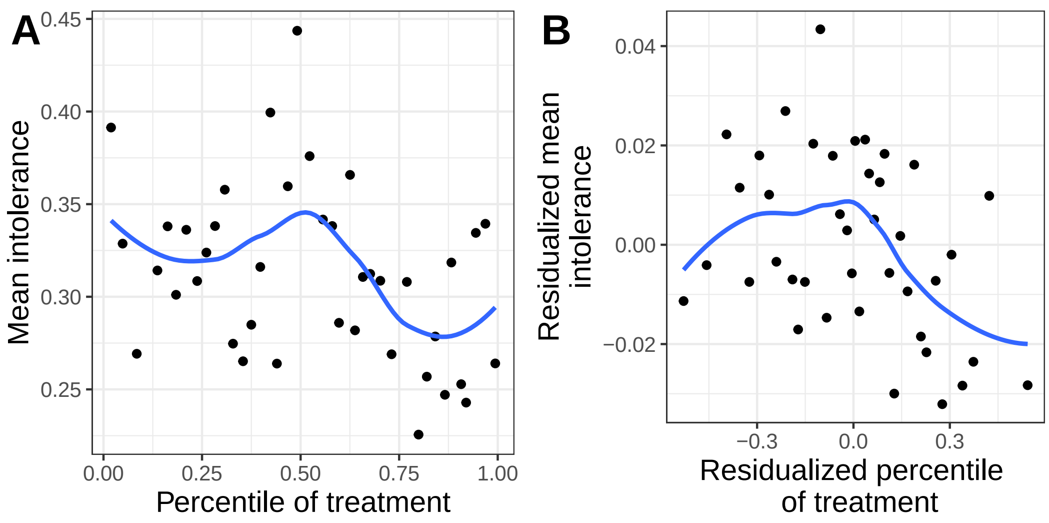

Figure S9: This figure presents binned scatter plots of the relationship between the stage at which individuals were surveyed in the course of the election cycle (in percentage terms) and whether they offered a negative attitude toward religious outgroups. In Panel A, I rank observations by their values on the treatment variable, and then split the data into 40 equally sized bins, according to this variable; next, I plot the bin-medians of the treatment variable against the bin-means of the outcome variable. Panel B follows the same proceduce, except both variables are first residualized to partial out country, question-type, and question-target fixed effects. Blue lines represent loess smoothers throught the binned data.

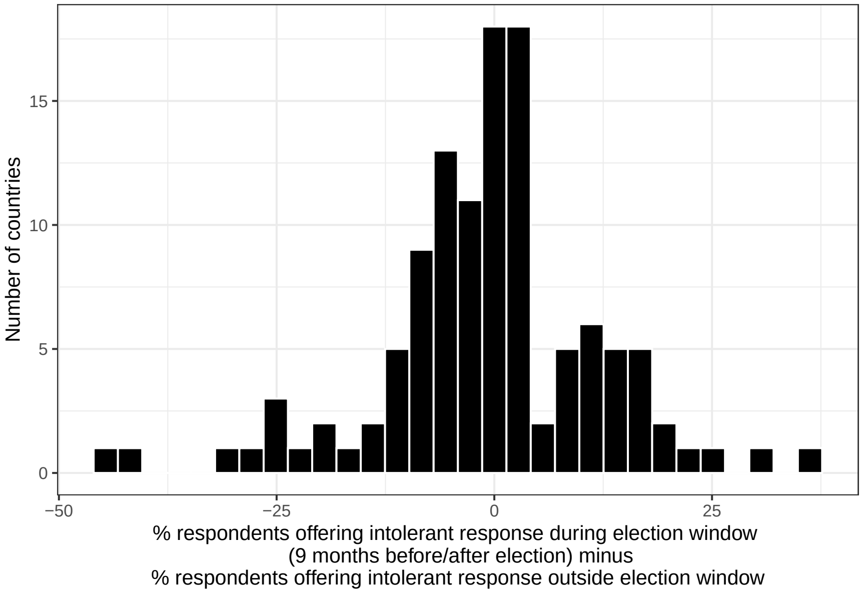

Figure S10: Histogram showing the distribution of the differences in mean intolerance rates between election periods and non-election periods, computed separately for each country.

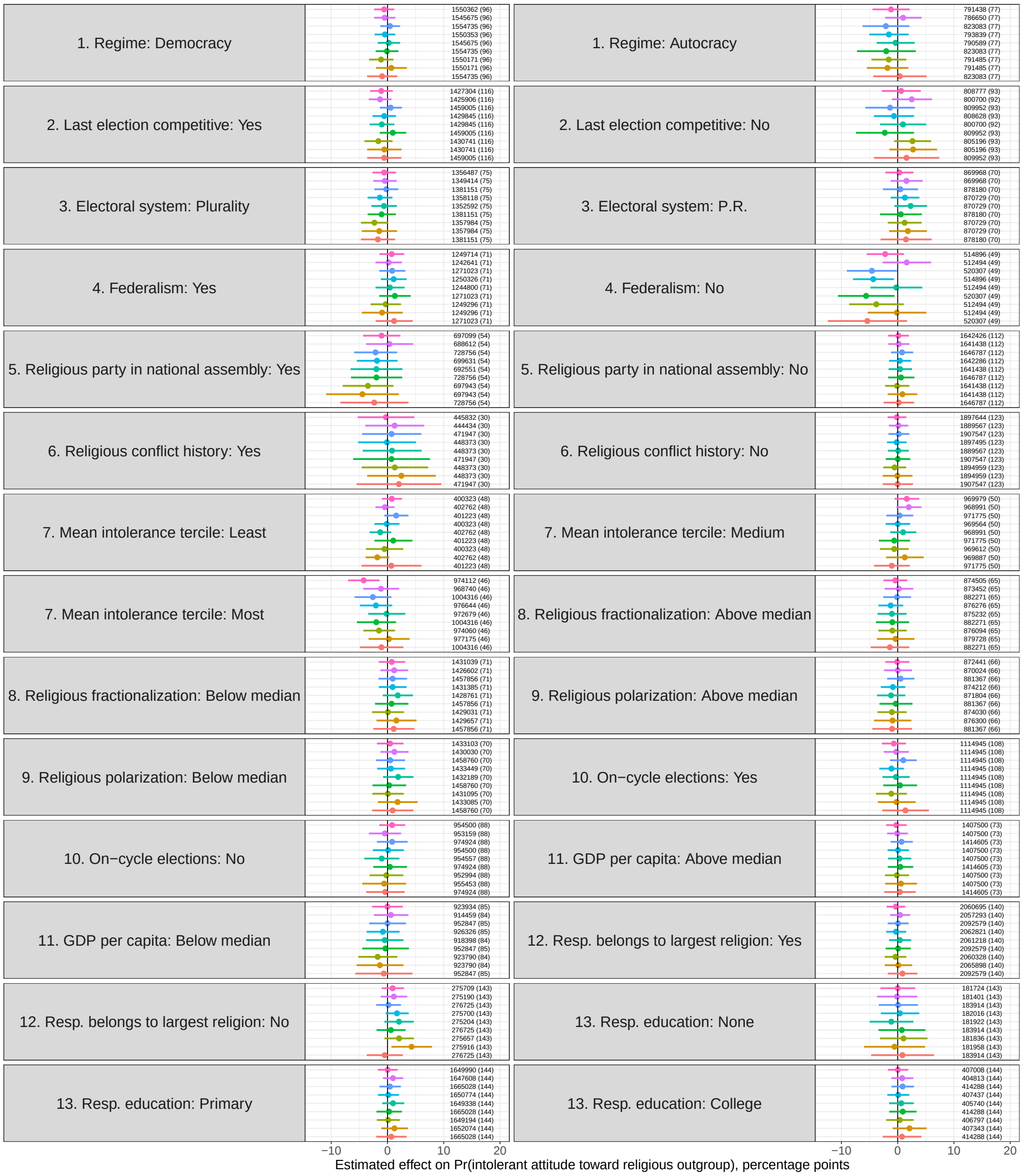

Figure S11: Complete subgroup effects. Estimates from weighted least-squares regressions that include country, question-type, and question-target fixed effects. Each subplot employs nine differently specified treatment variables (ordered as in Fig. 2). The unit of analysis is the respondent/question-item. Only responses for which the survey date can be accurately pintpointed to within a six-month time bracket are included. Observations are weighted such that each country/election cycle contributes equally in estimation. 95 percent confidence intervals are based on robust standard errors clustered by country/election cycle.

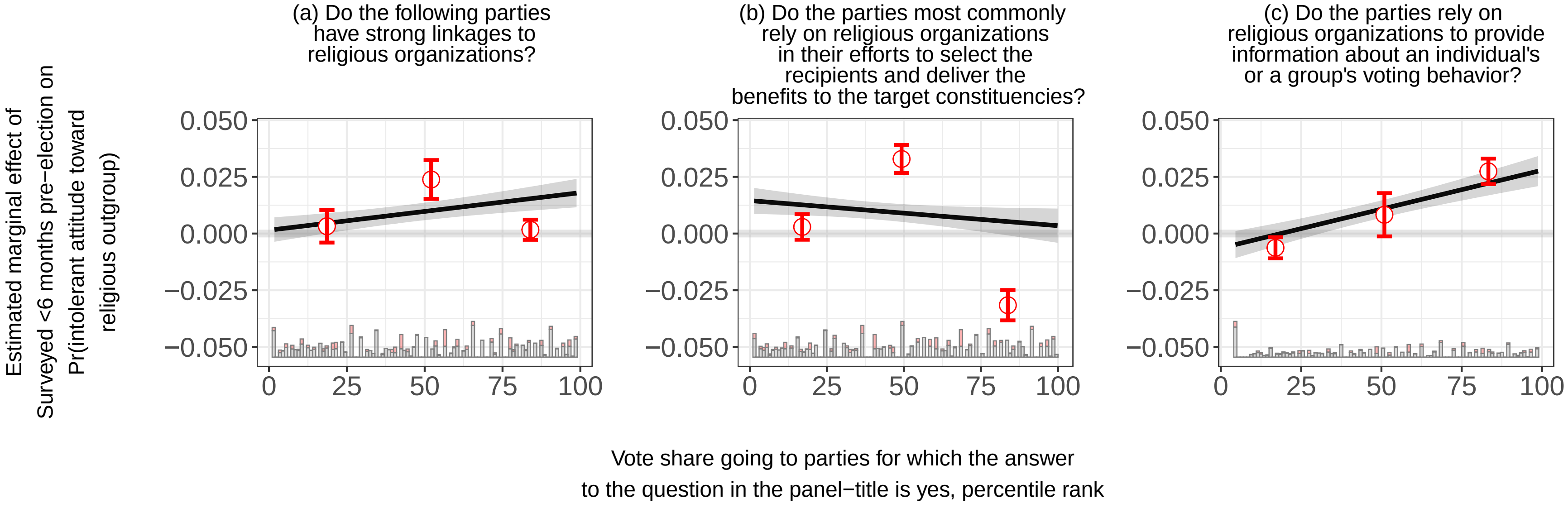

Figure S12: Estimated marginal effects according to the estimated national vote share received by parties with religious ties, as described in the Democratic Accountability and Linkages Project 2008. The unit of analysis is the respondent/question-item. Models include country, question-type, and question-target fixed effects. Only responses for which the survey date can be accurately pintpointed to within a six-month time bracket are included. Observations are weighted such that each country/election cycle contributes equally in estimation.

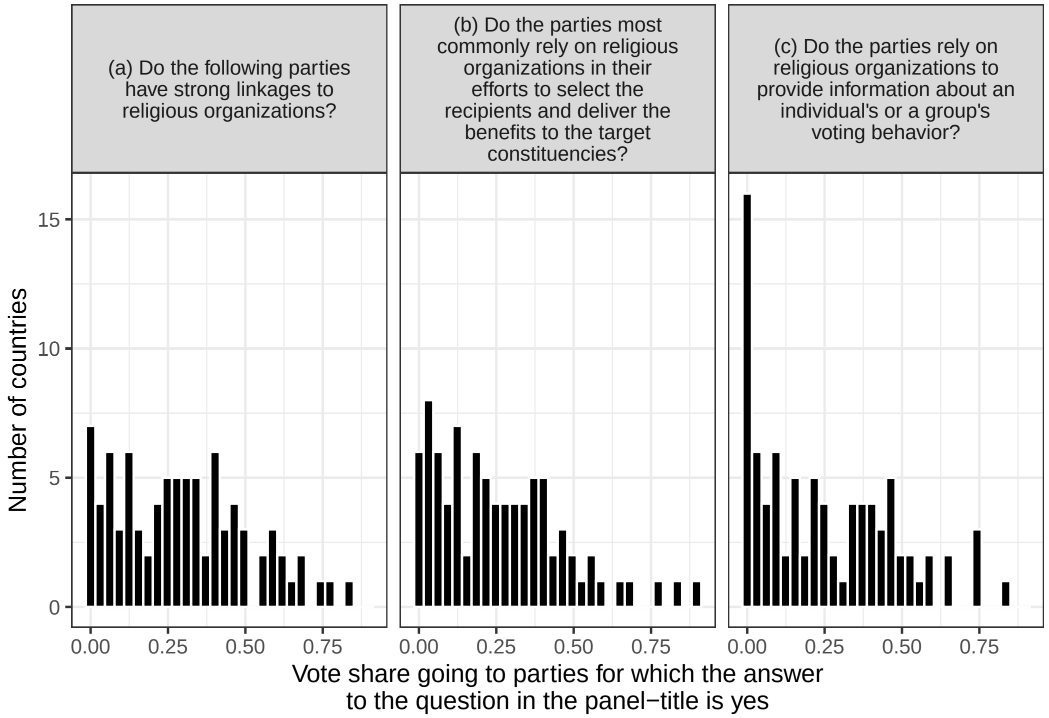

Figure S13: Histograms showing the distribution of vote shares received by parties with religious ties, as described in the Democratic Accountability and Linkages Project 2008.

B Additional tables

Table S1: Summary statistics. Detailed variable descriptions are provided in SI Appendix, Table S2. Variables are summarized at the level at which measurements were initially recorded.

Measure	N	Mean	St. Dev.	Min.	Median	Max.	Histogram
**Outcome (question-item level)**							
Intolerant attitude toward religious outgroup	2,576,234	0.31	0.46	0.00	0.00	1.00	
Generalized social distrust	821,742	0.66	0.47	0.00	1.00	1.00	
Religiosity	1,493,159	0.64	0.40	0.00	0.67	1.00	
Resp. has religion	1,503,683	0.84	0.37	0.00	1.00	1.00	
Non-response to religious intolerance question	2,859,490	0.10	0.30	0.00	0.00	1.00	
**Treatment variables (individual level)**							
Surveyed <3 months before election	1,180,596	0.09	0.28	0.00	0.00	1.00	
Surveyed <6 months before election	1,178,678	0.18	0.39	0.00	0.00	1.00	
Surveyed <9 months before election	1,176,283	0.26	0.44	0.00	0.00	1.00	
Surveyed <3 months after election	1,192,084	0.07	0.26	0.00	0.00	1.00	
Surveyed <6 months after election	1,192,084	0.14	0.35	0.00	0.00	1.00	
Surveyed <9 months after election	1,192,084	0.23	0.42	0.00	0.00	1.00	
Surveyed <3 months before/after election	1,176,820	0.15	0.36	0.00	0.00	1.00	
Surveyed <6 months before/after election	1,178,157	0.31	0.46	0.00	0.00	1.00	
Surveyed <9 months before/after election	1,176,923	0.45	0.50	0.00	0.00	1.00	
Percentage through election cycle surveyed	1,088,435	0.53	0.28	0.00	0.53	1.00	
**Country-level characteristics**							
Religious fractionalization above median	138	0.47	0.50	0.00	0.00	1.00	
Religious polarization above median	138	0.48	0.50	0.00	0.00	1.00	
Linguistic fractionalization above median	146	0.53	0.50	0.00	1.00	1.00	
Ethnic fractionalization above median	147	0.56	0.50	0.00	1.00	1.00	
**Country/election-level characteristics**							
Democracy	614	0.68	0.47	0.00	1.00	1.00	
Last election competitive	578	0.67	0.47	0.00	1.00	1.00	
Electoral system plurality	575	0.42	0.49	0.00	0.00	1.00	
Electoral system P.R.	575	0.58	0.49	0.00	1.00	1.00	
Federalism	445	0.71	0.46	0.00	1.00	1.00	
Religious party in national assembly	613	0.27	0.44	0.00	0.00	1.00	
Religious conflict history	617	0.15	0.36	0.00	0.00	1.00	
On-cycle elections	541	0.55	0.50	0.00	1.00	1.00	
GDP per capita above median	613	0.60	0.49	0.00	1.00	1.00	
**Individual-level characteristics**							
Female	1,224,770	0.53	0.50	0.00	1.00	1.00	
Rural	1,081,754	0.36	0.48	0.00	0.00	1.00	
Age	1,221,869	42.44	17.17	15.00	40.00	110.00	
Member of largest religion	1,220,228	0.87	0.34	0.00	1.00	1.00	
Education: None	1,126,043	0.10	0.30	0.00	0.00	1.00	

Education: Primary to college	1,126,043	0.73	0.44	0.00	1.00	1.00
Education: College	1,126,043	0.17	0.37	0.00	0.00	1.00
Surveyed in person	1,225,133	0.89	0.31	0.00	1.00	1.00

Table S2: Variables description and sources.

Variable	Data source(s)	Description
**Individual/question-item outcomes**		
• Intolerant attitude toward religious outgroup	Compiled multi-national surveys; see SI Appendix, Table S8.	Across the surveys, four types of questions appear probing respondents' attitudes toward members of religious outgroups: willingness to have members of different religious groups occupy social roles at varying "distances" to the respondent—as neighbors, work associates, friends, citizens, or family members—as well as trust, favoribility, and discomfort with respect to members of other religious groups. I recode responses dichotomously such that one always indicates that the respondent offered an intolerant attitude toward the religious group specified in the question wording, and zero indicates that the respondent offered a tolerant or neutral/indifferent attitude. All question-specific recodes are listed in SI Appendix, Table S12. Only responses for which the question inquired about a religion that was different from the religion with which the respondent identified are included in the dataset; this ensures that the dataset only captures attitudes toward religious outgroups.
• Generalized social distrust	Compiled multi-national surveys; see SI Appendix, Table S8.	I focus on the question: "Generally speaking, would you say that most people can be trusted or that you need to be very careful in dealing with people?" This question—or variants of it—is available in most, but not all, survey instruments. I recode responses dichotomously such that one always indicates that the respondent offered a negative attitude (i.e. expressed generalized distrust in other people), and zero indicates that the respondent offered a positive or neutral/indifferent attitude.
• Religiosity	Compiled multi-national surveys; see SI Appendix, Table S8.	Surveys probe the strength of individuals' personal religiosity in three ways: (i) by asking whether—or to what extent—the respondent considers themself to be "a religious person"; (ii) by asking how important religion is in the respondent's life; and (iii) by asking about the frequency with which a respondent engages in religious practices (praying, attending religious services). I employ one measure of religiosity per respondent. Where surveys contain more than one type of measure, I use type (i) if available, or otherwise types (ii) or (iii) in that order of preference. Most surveys provide likert-style response options (e.g. 1 = Not at all important, 2 = Somewhat unimportant, 3 = Somewhat important, 4 = Very important). For simplicity, I assume equal differences between ordered categories, and rescale variables to take a value between zero and one, where zero indicates "not at all religious" and one indicates "very religious."
• Resp. reports having any religion	Compiled multi-national surveys; see SI Appendix, Table S8.	This variable takes one if a respondent reported subscribing to any religion; it takes zero if the respondent reported being not religious (including if they reported being athiest or agnostic), or if they replied "don't know," "can't say," or refused to answer the question, or if the value for this variable is missing in the dataset for any other reason.

Table S2: (*continued*) Variables description and sources.

Variable	Data source(s)	Description
• Non-response to religious intolerance question	Compiled multi-national surveys; see SI Appendix, Table S8.	This variable takes one if a respondent offered a substantive response to a question about their attitude toward religious outgroups; it takes zero if the respondent replied "don't know," "can't say," or refused to answer the question, or if the value for this variable is missing in the dataset for any other reason.
**<u>Individual treatments</u>**		
• Surveyed [X] months before and/or after an election	Compiled multi-national surveys; see SI Appendix, Table S8. National Elections Across Democracy and Autocracy (NELDA) dataset; bit.ly/3Ng5YJo.	The dates on which individual respondents were interviewed are drawn from the original survey data. In instances where respondent-specific dates are not available, I input the individual's survey date as the midpoint of the range of dates during which surveys were fielded in that country/round; this information is provided in the surveys' technical documentation. For every individual, I then generate columns that contain the dates of the most recent (completed) and next upcoming national elections occuring in the country where the survey took place. Election dates are from the NELDA dataset. With this information merged, I then compute columns for (a) the number of days elapsed between the last election and the date on which the respondent was interviewed, and (b) the number of days between the date on which the respondent was interviewed and the next (forthcoming) election. Finally, I compute the suite of treatment indicators, dichotomously recording whether an individual's survey date falls within a given time window—variously specified as three, six, or nine months—of a previous and/or forthcoming election.
• Percentage way through election cycle surveyed	Compiled multi-national surveys; see SI Appendix, Table S8. National Elections Across Democracy and Autocracy (NELDA) dataset; bit.ly/3Ng5YJo.	For each individual, this continuous measure is computed as: (Number of days since last election)/(Total number of days between next election and last election). See the previous row of this table for a desciption of how the component variables were derived.
**<u>Individual characteristics</u>**		
• Female	Compiled multi-national surveys; see SI Appendix, Table S8.	The variable takes one if the respondent identifies as female and zero otherwise (unless missing). This information is available in almost all surveys.
• Rural	Compiled multi-national surveys; see SI Appendix, Table S8.	Most survey datasets contain a variable that captures the enumerator's assessment of the type of settlement where the interview took place. To standardize across instruments, I generate a dichotomous indicator for "Rural." This takes one in the following cases (and zero otherwise): (i) the terms "rural," "country," "countryside," or "village" are used to describe the interview location; (ii) 10,000 people or fewer are reported as living in the settlement; (iii) in cases where the binned response-options do not include the number 10,000 for the population of the settlement, then the population-bin with the upper bound above and closest to 10,000 is considered "rural." Settlements described as "refugee camps" are coded as not being rural.

Table S2: (*continued*) Variables description and sources.

Variable	Data source(s)	Description
• Education	Compiled multi-national surveys; see SI Appendix, Table S8.	Surveys record individuals' educational attainment in a plethora of ways. I assign each respondent to one of three educational attainment categories, which can be discerned quite consistently across datasets. I code individuals as having "No education" if they did not complete a country's formal primary school curriculum (noting that the length of the formal curriculum varies across countries); where that information in not provided, I code individuals with fewer than 5 years (grades) of formal education as having "No education." Individuals who report having completed an academic (not vocational) bachelors/undergraduate degree, or a more advanced academic degree, are recorded as having attained a "College" education; where only number of years of education is provided, I code individuals with 15 or more years of education as having a "College" education also. All remaining individuals—those who completed a primary school education but did not complete a college education, under these defintions—are coded has having "Primary" education, meaning that this group's educational attainment ranges from completed primary education up to and including incomplete college education. Each category receives a dichotomous indicator in the dataset.
• Age	Compiled multi-national surveys; see SI Appendix, Table S8.	Most surveys provide either respondents' precise age (in years), or their year of birth (from which age can be computed by knowing the year of the survey). Where surveys provide only a categorical age range (e.g. "27-35"), individuals' recoded age is imputed as the midpoint of the given range (thus, 31 in the "27-35" example). Where surveys provide categories that lack either an upper or lower bound (e.g. "75 and older," "20 or younger"), individuals' age is imputed as the *given* bound (thus, 75 and 20, respectively, in the examples just cited).
• Member of largest religion	Cross-National Socio-Economic and Religion (CNSER) Data, 2011; bit.ly/3zmGXIm.	I use the RELRECOD variable from the CNSER data to code the largest religion for each country as Christian, Muslim, Jewish, Hindu, Buddhist, Shinto, or Other religion. I then create an indicator that takes one (and zero otherwise) in cases where a surveyed individual's self-identified religion matches the largest religion in the country where they reside.
• Surveyed in person	Compiled multi-national surveys; see SI Appendix, Table S8.	Technical survey documentation and, in several cases, dataset variables were used to create an indicator that takes one if the respondent was interviewed face-to-face, in-person by an enumerator, and zero otherwise.
**Country/election-level characteristics**		
• Regime: Democracy, Autocracy	Varietes of Democracy (V-DEM) Project, version 12; bit.ly/3NcHP6t.	Variable derived from the V-DEM item, v2xregime: "How can the political regime overall be classified considering the competitiveness of access to power (polyarchy) as well as liberal principles?" The original V-DEM variable is coded for country/years and takes four levels. In my recode, I class "Autocracy" as comprising either the level "Closed autocracy" or "Electoral autocracy" in the original coding scheme, and "Democracy" as comprising either "Electoral democracy" or "Liberal democracy" in the original coding scheme.

Table S2: (*continued*) Variables description and sources.

Variable	Data source(s)	Description
• Electoral system: Plurality, P.R.	Database of Political Institutions (DPI) 2020; bit.ly/3Tn5JiM.	I use the variable HOUSESYS from the DPI dataset, which codes the electoral rule (proportional representation or plurality) governing the election of the majority of national "house seats" in a given country/year. I generate a variable that takes one if the value of HOUSESYS is "Plurality" for a given country/year, and zero otherwise. I generate a second variable that takes one if the value of HOUSESYS is "Proportional representation (P.R.)" for a given country/year, and zero otherwise.
• Federalism	Database of Political Institutions (DPI) 2020; bit.ly/3Tn5JiM.	I use the variable STATE from the DPI dataset, which codes whether state/provincil governments are locally elected or not for a given country/year. I generate a variable that takes zero if the value of STATE is zero (denoting that state/provincial governments are not elected), and one if the value of STATE is one or two (respectively denoting that the state/provincial legislature, or the state/provincial executive and legislature, are elected).
• Religious party in national assembly	Varieties of Party Identity and Organization (V-Party) Dataset; bit.ly/3MbpGVt.	The V-Party dataset consists of expert codings of the characteristics of parties attaining more than five percent of seats in a country's national assembly. The original unit of analysis is the country/election year/party. I use the variable, v2parelig: "To what extent does this party invoke God, religion, or sacred/religious texts to justify its positions?" I classify a religious party as one that is coded as "Always or almost always" or "Often, but not always" invoking God, religion, or sacred/religious texts to justify its positions. Finally, I generate a variable that takes one if there is any religious party in the national assembly in a given country/election year (and zero otherwise). To make a country/year panel, I fill this variable forward annually within country, until the next election is reached.
• Last election competitive	National Elections Across Democracy and Autocracy (NELDA) dataset; bit.ly/3Ng5YJo.	Variable based on NELDA 12, which is dichotomous: "Was the incumbent or ruling party confident of victory before elections?" In the original dataset, "no" indicates that the incumbent was not confident of victory, indicating that the election was at least somewhat competitive. The final analysis variable takes one if the last election was coded as competitive according to this measure, and zero otherwise.

Table S2: (*continued*) Variables description and sources.

Variable	Data source(s)	Description
• Religious conflict history	Religion and Armed Conflict (RELAC) Data; bit.ly/3nh49kk.	The RELAC dataset is an extension of the UCDP Dyadic Dataset of armed conflicts in the world between 1975 and 2015. Armed conflicts are defined as: "a contested incompatibility that concerns government and/or territory where the use of armed force between two parties, of which at least one is the government of a state, results in at least 25 battle-related deaths in a calendar year." RELAC adds a newly coded column (RelIncomp) for "Religious Incompatability," capturing "whether there is a religious dimension in the original incompatibility as explicitly stated at the onset of the conflict by the representatives of the primary parties." The dataset also provides a variable for the start date of each conflict. I keep all conflicts for which RelIncomp takes a value of one. Finally, in the analysis dataset, I generate a dichotomous variable indicating whether, at the time of the last election (in relation to when the respondent was surveyed), a country had previously experienced a religious conflict according to the RELAC dataset. Note, therefore, that this measure does not capture any conflicts that occurred prior to 1975.
• On-cycle elections	National Elections Across Democracy and Autocracy (NELDA) dataset; bit.ly/3Ng5YJo.	Variable based on NELDA 6, which is dichotomous: "If regular, were these elections early or late relative to the date they were supposed to be held per established procedure?" In the original dataset, "no" indicates that the election was held on-cycle. The final analysis variable takes one if both the last and next elections (relative to the date of the individual survey) were held on-cycle, and zero otherwise.
• GDP per capita above median	United Nations Statistics Division; bit.ly/3ze9Pm8	The UN provides a dataset of GDP per capita—at current prices in US Dollars—for all countries except Taiwan between 1970 and 2020. I limit the series to the years 1975 to 2020, which is the date-range of the first to last election that appears in the analysis dataset. For each year, I then categorize each country as above or below the median per-capita income for all countries in that given year.
• Election involved civilian deaths	National Elections Across Democracy and Autocracy (NELDA) dataset; bit.ly/3Ng5YJo.	Variable based on NELDA 33, which is dichotomous: "Was there significant violence involving civilian deaths immediately before, during, or after the election?" The analysis variable takes one if there was such violence and zero otherwise.
**Country-level characteristics**		
• Mean intolerance tercile	Compiled multi-national surveys; see SI Appendix, Table S8.	I take the average rate of religious intolerance toward religious outgroups by country, using the integrated survey dataset, across all available years. I then assign countries into terciles of intolerance using the ranking of these country-level averages.
• Religious fractionalization above median	Composition of Religious and Ethnic Groups Fractionalization/Polarization Estimates dataset, produced by Steven V. Miller; bit.ly/3grYjfy.	I use the religious fractionalization measure from the Steven V. Miller dataset. The measure is an application of the Herfindahl-Hirschman concentration index, based on cross-national religious demographic data for the year 2000. I use the median value for the full set of countries to create a dichotomous varaible indicating that a country's value was above median.

Table S2: (*continued*) Variables description and sources.

Variable	Data source(s)	Description
• Religious polarization above median	Composition of Religious and Ethnic Groups Fractionalization/Polarization Estimates dataset, produced by Steven V. Miller; bit.ly/3grYjfy.	I use the religious polarization measure from the Steven V. Miller dataset dataset. The measure is an application of the Montalvo and Reynal-Querol polarization index, introduced in Jose Montalvo and Marta Reynal-Querol (2005), "Ethnic polarization, potential conflict, and civil wars," American Economic Review 95(3), pp. 796–816. The calculation is based on cross-national religious demographic data for the year 2000. I use the median value for the full set of countries to create a dichotomous varaible indicating that a country's value was above median.
• Linguistic fractionalization above median	Alberto Alesina et al (2003). "Fractionalization." Journal of Economic Growth 8, pp. 155–194; bit.ly/3bl0Rtr.	I use the linguistic fractionalization measure from the original Alesina et al dataset. The measure is an application of the Herfindahl concentration index, based on data from various sources. I use the median value for the full set of countries to partition the data into above and below median values.
• Ethnic fractionalization above median	Alberto Alesina et al (2003). "Fractionalization." Journal of Economic Growth 8, pp. 155–194; bit.ly/3bl0Rtr.	I use the ethnic fractionalization measure from the original Alesina et al dataset. The measure is an application of the Herfindahl concentration index, based on data from various sources. I use the median value for the full set of countries to partition the data into above and below median values.
**Fixed effects**		
• Country		Fixed effects for each country.
• Question-type		Fixed effects for the question types that describe the nature of the question asked regarding religious intolerance (various social roles, trust, favorability, discomfort).
• Question-target		Fixed effects for the religion or sect specified in the question text for questions about religious intolerance, including for aggregate target groups such as "Different religion" and "Different sect."

Table S3: Weighted least-squares regression estimates of balance on individuals' pre-treatment characteristics. The unit of analysis is the respondent. Q-type and Q-target refer to question-type and question-target, respectively. Only responses for which the survey date can be accurately pintpointed to within a six-month time bracket are included. Observations are weighted such that each country/election cycle contributes equally in estimation. Robust standard errors clustered by country/election cycle are in parentheses.

Outcome:	Female<br>(1)	Age<br>(2)	Rural<br>(3)	Belongs to largest religion<br>(4)	Education: None<br>(5)	Education: Primary<br>(6)	Education: College<br>(7)	Surveyed in person<br>(8)
**Panel A**								
Surveyed <3 months pre-election	0.008	0.073	-0.008	0.001	-0.007	0.017	-0.010	0.029
	(0.008)	(0.610)	(0.016)	(0.007)	(0.009)	(0.012)	(0.009)	(0.018)
N	1,131,258	1,129,095	996,486	1,126,688	1,049,224	1,049,224	1,049,224	1,131,524
R^2	0.01	0.13	0.10	0.36	0.19	0.07	0.06	0.51
Country + Q-type + Q-target FE	Y	Y	Y	Y	Y	Y	Y	Y
**Panel B**								
Surveyed <6 months pre-election	0.001	-0.158	-0.012	0.000	0.000	0.011	-0.012*	0.012
	(0.006)	(0.503)	(0.013)	(0.007)	(0.007)	(0.009)	(0.007)	(0.016)
N	1,129,340	1,127,177	994,568	1,124,770	1,048,323	1,048,323	1,048,323	1,129,606
R^2	0.01	0.13	0.10	0.36	0.19	0.07	0.06	0.51
Country + Q-type + Q-target FE	Y	Y	Y	Y	Y	Y	Y	Y
**Panel C**								
Surveyed <9 months pre-election	0.004	0.159	-0.010	-0.003	0.000	0.012	-0.012*	-0.010
	(0.006)	(0.452)	(0.012)	(0.006)	(0.006)	(0.008)	(0.006)	(0.015)
N	1,126,945	1,124,782	992,173	1,122,375	1,046,689	1,046,689	1,046,689	1,127,211
R^2	0.01	0.13	0.10	0.36	0.19	0.07	0.06	0.51
Country + Q-type + Q-target FE	Y	Y	Y	Y	Y	Y	Y	Y
**Panel D**								
Surveyed <3 months post-election	0.010	0.592	-0.004	0.013	0.006	-0.012	0.007	0.035
	(0.014)	(0.779)	(0.020)	(0.009)	(0.007)	(0.012)	(0.011)	(0.024)
N	1,142,764	1,140,564	1,007,717	1,138,176	1,053,570	1,053,570	1,053,570	1,143,012
R^2	0.01	0.13	0.10	0.36	0.19	0.07	0.06	0.48
Country + Q-type + Q-target FE	Y	Y	Y	Y	Y	Y	Y	Y
**Panel E**								
Surveyed <6 months post-election	0.004	0.404	-0.003	0.013*	0.003	-0.013	0.010	0.021
	(0.009)	(0.533)	(0.015)	(0.007)	(0.006)	(0.009)	(0.008)	(0.020)
N	1,142,764	1,140,564	1,007,717	1,138,176	1,053,570	1,053,570	1,053,570	1,143,012
R^2	0.01	0.13	0.10	0.36	0.19	0.07	0.06	0.48
Country + Q-type + Q-target FE	Y	Y	Y	Y	Y	Y	Y	Y
**Panel F**								
Surveyed <9 months post-election	0.003	-0.042	0.004	0.001	0.003	-0.015**	0.012*	0.015
	(0.006)	(0.449)	(0.012)	(0.006)	(0.005)	(0.007)	(0.006)	(0.017)
N	1,142,764	1,140,564	1,007,717	1,138,176	1,053,570	1,053,570	1,053,570	1,143,012
R^2	0.01	0.13	0.10	0.36	0.19	0.07	0.06	0.48
Country + Q-type + Q-target FE	Y	Y	Y	Y	Y	Y	Y	Y
**Panel G**								
Surveyed <3 months pre/post-election	0.005	0.187	-0.006	0.003	-0.002	-0.001	0.002	0.026
	(0.008)	(0.534)	(0.014)	(0.007)	(0.007)	(0.010)	(0.008)	(0.017)
N	1,127,493	1,125,335	992,716	1,122,912	1,046,216	1,046,216	1,046,216	1,127,748
R^2	0.01	0.13	0.10	0.36	0.19	0.07	0.06	0.51
Country + Q-type + Q-target FE	Y	Y	Y	Y	Y	Y	Y	Y
**Panel H**								
Surveyed <6 months pre/post-election	0.001	0.066	-0.005	0.007	0.000	-0.001	0.001	0.019
	(0.005)	(0.433)	(0.012)	(0.006)	(0.006)	(0.008)	(0.006)	(0.015)
N	1,128,830	1,126,669	994,053	1,124,249	1,047,418	1,047,418	1,047,418	1,129,085
R^2	0.01	0.13	0.10	0.36	0.19	0.07	0.06	0.50
Country + Q-type + Q-target FE	Y	Y	Y	Y	Y	Y	Y	Y
**Panel I**								
Surveyed <9 months pre/post-election	0.005	-0.038	-0.001	-0.001	-0.003	-0.001	0.004	-0.001
	(0.005)	(0.406)	(0.012)	(0.006)	(0.006)	(0.007)	(0.006)	(0.013)
N	1,127,596	1,125,435	992,813	1,123,015	1,046,796	1,046,796	1,046,796	1,127,851
R^2	0.01	0.13	0.10	0.36	0.19	0.07	0.06	0.50
Country + Q-type + Q-target FE	Y	Y	Y	Y	Y	Y	Y	Y

*p<0.1; **p<0.05; ***p<0.01

Table S4: Weighted least-squares regression estimates of the relationship between being surveyed within specified time-frames before and after a national election and expressions of intolerance toward religious outgroups. This table presents the results of nine separate regression models. Indicators for being surveyed before and after an election are entered simultaneously into the regression models. To assess robustness, different combinations of fixed effects are included, as indicated in the table header. Only responses for which the survey date can be accurately pintpointed to within a six-month time bracket are included. In each model, observations are weighted such that each country/election cycle contributes equally in estimation. Robust standard errors clustered by country/election cycle are in parentheses.

Country FE:	Y	Y	Y
Question-type FE:	N	Y	Y
Question-target FE:	N	N	Y
	(1)	(2)	(3)
**Panel A: Treatment window = 3 months**			
Surveyed <3m pre-election	0.018	0.004	0.003
	(0.018)	(0.012)	(0.012)
Surveyed <3m post-election	0.013	0.007	0.002
	(0.019)	(0.013)	(0.013)
N	2,343,332	2,343,332	2,343,332
R^2	0.11	0.17	0.18
**Panel B: Treatment window = 6 months**			
Surveyed <6m pre-election	0.015	0.001	0.004
	(0.013)	(0.009)	(0.009)
Surveyed <6m post-election	0.006	0.008	0.004
	(0.016)	(0.011)	(0.011)
N	2,337,940	2,337,940	2,337,940
R^2	0.11	0.17	0.18
**Panel C: Treatment window = 9 months**			
Surveyed <9m pre-election	0.011	0.001	0.005
	(0.012)	(0.009)	(0.008)
Surveyed <9m post-election	0.007	0.006	0.005
	(0.013)	(0.009)	(0.009)
N	2,334,001	2,334,001	2,334,001
R^2	0.11	0.17	0.18

*p<0.1; **p<0.05; ***p<0.01

Table S5: Tabulation of primary results displayed in Figure 2 of the main paper. Weighted least-squares regression estimates of the relationship between being surveyed within a specified time-frame before and/or after a national election and expressions of intolerance toward religious outgroups. The unit of analysis is the respondent/question-item. Models include country, question-type, and question-target fixed effects. Only responses for which the survey date can be accurately pintpointed to within a six-month time bracket are included. Observations are weighted such that each country/election cycle contributes equally in estimation. Standard errors are clustered by country/election cycle, and 95 percent confidence intervals are based on these.

Treatment	Estimate	S.E.	C.I.	p-value	Sig.	N	Countries
Surveyed <3 months pre-election	0.004	0.012	(-0.020, 0.028)	0.741		2,352,004	145
Surveyed <6 months pre-election	0.003	0.009	(-0.015, 0.022)	0.710		2,346,612	145
Surveyed <9 months pre-election	0.004	0.009	(-0.013, 0.020)	0.682		2,342,673	145
Surveyed <3 months post-election	0.003	0.013	(-0.022, 0.028)	0.812		2,379,494	145
Surveyed <6 months post-election	0.001	0.011	(-0.020, 0.022)	0.917		2,379,494	145
Surveyed <9 months post-election	0.003	0.009	(-0.015, 0.020)	0.780		2,379,494	145
Surveyed <3 months pre-/post-election	-0.004	0.009	(-0.023, 0.014)	0.645		2,346,175	145
Surveyed <6 months pre-/post-election	-0.002	0.008	(-0.018, 0.015)	0.855		2,348,711	145
Surveyed <9 months pre-/post-election	-0.002	0.008	(-0.018, 0.014)	0.797		2,346,594	145

*p<0.1; **p<0.05; ***p<0.01

Table S6: Tabulation of subgroup effects displayed in Figure 3 of the main paper. Weighted least-squares regression estimates of the relationship between being surveyed six months before a national election and expressions of intolerance toward religious outgroups. Models include country, question-type, and question-target fixed effects. The unit of analysis is the respondent/question-item. Only responses for which the survey date can be accurately pintpointed to within a six-month time bracket are included. Observations are weighted such that each country/election cycle contributes equally in estimation. Standard errors are clustered by country/election cycle, and 95 percent confidence intervals are based on these.

Subgroup	Estimate	S.E.	C.I.	p-value	Sig.	N	Countries
**1. Regime**							
Democracy	0.002	0.010	(-0.017, 0.022)	0.824		1,545,675	96
Autocracy	-0.004	0.017	(-0.037, 0.029)	0.818		790,589	77
**2. Last election competitive**							
Yes	-0.010	0.011	(-0.032, 0.011)	0.352		1,429,845	116
No	0.009	0.021	(-0.031, 0.050)	0.646		800,700	92
**3. Electoral system**							
Plurality	-0.006	0.011	(-0.029, 0.016)	0.571		1,352,592	75
P.R.	0.023	0.014	(-0.005, 0.051)	0.112		870,729	70
**4. Federalism**							
Yes	0.004	0.013	(-0.021, 0.030)	0.736		1,244,800	71
No	-0.003	0.023	(-0.048, 0.043)	0.909		512,494	49
**5. Religious party in national assembly**							
Yes	-0.020	0.023	(-0.065, 0.026)	0.391		692,551	54
No	0.004	0.010	(-0.016, 0.025)	0.673		1,641,438	112
**6. Religious conflict history**							
Yes	0.008	0.026	(-0.044, 0.060)	0.757		448,373	30
No	0.001	0.009	(-0.017, 0.019)	0.919		1,889,567	123
**7. Mean intolerance tercile**							
Least	-0.013	0.009	(-0.031, 0.006)	0.173		402,762	48
Medium	0.010	0.011	(-0.013, 0.032)	0.403		968,991	50
Most	-0.002	0.016	(-0.034, 0.031)	0.926		972,679	46
**8. Religious fractionalization**							
Above median	-0.010	0.013	(-0.036, 0.015)	0.423		875,232	65
Below median	0.018	0.013	(-0.008, 0.045)	0.172		1,428,761	71
**9. Religious polarization**							
Above median	-0.012	0.012	(-0.036, 0.013)	0.345		871,804	66
Below median	0.019	0.014	(-0.008, 0.045)	0.163		1,432,189	70
**10. On-cycle elections**							
Yes	-0.003	0.012	(-0.027, 0.021)	0.800		1,114,945	108
No	-0.010	0.016	(-0.041, 0.020)	0.510		954,557	88
**11. GDP per capita**							
Above median	0.003	0.010	(-0.017, 0.023)	0.755		1,407,500	73
Below median	-0.005	0.017	(-0.038, 0.028)	0.758		918,398	84
**12. Resp. belongs to largest religion**							
Yes	0.004	0.010	(-0.015, 0.023)	0.685		2,061,218	140
No	0.020	0.013	(-0.005, 0.046)	0.120		275,204	143
**13. Resp. education**							
None	-0.011	0.020	(-0.050, 0.027)	0.562		181,922	143
Primary	0.010	0.010	(-0.009, 0.029)	0.315		1,649,338	144
College	0.006	0.011	(-0.015, 0.028)	0.563		405,740	144

*p<0.1; **p<0.05; ***p<0.01

Table S7: Subgroup effects, equivalent to those displayed in Figure 3 of the main paper, but for *democratic countries only.* Weighted least-squares regression estimates of the relationship between being surveyed six months before a national election and expressions of intolerance toward religious outgroups. Models include country, question-type, and question-target fixed effects. The unit of analysis is the respondent/question-item. Only responses for which the survey date can be accurately pintpointed to within a six-month time bracket are included. Observations are weighted such that each country/election cycle contributes equally in estimation. Standard errors are clustered by country/election cycle, and 95 percent confidence intervals are based on these.

Subgroup	Estimate	S.E.	C.I.	p-value	Sig.	N	Countries
**1. Last election competitive**							
Yes	0.000	0.012	(-0.023, 0.023)	0.990		1,108,468	85
No	0.007	0.022	(-0.037, 0.050)	0.756		393,263	46
**2. Electoral system**							
Plurality	-0.003	0.012	(-0.027, 0.021)	0.782		1,089,976	60
P.R.	0.011	0.014	(-0.017, 0.040)	0.431		445,001	39
**3. Federalism**							
Yes	0.015	0.013	(-0.010, 0.040)	0.230		1,020,920	53
No	-0.053	0.028	(-0.109, 0.003)	0.065	*	243,228	29
**4. Religious party in national assembly**							
Yes	0.005	0.027	(-0.049, 0.059)	0.850		388,098	29
No	0.000	0.011	(-0.021, 0.021)	0.971		1,157,577	81
**5. Religious conflict history**							
Yes	-0.049	0.056	(-0.165, 0.067)	0.394		147,181	9
No	0.006	0.010	(-0.014, 0.025)	0.556		1,398,494	89
**6. Mean intolerance tercile**							
Least	-0.015	0.010	(-0.034, 0.004)	0.119		331,328	38
Medium	0.011	0.013	(-0.014, 0.036)	0.374		759,608	34
Most	-0.003	0.020	(-0.044, 0.037)	0.868		453,646	23
**7. Religious fractionalization**							
Above median	0.000	0.015	(-0.030, 0.029)	0.987		508,449	37
Below median	0.013	0.013	(-0.012, 0.039)	0.300		1,007,996	53
**8. Religious polarization**							
Above median	-0.007	0.014	(-0.034, 0.021)	0.646		524,965	39
Below median	0.016	0.013	(-0.010, 0.042)	0.218		991,480	51
**9. On-cycle elections**							
Yes	0.001	0.013	(-0.024, 0.026)	0.928		840,853	75
No	0.005	0.017	(-0.030, 0.039)	0.782		578,120	58
**10. GDP per capita**							
Above median	0.008	0.010	(-0.013, 0.028)	0.462		1,177,521	56
Below median	-0.017	0.027	(-0.070, 0.036)	0.522		356,112	45
**11. Resp. belongs to largest religion**							
Yes	0.000	0.010	(-0.020, 0.020)	0.977		1,375,279	94
No	0.011	0.014	(-0.016, 0.038)	0.427		160,622	96
**12. Resp. education**							
None	0.002	0.025	(-0.048, 0.052)	0.940		87,424	95
Primary	0.005	0.011	(-0.016, 0.026)	0.619		1,114,518	95
College	-0.002	0.012	(-0.026, 0.021)	0.859		288,457	95

*p<0.1; **p<0.05; ***p<0.01

Table S8: List and description of included survey series.

Source	Link to original data	Brief description	Target sample
A Cross-National Survey of Muslim Attitudes	bit.ly/3z8Wdsm	"The survey of Muslims was conducted on behalf of Arizona State University by Abt SRBI in Southeast Asia, West Africa, and Western Europe. The goal of the study is to investigate how Muslim individuals and communities respond to and counter radical or extremist actors in their discourses. It details religious attitudes and perceptions of the impact of the West on Muslim societies."	Muslims only
Afrobarometer	bit.ly/3POesJs	"A pan-African, non-partisan survey research network that conducts public attitude surveys on democracy, governance, the economy, and society ... Our network encompasses more than 30 national partners responsible for data collection, analysis, and in-country dissemination of findings."	Nationally representative
Arab Barometer	bit.ly/3sYWE4L	"Arab Barometer is a nonpartisan research network that provides insight into the social, political, and economic attitudes and values of ordinary citizens across the Arab world. We have been conducting high quality and reliable public opinion surveys in the Middle East and North Africa since 2006. We are the longest-standing and the largest repository of publicly available data on the views of men and women in the MENA region."	Nationally representative
Arab Opinion Index	bit.ly/3BW50O3	"The Arab Opinion Index [is] an annual survey conducted by the Arab Center in order to gauge Arab public opinion around a number of political, cultural, and social topics. The program is headed by a specialist statistician, leading a team of research assistants and experts in survey methods."	Nationally representative
Candidate Eurobarometer	bit.ly/3t46CBQ	"In October 2001, the European Commission launched a new series of surveys in the 13 countries that are applying for European Union membership under the heading Candidate Countries Eurobarometer ... The CCEB surveys gather information from the societies applying to become members of the EU in a way that is comparable with the Standard Eurobarometer."	Nationally representative
Caucasus Barometer	bit.ly/3LVCeAm	"The Caucasus Barometer is an annually conducted nationwide survey in Georgia, Armenia and Azerbaijan that covers a wide range of social, economic and political issues in the region." It is run by the Caucasus Research Resource Centers.	Nationally representative
Comparative Cross-National Study of Religious Fundamentalism, Developmental Idealism, Values, and Morality in the Middle East and North Africa	bit.ly/3lTiV00	Part of the Middle Eastern Values Study. "The objective of Middle Eastern Values Studies (MEVS) is to advance the social-scientific understanding of the causes and consequences of the values and the belief systems of the people of the Middle East ... The Middle East Value Studies began with a three-country survey ... in 2000-2001."	Nationally representative
Comparative Panel Survey on the Dynamics of Change: Belief Formation and Political Engagement in Egypt, Tunisia, and Turkey	bit.ly/3lSnAiB	Part of the Middle Eastern Values Study. "The objective of Middle Eastern Values Studies (MEVS) is to advance the social-scientific understanding of the causes and consequences of the values and the belief systems of the people of the Middle East ... The Middle East Value Studies began with a three-country survey ... in 2000-2001."	Nationally representative
CROss-National Online Survey	bit.ly/3URDxpy	"CRONOS was designed and implemented alongside ESS Round 8 in 2016, as part of the larger SERISS project. After completing the ESS face-to-face interview, respondents in Estonia, Great Britain and Slovenia 18 or older were invited to participate in six 20-minute online surveys over a time period of twelve months."	Nationally representative
Eurobarometer	bit.ly/3POLMQJ; bit.ly/3wZktug; bit.ly/3a30naM; bit.ly/3z6D1eI; bit.ly/3lWZIdQ; bit.ly/3z78GwS; bit.ly/3aji5XA; bit.ly/3zbIH7z	"Eurobarometer is a series of public opinion surveys conducted regularly on behalf of the European Commission and other EU Institutions since 1973 ... The Standard Eurobarometer survey series is a cross-national longitudinal study, designed to compare and gauge trends within Member States of the European Union."	Nationally representative

Table S8: (*continued*) List and description of included survey series.

Source	Link to original data	Brief description	Target sample
European Values Study	bit.ly/3lRIBdc	"The European Values Study (EVS) is a large-scale, cross-national, repeated cross-sectional survey research programme on basic human values. It provides insights into the ideas, beliefs, preferences, attitudes, values and opinions of citizens all over Europe."	Nationally representative
Friedrich-Ebert-Stiftung Youth Studies in East Europe and Central Asia	bit.ly/3LZunlb	"These comprehensive studies capture youth attitudes, beliefs, and participation in core domains of life, including democracy, politics, governance, and the EU and also include sections devoted to education, employment, religion, family and lifestyles. Each nation used a questionnaire developed in 2005 for Germany's Shell Youth Studies as a template and made modifications based on their country's context to ensure relevance to particular intra-national issues."	Nationally representative of youths aged approximately 15 to 27 (there are small variations in ages sampled across countries)
Friedrich-Ebert-Stiftung Youth Studies in Southeast Europe	bit.ly/3NJfcxY	"These comprehensive studies capture youth attitudes, beliefs, and participation in core domains of life, including democracy, politics, governance, and the EU and also include sections devoted to education, employment, religion, family and lifestyles. Each nation used a questionnaire developed in 2005 for Germany's Shell Youth Studies as a template and made modifications based on their country's context to ensure relevance to particular intra-national issues."	Nationally representative of youths aged approximately 15 to 27 (there are small variations in ages sampled across countries)
International Social Survey Programme	bit.ly/38P0CGl; bit.ly/3GtwkVS; bit.ly/3PQFsbu; bit.ly/3MXf25P	"The International Social Survey Programme (ISSP) is a collaboration between different nations conducting surveys covering topics which are useful for social science research ... The results of the surveys provide a cross-national and cross-cultural perspective to individual national studies. By 2021, 58 countries have already taken part in the ISSP."	Nationally representative
LatinoBarometero	bit.ly/3a5jW24	"Latinobarometro is an annual public opinion survey that involves some 20,000 interviews in 18 Latin American countries, representing more than 600 million inhabitants ... [It] researches the development of democracy and economies as well as societies, using indicators of opinion, attitudes, behaviour and values."	Nationally representative
Life in Transition Survey	bit.ly/3PLGeX3; bit.ly/3NEyzbh	"The European Bank for Reconstruction and Development ... in collaboration with the World Bank, has carried out three such surveys [on 'how transition has affected the lives of people in regions, and what their views are on issues such as democracy, the role of the state, and prospects for the future']: one in 2006, one in 2010 and one in 2016. The most recent polled 51,000 households in 34 countries, mainly 'transition countries' in central and eastern Europe as well as Turkey and also, for the sake of comparison with more prosperous western neighbours, from Germany and Italy. For the first time, the survey also covered Cyprus and Greece."	Nationally representative
Perception and Acceptance of Religious Diversity among the European Population	bit.ly/3lWa5hX	"The Perception and Acceptance of Religious Diversity survey is one of the largest representative surveys to date on religious plurality in Europe. It captures the range of attitudes toward Islam and other religions among residents in five European countries. The survey was carried out in 2010 by the Cluster of Excellence 'Religion and Politics' of the University of Münster, together with TNS Emnid."	Nationally representative
Pew Global Attitudes Project	pewrsr.ch/3wToJeP	"The Pew Global Attitudes Project is a series of worldwide public opinion surveys encompassing a broad array of subjects ranging from people's assessments of their own lives to their views about the current state of the world and important issues of the day."	Nationally representative
Pew Religious Belief and National Belonging in Central and Eastern Europe	pewrsr.ch/3NFXHyj	This survey is part of the "Pew-Templeton Global Religious Future project, which analyzes religious change and its impact on societies around the world ... The survey in Central and Eastern Europe was conducted via face-to-face interviews under the direction of three research partners ... The survey is based on samples of noninstitutionalized adults ages 18 and older."	Nationally representative

Table S8: (*continued*) List and description of included survey series.

Source	Link to original data	Brief description	Target sample
Pew: Islam and Christianity in Sub-Saharan Africa	pewrsr.ch/2kQml7z	"The report was funded by generous grants from The Pew Charitable Trusts and the John Templeton Foundation as part of the Pew-Templeton Global Religious Futures Project, which aims to increase people's knowledge of religion around the world ... Our survey asked people to describe their religious beliefs and practices. We sought to gauge their knowledge of, and attitudes toward, other faiths.	Nationally representative
Pew: The World's Muslims—Religion, Politics and Society	pewrsr.ch/3LXoZiu	"This report examines the social and political views of Muslims around the world. It is based on public opinion surveys conducted by the Pew Research Center between 2008 and 2012 in a total of 39 countries and territories on three continents: Africa, Asia and Europe."	Muslims only
Pulse of Europe	bit.ly/3GssqwD	"The end of communism in the East and the immanent economic integration in the West have unleashed forces of nationalism and ethnicity contained for the last 45 years by the exigencies of the Cold War ... The Times Mirror Survey suggests that contradictory forces are pulling Europe in opposite directions ... Our in depth survey of 13,000 European in nine nations and the Republics of Russia, the Ukraine, and Lithuania finds many reasons to be pessimistic about future developments."	Nationally representative
Second European Union Minorities and Discrimination Survey	bit.ly/3t5LWcN	The European Union Agency for Fundamental Rights's "second European Union Minorities and Discrimination Survey (EU-MIDIS II) ... collected information from over 25,500 respondents with different ethnic minority and immigrant backgrounds across all 28 EU Member States."	Representative of immigrant groups and Roma populations
The Youth, Emotional Energy, and Political Violence: The Cases of Egypt and Saudi Arabia	bit.ly/38O5jjL	"This project intends to explore and explain the values and sociopolitical and cultural attitudes of young Egyptians and Saudis by looking at four general areas of values ... To analyze these four areas researchers conducted face-to-face interviews with 18 to 25 year old youths from Egypt (in the cities of Cairo, Alexandria, and El-Minya) and from Saudi Arabia (in the cities of Jeddah, Riyadh, and Dammam/Khobar). The research was supported by the United States Institute of Peace, the Mellon Foundation, and Eastern Michigan University."	Representative of youths aged 18 to 25 in certain cities
World Values Survey	bit.ly/3GuX5sZ	"A global research project that explores people's values and beliefs, how they change over time, and what social and political impact they have. Since 1981 a worldwide network of social scientists have conducted representative national surveys as part of WVS in almost 100 countries."	Nationally representative

Table S9: List of surveys considered but not included, with reason for exclusion. ♠ indicates that no questions on attitudes toward religious outgroups were asked; ♣ indicates that digitized microdata are not freely available online; ◇ indicates that the surveys were fielded prior to 1980; ♡ indicates duplicates of others survey datasets.

Source	Reason	Link
Americas Barometer/LAPOP	♠	bit.ly/3fPLUC4
Arab Public Opinion: A Survey in Six Countries	♣	bit.ly/3rBzctd
Asia Europe Survey: A Multinational Comparative Study in 18 Countries	♠	bit.ly/3e8CQrx
Asian Barometer Survey (ABS) [see: EABS]	♠	bit.ly/3Vcbe5d
Attitudes towards Europe	◇	bit.ly/3MbEV2d
Central and Eastern Eurobarometer	♠	bit.ly/3yknWVG
Central Asia Barometer	♠	bit.ly/3ylDXuA
Civicus Civil Society Index	♣	bit.ly/3SGaELC
East Asia Value Survey	♣	bit.ly/3EnrcDy
East Asian Social Survey (EASS)	♠	bit.ly/3Ce57ou
EU Neighborhood Barometer	♠	bit.ly/3M9I4jj
EUCROSS (The Europeanisation of Everyday Life: Cross-Border Practices and Transnational Identities among EU and Third-Country Citizens)	♠	bit.ly/3V6f1RI
European Election Studies	♡	bit.ly/3V0LVTE
European Quality of Life Survey	♠	bit.ly/2T8X5bT
European Social Survey	♠	bit.ly/3Vcf66f
Gallup World Poll	♣	bit.ly/3SGaZ0T
Gender And Adolescence: Global Evidence	♠ (asked only in Jordan, therefore not multi-national)	bit.ly/3CDDt5B
Generations and Gender Survey	♠	bit.ly/3ejs1Tg
Group-Focused Enmity in Europe. A Representative Cross-European Survey on Group-Focused Enmity and Political Attitudes	♣	bit.ly/3VcfHVx
Images of the World in the Year 2000	♣	bit.ly/3Eo8rQK
International Civic and Citizenship Education Study	♠	bit.ly/3V6Oeoa
INTUNE—Integrated and United? A Quest for Citizenship in an Ever Closer Europe	♠	bit.ly/3SG3qag
Memory, Youth, Political Legacy And Civic Engagement	♣	bit.ly/3ebTWoh
Multiple Indicator Cluster Survey	♠	bit.ly/3RJt4K1
New Baltic Barometer	♠	bit.ly/3Ce8lZ8
New Departures: Religion and Attitudes toward Church in Eastern (Central) Europe	♣	bit.ly/3EoJEfc (link to original survey cannot be found)
New Europe Barometer	♠	bit.ly/3T0C12x
New Soviet Citizen Surveys	♠	bit.ly/3V1PQ2J
Pew: Religion in Latin America	♠	pewrsr.ch/3MdoAdF
Reader's Digest EURODATA—The Reader's Digest Survey of Europe Today	♠	bit.ly/3rC050g
Religious Fundamentalism and Radicalization Survey	♠	bit.ly/3RBzwCV
Social Change in the Baltic Countries	♠	bit.ly/3SUxXBa
The Voice of the People	♠	bit.ly/3V1QGMV
Transatlantic Trend Survey	♠	bit.ly/3CCF8IE
Young Lives Survey	♠	bit.ly/3edvq6g

Table S10: Ordinary least-squares regression estimates of the relationship between survey coverage and whether or not a national election involved civilian deaths. The unit of analysis is the national election. In the table header, "m" refers to months. Included in the sample are all national elections from 1982, which is the year of the first survey in this study, to 2020, the end of the NELDA dataset. Standard errors clustered by country are in parentheses.

	Any survey responses within...			Number of survey responses within...		
	3m	6m	9m	3m	6m	9m
	...before or after election					
	(1)	(2)	(3)	(4)	(5)	(6)
Election involved civilian deaths	0.012	0.000	-0.021	59.841	73.378	-27.244
	(0.024)	(0.030)	(0.035)	(92.728)	(181.764)	(227.969)
N	1,660	1,660	1,660	1,660	1,660	1,660
R^2	0.19	0.21	0.22	0.14	0.21	0.22
Country FE	Y	Y	Y	Y	Y	Y

*p<0.1; **p<0.05; ***p<0.01

Table S11: List of national elections employed in computing treatment indicators for the analysis of attitudes regarding religious intolerance. Note, this is not necessarily a complete list of national elections in each included country; an election's appearance depends on the timing of surveys fielded in a given country.

Country	National election dates employed in analyses of religious intolerance
Albania	Jul 06, 1997; Jun 24, 2001; Jul 08, 2001; Jul 03, 2005; Jun 28, 2009; Jun 23, 2013; Jun 25, 2017
Algeria	Apr 15, 1999; May 30, 2002; Apr 09, 2009; May 10, 2012; Apr 17, 2014; May 04, 2017; Dec 12, 2019
Andorra	Apr 24, 2005; Apr 26, 2009; Mar 01, 2015; Apr 07, 2019
Argentina	May 14, 1989; Aug 11, 1991; May 14, 1995; Oct 26, 1997; Oct 24, 1999; Oct 14, 2001; Oct 23, 2005; Oct 28, 2007; Jun 28, 2009; Oct 23, 2011; Oct 25, 2015; Nov 22, 2015; Oct 29, 2017
Armenia	Feb 19, 2008; May 06, 2012; Feb 18, 2013; Apr 02, 2017; Dec 09, 2018
Australia	Oct 09, 2004; Nov 24, 2007; Aug 21, 2010; Sep 07, 2013; Jul 02, 2016; May 18, 2019
Austria	Dec 17, 1995; Apr 19, 1998; Oct 01, 2006; Sep 28, 2008; Apr 25, 2010; Sep 29, 2013; Apr 24, 2016; Oct 15, 2017; Sep 29, 2019
Azerbaijan	Nov 26, 1995; Oct 11, 1998; Oct 15, 2008; Nov 07, 2010; Oct 09, 2013; Nov 01, 2015; Apr 11, 2018
Bahrain	Dec 02, 2006; Oct 23, 2010
Bangladesh	Jun 12, 1996; Oct 01, 2001; Dec 29, 2008; Jan 05, 2015; Dec 30, 2018
Belarus	Dec 10, 1995; Oct 15, 2000; Oct 29, 2000; Sep 09, 2001; Sep 28, 2008; Dec 19, 2010; Sep 23, 2012; Oct 11, 2015; Sep 11, 2016
Belgium	Dec 13, 1987; Nov 24, 1991; May 21, 1995; Jun 13, 1999; Jun 10, 2007; Jun 13, 2010; May 25, 2014; May 26, 2019
Benin	Apr 30, 2011; Apr 26, 2015; Mar 20, 2016; Apr 28, 2019
Bolivia	Jun 01, 1997; Jun 30, 2002; Oct 12, 2014; Oct 20, 2019
Bosnia	Sep 14, 1996; Sep 12, 1998; Nov 11, 2000; Oct 05, 2002; Oct 01, 2006; Oct 03, 2010; Oct 12, 2014; Oct 07, 2018
Botswana	Oct 30, 2004; Oct 16, 2009; Oct 24, 2014; Oct 23, 2019
Brazil	Oct 03, 1990; Oct 03, 1994; Oct 04, 1998; Oct 06, 2002; Oct 29, 2006; Oct 03, 2010; Oct 31, 2010; Oct 05, 2014; Oct 26, 2014; Oct 07, 2018
Bulgaria	Jun 17, 1990; Oct 13, 1991; Apr 19, 1997; Jun 17, 2001; Nov 18, 2001; Jun 25, 2005; Oct 29, 2006; Jul 05, 2009; Oct 23, 2011; May 12, 2013; Oct 05, 2014; Nov 06, 2016; Mar 26, 2017
Burkina Faso	Nov 13, 2005; May 06, 2007; Dec 02, 2012; Nov 29, 2015; Nov 22, 2020
Burundi	Jul 23, 2010; Jun 29, 2015
Côte d'Ivoire	Dec 11, 2011; Oct 25, 2015; Dec 18, 2016; Oct 31, 2020
Cambodia	Jul 29, 2018
Cameroon	Jul 22, 2007; Oct 09, 2011; Sep 30, 2013; Oct 07, 2018
Canada	Nov 21, 1988; Oct 25, 1993; Jun 02, 1997; Nov 27, 2000; Jun 28, 2004; Jan 23, 2006; Oct 14, 2008; May 02, 2011; Oct 21, 2019
Cape Verde	Aug 21, 2011; Mar 20, 2016; Oct 02, 2016
Chad	May 03, 2006; Feb 13, 2011
Chile	Dec 14, 1989; Dec 11, 1993; Dec 11, 1997; Dec 12, 1999; Jan 16, 2000; Dec 16, 2001; Jan 15, 2006; Dec 13, 2009; Jan 17, 2010; Nov 17, 2013; Dec 17, 2017
Colombia	Jun 21, 1998; Mar 10, 2002; May 26, 2002; Mar 12, 2006; Jun 20, 2010; Mar 09, 2014; Jun 17, 2018
Congo - Kinshasa	Oct 29, 2006; Nov 28, 2011
Costa Rica	Feb 01, 1998; Feb 03, 2002
Croatia	Nov 05, 1995; Apr 13, 1997; Jan 03, 2000; Jan 24, 2000; Feb 07, 2000; Nov 25, 2007; Dec 27, 2009; Jan 10, 2010; Dec 04, 2011; Nov 08, 2015; Sep 11, 2016; Dec 22, 2019
Cyprus	May 27, 2001; Feb 16, 2003; May 21, 2006; Feb 17, 2008; Feb 24, 2008; May 22, 2011; Feb 17, 2013; Feb 24, 2013; May 22, 2016; Feb 04, 2018
Czechia	May 31, 1996; Jun 14, 2002; Oct 25, 2002; Oct 27, 2006; Oct 17, 2008; Oct 24, 2008; May 28, 2010; Oct 22, 2010; Oct 12, 2012; Oct 17, 2014; Oct 20, 2017; Jan 12, 2018; Jan 26, 2018; Oct 05, 2018; Oct 12, 2018; Oct 02, 2020
Czechoslovakia	Jun 08, 1990; Jun 05, 1992
Denmark	May 10, 1988; Dec 12, 1990; Sep 21, 1994; Mar 11, 1998; Nov 13, 2007; Sep 15, 2011; Jun 18, 2015; Jun 05, 2019
Djibouti	Feb 08, 2008; Apr 08, 2011
Dominican Republic	May 16, 2008; May 16, 2010

Table S11: (*continued*) List of national elections employed.

Country	National election dates employed in analyses of religious intolerance
Ecuador	Jul 12, 1998; Oct 20, 2002; Feb 17, 2013; Feb 19, 2017; Apr 02, 2017
Egypt	Sep 26, 1999; Oct 18, 2000; Nov 08, 2000; Sep 07, 2005; Nov 09, 2005; Jun 11, 2007; Jun 01, 2010; Nov 28, 2010; Nov 28, 2011; Dec 05, 2011; Oct 18, 2015; Mar 26, 2018; Aug 11, 2020
El Salvador	Mar 16, 1997; Mar 07, 1999; Mar 12, 2000
Estonia	Mar 05, 1995; Mar 07, 1999; Mar 02, 2003; Mar 04, 2007; Mar 06, 2011; Mar 01, 2015; Mar 03, 2019
Eswatini	Sep 20, 2013; Sep 21, 2018
Ethiopia	May 15, 2005; May 23, 2010; May 24, 2015
Finland	Mar 19, 1995; Mar 21, 1999; Mar 16, 2003; Jan 15, 2006; Mar 18, 2007; Apr 17, 2011; Apr 19, 2015; Jan 28, 2018; Apr 14, 2019
France	Jun 12, 1988; Mar 21, 1993; Mar 28, 1993; Apr 23, 1995; May 07, 1995; May 25, 1997; Jun 01, 1997; Jun 16, 2002; Apr 22, 2007; Jun 17, 2007; Apr 22, 2012; Jun 17, 2012; Apr 23, 2017; Jun 18, 2017
Gabon	Dec 17, 2011; Aug 27, 2016; Oct 06, 2018
Gambia	Apr 06, 2017
Georgia	Nov 19, 1995; Oct 31, 1999; May 21, 2008; Oct 01, 2012; Oct 27, 2013; Oct 08, 2016; Oct 30, 2016; Oct 28, 2018; Nov 28, 2018; Oct 31, 2020
Germany	Jan 25, 1987; Dec 02, 1990; Oct 16, 1994; Sep 27, 1998; Sep 22, 2002; Sep 18, 2005; Sep 27, 2009; Sep 22, 2013; Sep 24, 2017
Ghana	Dec 07, 2004; Dec 07, 2008; Dec 28, 2008; Dec 07, 2012; Dec 07, 2016; Dec 07, 2020
Greece	Jun 02, 1985; Jun 18, 1989; Apr 08, 1990; Oct 10, 1993; Sep 22, 1996; Apr 09, 2000; Sep 16, 2007; Oct 04, 2009; Jun 17, 2012; Jan 25, 2015; Sep 20, 2015; Jul 07, 2019
Guatemala	Jan 07, 1996; Nov 07, 1999; Dec 28, 2003; Sep 09, 2007; Aug 11, 2019
Guinea	Sep 28, 2013; Oct 11, 2015; Mar 22, 2020
Guinea-Bissau	Nov 16, 2008; Jun 28, 2009
Haiti	Oct 25, 2015; Nov 20, 2016
Honduras	Nov 30, 1997; Nov 25, 2001
Hungary	Apr 08, 1990; May 08, 1994; Apr 21, 2002; Apr 09, 2006; Apr 23, 2006; Apr 11, 2010; Apr 25, 2010; Apr 06, 2014; Apr 08, 2018
Iceland	Oct 29, 2016; Oct 28, 2017; Jun 27, 2020
India	Nov 22, 1989; May 20, 1991; Apr 27, 1996; Sep 05, 1999; Apr 20, 2004; Apr 16, 2009; Apr 07, 2014
Indonesia	Jun 07, 1999; Apr 05, 2004; Sep 20, 2004; Apr 09, 2009; Jul 08, 2009; Apr 09, 2014; Jul 09, 2014; Apr 17, 2019
Iran	Oct 23, 1998; Feb 18, 2000; Jun 24, 2005; Mar 14, 2008; Mar 02, 2012; May 04, 2012; May 19, 2017
Iraq	Mar 27, 2000; Jan 30, 2005; Dec 15, 2005; Mar 07, 2010; Apr 30, 2014; May 12, 2018
Ireland	Feb 17, 1987; Jun 15, 1989; Nov 09, 1990; Nov 25, 1992; Jun 06, 1997; May 24, 2007; Feb 25, 2011; Oct 27, 2011; Feb 26, 2016; Oct 26, 2018; Feb 08, 2020
Israel	Feb 10, 2009; Mar 17, 2015; Apr 09, 2019
Italy	Jun 14, 1987; Apr 05, 1992; Mar 27, 1994; Apr 21, 1996; May 13, 2001; Apr 09, 2006; Apr 13, 2008; Feb 24, 2013; Mar 04, 2018
Japan	Feb 18, 1990; Jul 26, 1992; Sep 11, 2005; Jul 29, 2007; Aug 30, 2009; Jul 11, 2010; Dec 16, 2012; Oct 22, 2017; Jul 21, 2019
Jordan	Nov 04, 1997; Jun 17, 2003; Nov 20, 2007; Nov 09, 2010; Jan 23, 2013; Sep 20, 2016; Nov 10, 2020
Kazakhstan	Aug 18, 2007; Apr 03, 2011; Jan 15, 2012; Apr 26, 2015; Mar 20, 2016; Jun 09, 2019
Kenya	Dec 27, 2007; Mar 04, 2013; Aug 08, 2017; Oct 26, 2017
Kosovo	Dec 12, 2010; Jun 08, 2014; Jun 11, 2017; Oct 06, 2019
Kuwait	Nov 26, 2016; Dec 05, 2020
Kyrgyzstan	Dec 24, 1995; Feb 20, 2000; Oct 29, 2000; Feb 27, 2005; Jul 23, 2009; Oct 10, 2010; Oct 30, 2011; Oct 04, 2015; Oct 15, 2017; Oct 04, 2020
Latvia	Sep 30, 1995; Oct 03, 1998; Oct 05, 2002; Oct 07, 2006; Oct 02, 2010; Sep 17, 2011; Oct 04, 2014; Oct 06, 2018
Lebanon	Aug 27, 2000; May 28, 2005; Jun 07, 2009; May 06, 2018
Lesotho	May 26, 2012
Liberia	Nov 08, 2005; Oct 11, 2011; Dec 20, 2014; Oct 10, 2017; Dec 26, 2017; Dec 08, 2020
Libya	Jul 07, 2012; Feb 20, 2014; Jun 25, 2014
Lithuania	Oct 25, 1992; Oct 08, 2000; Dec 22, 2002; Oct 24, 2004; Oct 12, 2008; May 17, 2009; Oct 14, 2012; May 25, 2014; Oct 09, 2016; Oct 23, 2016; May 12, 2019; May 26, 2019; Oct 11, 2020

Table S11: (*continued*) List of national elections employed.

Country	National election dates employed in analyses of religious intolerance
Luxembourg	Jun 17, 1984; Jun 18, 1989; Jun 12, 1994; Jun 13, 1999; Jun 13, 2004; Jun 07, 2009; Oct 20, 2013; Oct 14, 2018
Madagascar	Dec 20, 2013; Nov 07, 2018
Malawi	May 19, 2009; May 20, 2014; May 21, 2019
Malaysia	Mar 21, 2004; Mar 08, 2008; May 05, 2013; May 09, 2018
Mali	Jul 28, 2002; Apr 29, 2007; Jul 22, 2007; Jul 28, 2013; Nov 24, 2013; Jul 29, 2018
Malta	Sep 05, 1998; Apr 12, 2003; Mar 08, 2008; Mar 09, 2013; Jun 03, 2017
Mauritania	Jun 22, 2019
Mauritius	May 05, 2010; Dec 10, 2014; Nov 07, 2019
Mexico	Jul 06, 1988; Aug 18, 1991; Aug 21, 1994; Jul 06, 1997; Jul 02, 2000; Jul 06, 2003; Jul 02, 2006; Jul 05, 2009; Jul 01, 2012; Jun 07, 2015; Jul 01, 2018
Moldova	Dec 01, 1996; Mar 22, 1998; Feb 25, 2001; Mar 06, 2005; Apr 05, 2009; Jul 29, 2009; Nov 28, 2010; Nov 30, 2014; Oct 30, 2016
Mongolia	May 24, 2009; Jun 28, 2012; Jun 26, 2013; Jun 29, 2016; Jul 09, 2017; Jun 24, 2020
Montenegro	Apr 06, 2008; Mar 29, 2009; Oct 14, 2012; Apr 07, 2013; Oct 16, 2016; Apr 15, 2018
Morocco	Sep 27, 2002; Sep 07, 2007; Nov 25, 2011; Oct 07, 2016
Mozambique	Dec 01, 2004; Oct 28, 2009; Oct 15, 2014; Oct 15, 2019
Myanmar	Nov 08, 2015; Nov 08, 2020
Namibia	Nov 27, 2009; Nov 28, 2014; Nov 27, 2019
Nepal	Dec 07, 2017
Netherlands	May 21, 1986; Sep 06, 1989; May 03, 1994; May 06, 1998; Jan 22, 2003; Nov 22, 2006; Jun 09, 2010; Sep 12, 2012; Mar 15, 2017
New Zealand	Sep 17, 2005; Nov 08, 2008; Nov 26, 2011; Sep 20, 2014; Sep 23, 2017; Oct 17, 2020
Nicaragua	Oct 20, 1996; Nov 04, 2001; Nov 06, 2016
Niger	Mar 12, 2011; Feb 21, 2016; Mar 20, 2016; Dec 27, 2020
Nigeria	Aug 27, 1983; Jul 04, 1992; Jun 12, 1993; Apr 25, 1998; Feb 27, 1999; Apr 12, 2003; Apr 19, 2003; Apr 21, 2007; Apr 09, 2011; Apr 16, 2011; Mar 28, 2015; Feb 23, 2019
North Macedonia	Nov 14, 1999; Sep 15, 2002; Apr 05, 2009; Jun 05, 2011; Apr 27, 2014; Dec 11, 2016; Apr 21, 2019
Norway	Sep 10, 1989; Sep 12, 1993; Sep 15, 1997; Sep 12, 2005; Sep 14, 2009; Sep 11, 2017
Pakistan	Feb 03, 1997; Apr 30, 2002; Oct 10, 2002; Feb 18, 2008; May 11, 2013; Jul 25, 2018
Panama	May 08, 1994; May 02, 1999
Paraguay	May 10, 1998; Aug 13, 2000
Peru	Apr 09, 1995; Apr 09, 2000; Jun 03, 2001; Apr 09, 2006; Jun 04, 2006; Apr 10, 2011; Jun 05, 2011; Apr 10, 2016; Jun 05, 2016; Jan 26, 2020
Philippines	May 08, 1995; May 11, 1998; May 14, 2001; May 10, 2004; May 14, 2007; May 10, 2010; May 13, 2013; May 09, 2016; May 13, 2019
Poland	Dec 09, 1990; Oct 27, 1991; Sep 23, 2001; Sep 25, 2005; Oct 23, 2005; Oct 21, 2007; Jun 20, 2010; Jul 04, 2010; Oct 09, 2011; May 10, 2015; May 24, 2015; Oct 25, 2015; Oct 13, 2019
Portugal	Jul 19, 1987; Jan 13, 1991; Oct 06, 1991; Oct 01, 1995; Jan 14, 1996; Oct 10, 1999; Jan 22, 2006; Sep 27, 2009; Jan 23, 2011; Jun 05, 2011; Oct 04, 2015; Jan 24, 2016; Oct 06, 2019
Romania	Nov 17, 1996; Nov 26, 2000; Dec 10, 2000; Nov 28, 2004; Dec 12, 2004; May 19, 2007; Nov 30, 2008; Dec 06, 2009; Jul 29, 2012; Dec 09, 2012; Nov 02, 2014; Nov 16, 2014; Dec 11, 2016; Nov 10, 2019
Russia	Mar 04, 1979; Mar 04, 1984; Mar 04, 1990; Jun 12, 1991; Dec 17, 1995; Jun 16, 1996; Dec 19, 1999; Mar 26, 2000; Dec 07, 2003; Mar 14, 2004; Dec 02, 2007; Mar 02, 2008; Dec 04, 2011; Mar 04, 2012; Sep 18, 2016; Mar 18, 2018
Rwanda	Sep 29, 2003; Sep 15, 2008; Aug 09, 2010; Sep 16, 2013
São Tomé & Príncipe	Oct 12, 2014; Jul 17, 2016; Aug 07, 2016; Oct 07, 2018
Senegal	Jun 03, 2007; Feb 26, 2012; Jul 01, 2012; Jul 02, 2017; Feb 24, 2019
Serbia	Jan 21, 2007; May 11, 2008; May 06, 2012; Mar 06, 2014; Apr 24, 2016; Apr 02, 2017; Jun 21, 2020
Sierra Leone	Nov 17, 2012; Mar 07, 2018; Mar 31, 2018
Singapore	Aug 27, 2011; Sep 11, 2015; Jul 10, 2020

Table S11: (*continued*) List of national elections employed.

Country	National election dates employed in analyses of religious intolerance
Slovakia	Sep 30, 1994; Sep 20, 2002; Apr 03, 2004; Jun 17, 2006; Mar 21, 2009; Apr 04, 2009; Jun 12, 2010; Mar 10, 2012; Mar 29, 2014; Mar 05, 2016; Mar 16, 2019; Mar 30, 2019; Feb 29, 2020
Slovenia	Dec 06, 1992; Nov 10, 1996; Nov 23, 1997; Oct 15, 2000; Nov 10, 2002; Oct 03, 2004; Oct 21, 2007; Nov 11, 2007; Sep 21, 2008; Dec 04, 2011; Dec 02, 2012; Jul 13, 2014; Oct 22, 2017; Nov 12, 2017; Jun 03, 2018
South Africa	Jun 02, 1999; Apr 14, 2004; Apr 22, 2009; May 07, 2014; May 08, 2019
South Korea	Mar 25, 1981; Feb 12, 1985; Apr 26, 1988; Mar 24, 1992; Apr 13, 2000; Dec 19, 2002; Apr 15, 2004; Dec 19, 2007; Apr 09, 2008; Apr 11, 2012; May 09, 2017; Apr 15, 2020
Spain	Jun 22, 1986; Oct 29, 1989; Jun 06, 1993; Mar 03, 1996; Mar 12, 2000; Mar 14, 2004; Mar 09, 2008; Nov 20, 2011; Dec 20, 2015; Jun 26, 2016; Apr 28, 2019; Nov 10, 2019
Sri Lanka	Nov 17, 2005; Jan 26, 2010; Jan 08, 2015; Nov 16, 2019
Sudan	Apr 11, 2010; Apr 13, 2015
Suriname	May 25, 2015; May 25, 2020
Sweden	Sep 18, 1988; Sep 15, 1991; Sep 18, 1994; Sep 20, 1998; Sep 15, 2002; Sep 17, 2006; Sep 19, 2010; Sep 14, 2014; Sep 09, 2018
Switzerland	Oct 18, 1987; Oct 20, 1991; Oct 22, 1995; Oct 24, 1999; Oct 19, 2003; Oct 21, 2007; Oct 23, 2011; Oct 18, 2015; Oct 20, 2019
Taiwan	May 14, 2005; Jan 12, 2008; Mar 22, 2008; Jan 14, 2012; Jan 16, 2016; Jan 11, 2020
Tajikistan	Feb 28, 2010; Nov 06, 2013; Mar 01, 2015; Mar 01, 2020
Tanzania	Nov 05, 2000; Dec 14, 2005; Oct 31, 2010; Oct 25, 2015; Oct 28, 2020
Thailand	Apr 19, 2006; Dec 23, 2007; Jul 03, 2011; Feb 02, 2014; Mar 24, 2019
Togo	Jul 25, 2013; Apr 25, 2015; Dec 20, 2018
Trinidad & Tobago	Oct 07, 2002; Nov 05, 2007; May 24, 2010; Sep 07, 2015
Tunisia	Oct 25, 2009; Oct 23, 2011; Oct 26, 2014; Dec 21, 2014; Sep 15, 2019; Oct 13, 2019
Turkey	Nov 29, 1987; Oct 20, 1991; Dec 24, 1995; Apr 18, 1999; Nov 03, 2002; Jul 22, 2007; Jun 12, 2011; Aug 10, 2014; Nov 01, 2015; Jun 24, 2018
Uganda	Jun 27, 1996; Mar 12, 2001; Feb 23, 2006; Feb 18, 2011; Feb 18, 2016
UK	Jun 11, 1987; Apr 09, 1992; May 01, 1997; Jun 07, 2001; May 05, 2005; May 06, 2010; May 07, 2015; Jun 08, 2017; Dec 12, 2019
Ukraine	Dec 01, 1991; Jul 24, 1994; Mar 29, 1998; Mar 26, 2006; Sep 30, 2007; Jan 17, 2010; Feb 07, 2010; Oct 28, 2012; May 25, 2014; Oct 26, 2014; Mar 31, 2019; Apr 21, 2019; Jul 21, 2019
Uruguay	Nov 27, 1994; Oct 31, 1999; Oct 31, 2004; Oct 25, 2009; Nov 29, 2009; Oct 26, 2014
US	Nov 08, 1988; Nov 06, 1990; Nov 08, 1994; Nov 05, 1996; Nov 03, 1998; Nov 07, 2000; Nov 05, 2002; Nov 02, 2004; Nov 07, 2006; Nov 04, 2008; Nov 02, 2010; Nov 06, 2012; Nov 08, 2016; Nov 06, 2018; Nov 03, 2020
Uzbekistan	Dec 27, 2009; Dec 21, 2014; Mar 29, 2015; Dec 04, 2016
Venezuela	Dec 05, 1993; Nov 08, 1998; Dec 06, 1998; Jul 25, 1999; Jul 30, 2000; Aug 15, 2004; Dec 03, 2006; Sep 26, 2010; Dec 06, 2020
Vietnam	Jul 20, 1997; May 19, 2002; May 20, 2007; May 22, 2016
Zambia	Sep 28, 2006; Oct 30, 2008; Sep 20, 2011; Jan 20, 2015; Aug 11, 2016
Zimbabwe	Jun 24, 2000; Mar 09, 2002; Jun 27, 2008; Jul 31, 2013; Jul 30, 2018

Table S12: List of unique question wordings and response-options. Dichotomous recodes for the outcome measure of religious intolerance are indicated by the square brackets in the third column.

Source(s)	Question text	Response options [recodes]	Question-type
A Cross-National Survey of Muslim Attitudes Wave 1; A Cross-National Survey of Muslim Attitudes Wave 2	Do you personally believe it is acceptable or not acceptable for a Muslim man to marry a non-Muslim woman who does not convert to Islam?	1 = Yes, it is acceptable for a Muslim man to marry a non-Muslim woman [=0]; 2 = No, it is not acceptable for a Muslim man to marry a non-Muslim woman [=1]; 3 = In certain situations it may be acceptable [=0]	Role: family
A Cross-National Survey of Muslim Attitudes Wave 1; A Cross-National Survey of Muslim Attitudes Wave 2	Do you personally believe it is acceptable or not acceptable for a Muslim woman to marry a non-Muslim man who does not convert to Islam?	1 = Yes, it is acceptable for a Muslim woman to marry a non-Muslim man [=0]; 2 = No, it is not acceptable for a Muslim woman to marry a non-Muslim man [=1]; 3 = In certain situations it may be acceptable [=0]	Role: family
Afrobarometer Round 6	For each of the following types of people, please tell me whether you would like having people from this group as neighbors, dislike it, or not care: People of different religion.	1 = Strongly dislike [=1]; 2 = Somewhat dislike [=1]; 3 = Would not care [=0]; 4 = Somewhat like [=0]; 5 = Strongly like [=0]	Role: neighbor
Afrobarometer Round 7	For each of the following types of people, please tell me whether you would like having people from this group as neighbors, dislike it, or not care: People of different religion.	1 = Strongly dislike [=1]; 2 = Somewhat dislike [=1]; 3 = Would not care [=0]; 4 = Somewhat like [=0]; 5 = Strongly like [=0]	Role: neighbor
Arab Barometer Wave 1	Which of the following groups do you wish to have as neighbors? Followers of other religions.	1 = I do not wish [=1]; 2 = I do not mind [=0]	Role: neighbor
Arab Barometer Wave 2	Members of which of the following groups would you not like to have as neighbors? Followers of other religions.	1 = I do not want them to be my neighbors [=1]; 2 = I do not object [=0]	Role: neighbor
Arab Barometer Wave 2	Members of which of the following groups would you not like to have as neighbors? Those who belong to religious sects other than yours.	1 = I do not want them to be my neighbors [=1]; 2 = I do not object [=0]	Role: neighbor
Arab Barometer Wave 2	To what extent do you consider the following factors obstacles to accepting your son/daughter/sister/brother's marriage? From a different religion or denomination.	1 = Constitutes an obstacle to a great extent [=1]; 2 = Constitutes an obstacle to a medium extent [=1]; 3 = Constitutes an obstacle to a limited extent [=0]; 4 = Does not constitute an obstacle whatsoever [=0]	Role: family
Arab Barometer Wave 4	For each of the following types of people, please tell me whether you would like having people from this group as neighbors, dislike it, or not care? People of a different sect of Islam.	1 = Strongly dislike [=1]; 2 = Somewhat dislike [=1]; 3 = Would not care [=0]; 4 = Somewhat like [=0]; 5 = Strongly like [=0]	Role: neighbor
Arab Barometer Wave 4	For each of the following types of people, please tell me whether you would like having people from this group as neighbors, dislike it, or not care? People of a different religion.	1 = Strongly dislike [=1]; 2 = Somewhat dislike [=1]; 3 = Would not care [=0]; 4 = Somewhat like [=0]; 5 = Strongly like [=0]	Role: neighbor
Arab Barometer Wave 5	For each of the following types of people, please tell me how much you would like having people from this group as your neighbors. People of a different Christian denomination. [Asked of Christians only.]	1 = Strongly dislike [=1]; 2 = Dislike [=1]; 3 = Neither dislike, nor like [=0]; 4 = Like [=0]; 5 = Strongly like [=0]	Role: neighbor
Arab Barometer Wave 5	For each of the following types of people, please tell me how much you would like having people from this group as your neighbors. People of a different religion.	1 = Strongly dislike [=1]; 2 = Dislike [=1]; 3 = Neither dislike, nor like [=0]; 4 = Like [=0]; 5 = Strongly like [=0]	Role: neighbor
Arab Barometer Wave 5	For each of the following types of people, please tell me how much you would like having people from this group as your neighbors. People of a different sect of Islam. [Asked of Muslims only.]	1 = Strongly dislike [=1]; 2 = Dislike [=1]; 3 = Neither dislike, nor like [=0]; 4 = Like [=0]; 5 = Strongly like [=0]	Role: neighbor

Table S12: (*continued*) List of unique question wordings and response-options.

Source(s)	Question text	Response options [recodes]	Question-type
Arab Barometer Wave 6	For each of the following types of people, please tell me whether you would like having people from this group as neighbors, dislike it, or not care. People of a different Christian denomination. [Asked of Christians only.]	1 = Strongly dislike [=1]; 2 = Somewhat dislike [=1]; 3 = Would not care [=0]; 4 = Somewhat like [=0]; 5 = Strongly like [=0]	Role: neighbor
Arab Barometer Wave 6	For each of the following types of people, please tell me whether you would like having people from this group as neighbors, dislike it, or not care. People of a different religion.	1 = Strongly dislike [=1]; 2 = Somewhat dislike [=1]; 3 = Would not care [=0]; 4 = Somewhat like [=0]; 5 = Strongly like [=0]	Role: neighbor
Arab Barometer Wave 6	For each of the following types of people, please tell me whether you would like having people from this group as neighbors, dislike it, or not care. People of a different sect of Islam. [Asked of Muslims only.]	1 = Strongly dislike [=1]; 2 = Somewhat dislike [=1]; 3 = Would not care [=0]; 4 = Somewhat like [=0]; 5 = Strongly like [=0]	Role: neighbor
Arab Opinion Index Wave 7	Which of the following groups would you not like to have as neighbors: Followers of different religions.	1 = I do not want them as neighbors [=1]; 2 = I am impartial [=0]	Role: neighbor
Arab Opinion Index Wave 7	Would you accept or oppose your daughter or your sister marrying a person from another sect or confession?	1 = Accept [=0]; 2 = Oppose [=1]	Role: family
Candidate Eurobarometer 2002	Some people are disturbed by the opinions, customs and way of life of people different from themselves. And do you find the presence of people of another religion disturbing?	1 = Disurbing [=1]; 2 = Not disturbing [=0]	Discomfort
Caucasus Barometer 2009	Would you approve or disapprove of being friends with Jews?	1 = Approve [=0]; 0 = Disapprove [=1]	Role: friend
Caucasus Barometer 2019	Can you please tell me whether you approve or disapprove people like you doing business with a Jehovah's Witness?	1 = Yes (sic, means approve) [=0]; 0 = No (sic, means disapprove) [=1]	Role: work
Caucasus Barometer 2019	Can you please tell me whether you approve or disapprove people like you marrying a Jehovah's Witness?	1 = Yes (sic, means approve) [=0]; 0 = No (sic, means disapprove) [=1]	Role: family
Caucasus Barometer 2019; Caucasus Barometer 2017	Which of these people would you not wish to have as your neighbors most?	1 = People following a religion that is different to yours [=1]; 2 = People having politics views that are different to yours [=0]; 3 = Europeans who come to live in /country/ and want to stay [=0]; 4 = Asians who come to live in /country/ and want to stay [=0]; 5 = Drug addicts [=0]; 6 = Homosexuals [=0]; 7 = Black people [=0]; 8 = Criminals [=0]; 9 = Other, specify [=0]; 10/-5 = I would not wish any of these people as my neighbors [=1]	Role: neighbor
Caucasus Barometer 2019; Caucasus Barometer 2017; Caucasus Barometer 2015; Caucasus Barometer 2013	Can you please tell me whether you approve or disapprove of people of your ethnicity doing business with a Molokan.	1 = Approve [=0]; 0 = Disapprove [=1]	Role: work
Caucasus Barometer 2019; Caucasus Barometer 2017; Caucasus Barometer 2015; Caucasus Barometer 2013	Would you approve or disapprove of women of your ethnicity marrying a Molokan?	1 = Approve [=0]; 0 = Disapprove [=1]	Role: family
Caucasus Barometer 2019; Caucasus Barometer 2017; Caucasus Barometer 2015; Caucasus Barometer 2013; Caucasus Barometer 2012; Caucasus Barometer 2011; Caucasus Barometer 2010; Caucasus Barometer 2009	Now I am going to read out a list of nationalities. Can you please tell me whether you approve or disapprove of people of your ethnicity doing business with a Jew.	1 = Approve [=0]; 0 = Disapprove [=1]	Role: work

Table S12: (*continued*) List of unique question wordings and response-options.

Source(s)	Question text	Response options [recodes]	Question-type
Caucasus Barometer 2019; Caucasus Barometer 2017; Caucasus Barometer 2015; Caucasus Barometer 2013; Caucasus Barometer 2012; Caucasus Barometer 2011; Caucasus Barometer 2010; Caucasus Barometer 2009	Would you approve or disapprove of women of your ethnicity marrying a Jew?	1 = Approve [=0]; 0 = Disapprove [=1]	Role: family
Comparative Cross-National Study of Religious Fundamentalism, Developmental Idealism, Values, and Morality in the Middle East and North Africa	I am going to name a number of groups. For each one, could you tell me how much trust, in general, you have in them: is it a great deal of trust, some trust, not very much trust or none at all? Allawis.	1 = A great deal [=0]; 2 = Some [=0]; 3 = Not very much [=1]; 4 = None at all [=1]	Trust
Comparative Cross-National Study of Religious Fundamentalism, Developmental Idealism, Values, and Morality in the Middle East and North Africa	I am going to name a number of groups. For each one, could you tell me how much trust, in general, you have in them: is it a great deal of trust, some trust, not very much trust or none at all? Christians.	1 = A great deal [=0]; 2 = Some [=0]; 3 = Not very much [=1]; 4 = None at all [=1]	Trust
Comparative Cross-National Study of Religious Fundamentalism, Developmental Idealism, Values, and Morality in the Middle East and North Africa	I am going to name a number of groups. For each one, could you tell me how much trust, in general, you have in them: is it a great deal of trust, some trust, not very much trust or none at all? Followers of other religions.	1 = A great deal [=0]; 2 = Some [=0]; 3 = Not very much [=1]; 4 = None at all [=1]	Trust
Comparative Cross-National Study of Religious Fundamentalism, Developmental Idealism, Values, and Morality in the Middle East and North Africa	I am going to name a number of groups. For each one, could you tell me how much trust, in general, you have in them: is it a great deal of trust, some trust, not very much trust or none at all? Jews.	1 = A great deal [=0]; 2 = Some [=0]; 3 = Not very much [=1]; 4 = None at all [=1]	Trust
Comparative Cross-National Study of Religious Fundamentalism, Developmental Idealism, Values, and Morality in the Middle East and North Africa	I am going to name a number of groups. For each one, could you tell me how much trust, in general, you have in them: is it a great deal of trust, some trust, not very much trust or none at all? Shi'is.	1 = A great deal [=0]; 2 = Some [=0]; 3 = Not very much [=1]; 4 = None at all [=1]	Trust
Comparative Cross-National Study of Religious Fundamentalism, Developmental Idealism, Values, and Morality in the Middle East and North Africa	I am going to name a number of groups. For each one, could you tell me how much trust, in general, you have in them: is it a great deal of trust, some trust, not very much trust or none at all? Sunnis.	1 = A great deal [=0]; 2 = Some [=0]; 3 = Not very much [=1]; 4 = None at all [=1]	Trust
Comparative Cross-National Study of Religious Fundamentalism, Developmental Idealism, Values, and Morality in the Middle East and North Africa	I am going to name a number of individuals, groups and institutions. For each one, could you tell me how much trust, in general, you have in them: is it a lot, some, only a little, or none at all? Christians.	1 = A lot [=0]; 2 = Some [=0]; 3 = Only a little [=1]; 4 = Not at all [=1]	Trust
Comparative Cross-National Study of Religious Fundamentalism, Developmental Idealism, Values, and Morality in the Middle East and North Africa	I am going to name a number of individuals, groups and institutions. For each one, could you tell me how much trust, in general, you have in them: is it a lot, some, only a little, or none at all? Shia.	1 = A lot [=0]; 2 = Some [=0]; 3 = Only a little [=1]; 4 = Not at all [=1]	Trust
Comparative Cross-National Study of Religious Fundamentalism, Developmental Idealism, Values, and Morality in the Middle East and North Africa	I am going to name a number of individuals, groups and institutions. For each one, could you tell me how much trust, in general, you have in them: is it a lot, some, only a little, or none at all? Sunni.	1 = A lot [=0]; 2 = Some [=0]; 3 = Only a little [=1]; 4 = Not at all [=1]	Trust
Comparative Cross-National Study of Religious Fundamentalism, Developmental Idealism, Values, and Morality in the Middle East and North Africa	On this list are various groups of people. Could you please sort out any that you would not like to have as neighbors? Allawis.	1 = Don't like [=1]; 2 = Like [=0]	Role: neighbor

Table S12: (*continued*) List of unique question wordings and response-options.

Source(s)	Question text	Response options [recodes]	Question-type
Comparative Cross-National Study of Religious Fundamentalism, Developmental Idealism, Values, and Morality in the Middle East and North Africa	On this list are various groups of people. Could you please sort out any that you would not like to have as neighbors? Christians.	1 = Don't like [=1]; 2 = Like [=0]	Role: neighbor
Comparative Cross-National Study of Religious Fundamentalism, Developmental Idealism, Values, and Morality in the Middle East and North Africa	On this list are various groups of people. Could you please sort out any that you would not like to have as neighbors? Jews.	1 = Don't like [=1]; 2 = Like [=0]	Role: neighbor
Comparative Cross-National Study of Religious Fundamentalism, Developmental Idealism, Values, and Morality in the Middle East and North Africa	On this list are various groups of people. Could you please sort out any that you would not like to have as neighbors? Shi'is.	1 = Don't like [=1]; 2 = Like [=0]	Role: neighbor
Comparative Cross-National Study of Religious Fundamentalism, Developmental Idealism, Values, and Morality in the Middle East and North Africa	On this list are various groups of people. Could you please sort out any that you would not like to have as neighbors? Sunnis.	1 = Don't like [=1]; 2 = Like [=0]	Role: neighbor
Comparative Panel Survey on the Dynamics of Change: Belief Formation and Political Engagement in Egypt, Tunisia, and Turkey Wave 1	I am going to name a number of groups and institutions. For each one, could you tell me how much trust, in general, you have in them: is it a lot, some, only a little, or none at all? Jews.	1 = A lot [=0]; 2 = Some [=0]; 3 = Only a little [=1]; 4 = Not at all [=1]	Trust
Comparative Panel Survey on the Dynamics of Change: Belief Formation and Political Engagement in Egypt, Tunisia, and Turkey Wave 1	I am going to name a number of groups and institutions. For each one, could you tell me how much trust, in general, you have in them: is it a lot, some, only a little, or none at all? Shi'a.	1 = A lot [=0]; 2 = Some [=0]; 3 = Only a little [=1]; 4 = Not at all [=1]	Trust
Comparative Panel Survey on the Dynamics of Change: Belief Formation and Political Engagement in Egypt, Tunisia, and Turkey Wave 1; Comparative Panel Survey on the Dynamics of Change: Belief Formation and Political Engagement in Egypt, Tunisia, and Turkey Wave 2	I am going to name a number of groups and institutions. For each one, could you tell me how much trust, in general, you have in them: is it a lot, some, only a little, or none at all? Allawi.	1 = A lot [=0]; 2 = Some [=0]; 3 = Only a little [=1]; 4 = Not at all [=1]	Trust
Comparative Panel Survey on the Dynamics of Change: Belief Formation and Political Engagement in Egypt, Tunisia, and Turkey Wave 1; Comparative Panel Survey on the Dynamics of Change: Belief Formation and Political Engagement in Egypt, Tunisia, and Turkey Wave 2	I am going to name a number of groups and institutions. For each one, could you tell me how much trust, in general, you have in them: is it a lot, some, only a little, or none at all? Sunnis.	1 = A lot [=0]; 2 = Some [=0]; 3 = Only a little [=1]; 4 = Not at all [=1]	Trust
Comparative Panel Survey on the Dynamics of Change: Belief Formation and Political Engagement in Egypt, Tunisia, and Turkey Wave 2; Comparative Panel Survey on the Dynamics of Change: Belief Formation and Political Engagement in Egypt, Tunisia, and Turkey Wave 1	I am going to name a number of groups and institutions. For each one, could you tell me how much trust, in general, you have in them: is it a lot, some, only a little, or none at all? Christians.	1 = A lot [=0]; 2 = Some [=0]; 3 = Only a little [=1]; 4 = Not at all [=1]	Trust

Table S12: (*continued*) List of unique question wordings and response-options.

Source(s)	Question text	Response options [recodes]	Question-type
CROss-National Online Survey Wave 1	How much do you trust people of another religion?	1 = Trust completely [=0]; 2 = Trust somewhat [=0]; 3 = Do not trust very much [=1]; 4 = Do not trust at all [=1]	Trust
Eurobarometer Round 30	I am going to read out to you opinions. For each opinion I read out, please tell me to which, if any, kind of people it applies. To have them as neighbors creates problems. People of another religion.	0 = Not mentioned [=0]; 1 = Mentioned [=1]	Role: neighbor
Eurobarometer Round 30	I am going to read you out opinions. For each opinion I read out, please tell me to which, if any, kinds of people it applies. Marrying into one of these groups always ends badly. People of another religion.	0 = Not mentioned [=0]; 1 = Mentioned [=1]	Role: family
Eurobarometer Round 30	Some people are disturbed by the opinions, customs and way of life of people different from themselves. And do you find disturbing the presence of people of another religion?	1 = Disturbing [=1]; 2 = Not disturbing [=0]	Discomfort
Eurobarometer Round 37.0; Eurobarometer Round 39.0; Eurobarometer Round 42	Some people are disturbed by the opinions, customs and way of life of people different from themselves. And do you find the presence of people of another religion disturbing?	1 = Disturbing [=1]; 2 = Not Disturbing [=0]	Discomfort
Eurobarometer Round 47.2	Some people feel uneasy when they meet people who are different from themselves, for example, people who have different appearance, behaviour, opinions, habits or way of life. Do you feel uneasy in the presence of any people in your daily life? People of another religion.	0 = Not Mentioned [=0]; 1 = Mentioned [=1]	Discomfort
Eurobarometer Round 69.1	For each of the following situations, please tell me using this scale from 1 to 10 how you would personally feel about it: Having a person with a different religion or belief than yours as a neighbour.	1(/2/3/4/5) = Uncomfortable [=1]; (6/7/8/9)10 = Comfortable [=0]	Role: neighbor
Eurobarometer Round 83.4	Regardless of whether you are actually working or not, please tell me, using a scale from 1 to 10, how comfortable you would feel if one of your colleagues at work belonged to each of the following groups? A Buddhist person.	1(/2/3/4/5) = Uncomfortable [=1]; (6/7/8/9)10 = Comfortable [=0]	Role: work
Eurobarometer Round 83.4	Regardless of whether you are actually working or not, please tell me, using a scale from 1 to 10, how comfortable you would feel if one of your colleagues at work belonged to each of the following groups? A Muslim person.	1(/2/3/4/5) = Uncomfortable [=1]; (6/7/8/9)10 = Comfortable [=0]	Role: work
Eurobarometer Round 83.4; Eurobarometer Round 91.4	Regardless of whether you are actually working or not, please tell me, using a scale from 1 to 10, how comfortable you would feel if one of your colleagues at work belonged to each of the following groups? A Christian person.	1(/2/3/4/5) = Uncomfortable [=1]; (6/7/8/9)10 = Comfortable [=0]	Role: work
Eurobarometer Round 83.4; Eurobarometer Round 91.4	Regardless of whether you are actually working or not, please tell me, using a scale from 1 to 10, how comfortable you would feel if one of your colleagues at work belonged to each of the following groups? A Jewish person.	1(/2/3/4/5) = Uncomfortable [=1]; (6/7/8/9)10 = Comfortable [=0]	Role: work
Eurobarometer Round 83.4; Eurobarometer Round 91.4	Regardless of whether you have children or not, please tell me, using a scale from 1 to 10, how comfortable you would feel if one of your children was in a love relationship with a person from each of the following groups? A Buddhist person.	1(/2/3/4/5) = Uncomfortable [=1]; (6/7/8/9)10 = Comfortable [=0]	Role: family
Eurobarometer Round 83.4; Eurobarometer Round 91.4	Regardless of whether you have children or not, please tell me, using a scale from 1 to 10, how comfortable you would feel if one of your children was in a love relationship with a person from each of the following groups? A Christian person.	1(/2/3/4/5) = Uncomfortable [=1]; (6/7/8/9)10 = Comfortable [=0]	Role: family
Eurobarometer Round 83.4; Eurobarometer Round 91.4	Regardless of whether you have children or not, please tell me, using a scale from 1 to 10, how comfortable you would feel if one of your children was in a love relationship with a person from each of the following groups? A Jewish person.	1(/2/3/4/5) = Uncomfortable [=1]; (6/7/8/9)10 = Comfortable [=0]	Role: family
Eurobarometer Round 83.4; Eurobarometer Round 91.4	Regardless of whether you have children or not, please tell me, using a scale from 1 to 10, how comfortable you would feel if one of your children was in a love relationship with a person from each of the following groups? A Muslim person.	1(/2/3/4/5) = Uncomfortable [=1]; (6/7/8/9)10 = Comfortable [=0]	Role: family

Table S12: (*continued*) List of unique question wordings and response-options.

Source(s)	Question text	Response options [recodes]	Question-type
Eurobarometer Round 91.4	Regardless of whether you are actually working or not, please tell me, using a scale from 1 to 10, how comfortable you would feel if one of your colleagues at work belonged to each of the following groups? A Buddhist person.	1(/2/3/4/5) = Uncomfortable [=1]; 6-10 = Comfortable [=0]	Role: work
Eurobarometer Round 91.4	Regardless of whether you are actually working or not, please tell me, using a scale from 1 to 10, how comfortable you would feel if one of your colleagues at work belonged to each of the following groups? Muslim person.	1(/2/3/4/5) = Uncomfortable [=1]; (6/7/8/9)10 = Comfortable [=0]	Role: work
European Values Study Wave 5	I would like to ask you how much you trust people from various groups. Could you tell me for each whether you trust people from this group completely, somewhat, not very much or not at all? People of another religion.	1 = Trust completely [=0]; 2 = Trust somewhat [=0]; 3 = Do not trust very much [=1]; 4 = Do not trust at all [=1]	Trust
Friedrich-Ebert-Stiftung Youth Studies in East Europe and Central Asia	I will read a list to you with various groups or persons. Please tell me how much confidence you have in each group or person below, on a scale of 1 (no confidence at all) to 10 (total confidence). Persons who have another religion than you.	1(2/3/4/5) = No confidence at all [=1]; (6/7/8/9)10 = Total confidence [=0]	Trust
Friedrich-Ebert-Stiftung Youth Studies in East Europe and Central Asia	Imagine a scale from 1 to 10 where in the first scale (1) are the people you trust less and in the last scale (the 10th) are the people you trust more. According to you, at which scale would the following people belong? People of other religions.	1(2/3/4/5) = Trust less [=1]; (6/7/8/9)10 = Trust most [=0]	Trust
Friedrich-Ebert-Stiftung Youth Studies in East Europe and Central Asia	Imagine a scale from 1 to 10 where value one (1) indicates people you trust the least, and value ten (10) indicates people you trust the most. Where do, in your opinion, the following people belong on such scale? People of different religions from your own.	1(2/3/4/5) = Trust least [=1]; (6/7/8/9)10 = Trust most [=0]	Trust
Friedrich-Ebert-Stiftung Youth Studies in East Europe and Central Asia	Please imagine a scale with values ranging from 1 to 10, where 1 stands for people who you trust least, and 10 for people who you trust most. Where on this scale would you put the following people? People of other religions.	1(2/3/4/5) = Trust least [=1]; (6/7/8/9)10 = Trust most [=0]	Trust
Friedrich-Ebert-Stiftung Youth Studies in East Europe and Central Asia	Please tell me to what extent you trust the following people. Imagine a scale from 1 to 10, where 1 means you don't trust them completely, 10 means you trust them completely. People of other religions.	1(2/3/4/5) = Don't trust completely [=1]; (6/7/8/9)10 = Trust completely [=0]	Trust
Friedrich-Ebert-Stiftung Youth Studies in East Europe and Central Asia	To what degree do you trust the following people? Imagine a scale from 1 to 5, where 1 means no trust at all and 5 very much. People of other religions.	1(2) = No trust at all [=1]; (3/4)5 = Very much trust [=0]	Trust
Friedrich-Ebert-Stiftung Youth Studies in East Europe and Central Asia	Where on this scale would you put the following people? Please follow the scale from 1 (trust least) to 10 (trust most). People of other religions.	1(2/3/4/5) = Trust least [=1]; (6/7/8/9)10 = Trust most [=0]	Trust
Friedrich-Ebert-Stiftung Youth Studies in Southeast Europe	To what degree to you trust the following people? People of other religions.	1(/2) = Not at all [=1]; (3/4/)5 = Very much [=0]	Trust
International Social Survey Programme Religion III	People have different religions and different religious views. Would you accept a person from a different religion or with a different religious view from yours marrying a relative of yours?	1 = Definitely accept [=0]; 2 = Probably accept [=0]; 3 = Probably not accept [=1]; 4 = Definitely not accept [=1]	Role: family
International Social Survey Programme Religion IV	Do you consider those belonging to the following groups as threatening or not threatening? Buddhists.	1 = Very threatening [=1]; 2 = Somewhat threatening [=1]; 3 = Not very threatening [=0]; 4 = Not at all threatening [=0]	Discomfort
International Social Survey Programme Religion IV	Do you consider those belonging to the following groups as threatening or not threatening? Christians.	1 = Very threatening [=1]; 2 = Somewhat threatening [=1]; 3 = Not very threatening [=0]; 4 = Not at all threatening [=0]	Discomfort
International Social Survey Programme Religion IV	Do you consider those belonging to the following groups as threatening or not threatening? Hindus.	1 = Very threatening [=1]; 2 = Somewhat threatening [=1]; 3 = Not very threatening [=0]; 4 = Not at all threatening [=0]	Discomfort

Table S12: (*continued*) List of unique question wordings and response-options.

Source(s)	Question text	Response options [recodes]	Question-type
International Social Survey Programme Religion IV	Do you consider those belonging to the following groups as threatening or not threatening? Jews.	1 = Very threatening [=1]; 2 = Somewhat threatening [=1]; 3 = Not very threatening [=0]; 4 = Not at all threatening [=0]	Discomfort
International Social Survey Programme Religion IV	Do you consider those belonging to the following groups as threatening or not threatening? Muslims.	1 = Very threatening [=1]; 2 = Somewhat threatening [=1]; 3 = Not very threatening [=0]; 4 = Not at all threatening [=0]	Discomfort
International Social Survey Programme Religion IV	People have different religions and different religious views. Would you accept a person from a different religion or with a very different religious view from yours marrying a relative of yours?	1 = Definitely accept [=0]; 2 = Probably accept [=0]; 3 = Probably not accept [=1]; 4 = Definitely not accept [=1]	Role: family
International Social Survey Programme Religion IV; International Social Survey Programme Religion III	What is your personal attitude towards members of the following religious groups? Buddhists.	1 = Very positive [=0]; 2 = Somewhat positive [=0]; 3 = Neither positive nor negative [=0]; 4 = Somewhat negative [=1]; 5 = Very negative [=1]	Favorability
International Social Survey Programme Religion IV; International Social Survey Programme Religion III	What is your personal attitude towards members of the following religious groups? Christians.	1 = Very positive [=0]; 2 = Somewhat positive [=0]; 3 = Neither positive nor negative [=0]; 4 = Somewhat negative [=1]; 5 = Very negative [=1]	Favorability
International Social Survey Programme Religion IV; International Social Survey Programme Religion III	What is your personal attitude towards members of the following religious groups? Hindus.	1 = Very positive [=0]; 2 = Somewhat positive [=0]; 3 = Neither positive nor negative [=0]; 4 = Somewhat negative [=1]; 5 = Very negative [=1]	Favorability
International Social Survey Programme Religion IV; International Social Survey Programme Religion III	What is your personal attitude towards members of the following religious groups? Jews.	1 = Very positive [=0]; 2 = Somewhat positive [=0]; 3 = Neither positive nor negative [=0]; 4 = Somewhat negative [=1]; 5 = Very negative [=1]	Favorability
International Social Survey Programme Religion IV; International Social Survey Programme Religion III	What is your personal attitude towards members of the following religious groups? Muslims.	1 = Very positive [=0]; 2 = Somewhat positive [=0]; 3 = Neither positive nor negative [=0]; 4 = Somewhat negative [=1]; 5 = Very negative [=1]	Favorability
LatinoBarometero 1998	In this list you will see various groups of people. Could you select any that you would not like to have as neighbours? Jews.	1 = Mentioned [=1]; 0 = Not mentioned [=0]	Role: neighbor
LatinoBarometero 1998	In this list you will see various groups of people. Could you select any that you would not like to have as neighbours? Muslims.	1 = Mentioned [=1]; 0 = Not mentioned [=0]	Role: neighbor
Life in Transition Survey Round 2; Life in Transition Survey Round 3	On this list are various groups of people. Could you please mention any that you would not like to have as neighbours? Please just read out the letter that applies. Jewish people.	0 = Not mentioned [=0]; 1 = Mentioned [=1]	Role: neighbor
Life in Transition Survey Round 2; Life in Transition Survey Round 3	On this list are various groups of people. Could you please mention any that you would not like to have as neighbours? Please just read out the letter that applies. People of a different religion.	0 = Not mentioned [=0]; 1 = Mentioned [=1]	Role: neighbor
Perception and Acceptance of Religious Diversity among the European Population	How pleasant do you find these contacts with Buddhists?	1 = Very pleasant [=0]; 2 = Somewhat pleasant [=0]; 3 = Somewhat unpleasant [=1]; 4 = Very unpleasant [=1]	Discomfort
Perception and Acceptance of Religious Diversity among the European Population	How pleasant do you find these contacts with Christians?	1 = Very pleasant [=0]; 2 = Somewhat pleasant [=0]; 3 = Somewhat unpleasant [=1]; 4 = Very unpleasant [=1]	Discomfort

Table S12: (*continued*) List of unique question wordings and response-options.

Source(s)	Question text	Response options [recodes]	Question-type
Perception and Acceptance of Religious Diversity among the European Population	How pleasant do you find these contacts with Hindus?	1 = Very pleasant [=0]; 2 = Somewhat pleasant [=0]; 3 = Somewhat unpleasant [=1]; 4 = Very unpleasant [=1]	Discomfort
Perception and Acceptance of Religious Diversity among the European Population	How pleasant do you find these contacts with Jews?	1 = Very pleasant [=0]; 2 = Somewhat pleasant [=0]; 3 = Somewhat unpleasant [=1]; 4 = Very unpleasant [=1]	Discomfort
Perception and Acceptance of Religious Diversity among the European Population	How pleasant do you find these contacts with Muslims?	1 = Very pleasant [=0]; 2 = Somewhat pleasant [=0]; 3 = Somewhat unpleasant [=1]; 4 = Very unpleasant [=1]	Discomfort
Perception and Acceptance of Religious Diversity among the European Population	What is your personal attitude toward members of the following religious groups? Buddhists.	1 = Very positive [=0]; 2 = Somewhat positive [=0]; 3 = Somewhat negative [=1]; 4 = Very negative [=1]	Favorability
Perception and Acceptance of Religious Diversity among the European Population	What is your personal attitude toward members of the following religious groups? Christians.	1 = Very positive [=0]; 2 = Somewhat positive [=0]; 3 = Somewhat negative [=1]; 4 = Very negative [=1]	Favorability
Perception and Acceptance of Religious Diversity among the European Population	What is your personal attitude toward members of the following religious groups? Hindus.	1 = Very positive [=0]; 2 = Somewhat positive [=0]; 3 = Somewhat negative [=1]; 4 = Very negative [=1]	Favorability
Perception and Acceptance of Religious Diversity among the European Population	What is your personal attitude toward members of the following religious groups? Jews.	1 = Very positive [=0]; 2 = Somewhat positive [=0]; 3 = Somewhat negative [=1]; 4 = Very negative [=1]	Favorability
Perception and Acceptance of Religious Diversity among the European Population	What is your personal attitude toward members of the following religious groups? Muslims.	1 = Very positive [=0]; 2 = Somewhat positive [=0]; 3 = Somewhat negative [=1]; 4 = Very negative [=1]	Favorability
Pew Global Attitudes Project Fall 2009	I'd like you to rate some different groups of people in (survey country) according to how you feel about them. For each group, please tell me whether your opinion is very favorable, mostly favorable, mostly unfavorable or very unfavorable? Catholics.	1 = Very favorable [=0]; 2 = Mostly favorable [=0]; 3 = Mostly unfavorable [=1]; 4 = Very unfavorable [=1]	Favorability
Pew Global Attitudes Project Fall 2009	I'd like you to rate some different groups of people in (survey country) according to how you feel about them. For each group, please tell me whether your opinion is very favorable, mostly favorable, mostly unfavorable or very unfavorable? Christians.	1 = Very favorable [=0]; 2 = Mostly favorable [=0]; 3 = Mostly unfavorable [=1]; 4 = Very unfavorable [=1]	Favorability
Pew Global Attitudes Project Fall 2009	I'd like you to rate some different groups of people in (survey country) according to how you feel about them. For each group, please tell me whether your opinion is very favorable, mostly favorable, mostly unfavorable or very unfavorable? Hindus.	1 = Very favorable [=0]; 2 = Mostly favorable [=0]; 3 = Mostly unfavorable [=1]; 4 = Very unfavorable [=1]	Favorability
Pew Global Attitudes Project Fall 2009	I'd like you to rate some different groups of people in (survey country) according to how you feel about them. For each group, please tell me whether your opinion is very favorable, mostly favorable, mostly unfavorable or very unfavorable? Jews.	1 = Very favorable [=0]; 2 = Mostly favorable [=0]; 3 = Mostly unfavorable [=1]; 4 = Very unfavorable [=1]	Favorability
Pew Global Attitudes Project Fall 2009	I'd like you to rate some different groups of people in (survey country) according to how you feel about them. For each group, please tell me whether your opinion is very favorable, mostly favorable, mostly unfavorable or very unfavorable? Muslim Bulgarians.	1 = Very favorable [=0]; 2 = Mostly favorable [=0]; 3 = Mostly unfavorable [=1]; 4 = Very unfavorable [=1]	Favorability
Pew Global Attitudes Project Fall 2009	I'd like you to rate some different groups of people in (survey country) according to how you feel about them. For each group, please tell me whether your opinion is very favorable, mostly favorable, mostly unfavorable or very unfavorable? Muslims.	1 = Very favorable [=0]; 2 = Mostly favorable [=0]; 3 = Mostly unfavorable [=1]; 4 = Very unfavorable [=1]	Favorability

Table S12: (*continued*) List of unique question wordings and response-options.

Source(s)	Question text	Response options [recodes]	Question-type
Pew Global Attitudes Project Fall 2009	I'd like you to rate some different groups of people who live in (survey country) according to how you feel about them. For each group, please tell me whether your opinion is very favorable, mostly favorable, mostly unfavorable or very unfavorable? Jews.	1 = Very favorable [=0]; 2 = Mostly favorable [=0]; 3 = Mostly unfavorable [=1]; 4 = Very unfavorable [=1]	Favorability
Pew Global Attitudes Project Spring 2004; Pew Global Attitudes Project Spring 2005; Pew Global Attitudes Project Spring 2009; Pew Global Attitudes Project Spring 2010; Pew Global Attitudes Project Spring 2011	Please tell me if you have a very favorable, somewhat favorable, somewhat unfavorable or very unfavorable opinion of Christians?	1 = Very favorable [=0]; 2 = Somewhat favorable [=0]; 3 = Somewhat unfavorable [=1]; 4 = Very unfavorable [=1]	Favorability
Pew Global Attitudes Project Spring 2004; Pew Global Attitudes Project Spring 2005; Pew Global Attitudes Project Spring 2009; Pew Global Attitudes Project Spring 2010; Pew Global Attitudes Project Spring 2011	Please tell me if you have a very favorable, somewhat favorable, somewhat unfavorable or very unfavorable opinion of Jews?	1 = Very favorable [=0]; 2 = Somewhat favorable [=0]; 3 = Somewhat unfavorable [=1]; 4 = Very unfavorable [=1]	Favorability
Pew Global Attitudes Project Spring 2004; Pew Global Attitudes Project Spring 2005; Pew Global Attitudes Project Spring 2009; Pew Global Attitudes Project Spring 2010; Pew Global Attitudes Project Spring 2011	Please tell me if you have a very favorable, somewhat favorable, somewhat unfavorable or very unfavorable opinion of Muslims?	1 = Very favorable [=0]; 2 = Somewhat favorable [=0]; 3 = Somewhat unfavorable [=1]; 4 = Very unfavorable [=1]	Favorability
Pew Global Attitudes Project Spring 2006	Please tell me if you have a very favorable, somewhat favorable, somewhat unfavorable, or very unfavorable opinion of (INSERT)? Christians.	1 = Very favorable [=0]; 2 = Somewhat favorable [=0]; 3 = Somewhat unfavorable [=1]; 4 = Very unfavorable [=1]	Favorability
Pew Global Attitudes Project Spring 2006	Please tell me if you have a very favorable, somewhat favorable, somewhat unfavorable, or very unfavorable opinion of (INSERT)? Jews.	1 = Very favorable [=0]; 2 = Somewhat favorable [=0]; 3 = Somewhat unfavorable [=1]; 4 = Very unfavorable [=1]	Favorability
Pew Global Attitudes Project Spring 2006	Please tell me if you have a very favorable, somewhat favorable, somewhat unfavorable, or very unfavorable opinion of (INSERT)? Muslims.	1 = Very favorable [=0]; 2 = Somewhat favorable [=0]; 3 = Somewhat unfavorable [=1]; 4 = Very unfavorable [=1]	Favorability
Pew Global Attitudes Project Spring 2008	On a different topic, please tell me if you have a very favorable, somewhat favorable, somewhat unfavorable or very unfavorable opinion of Christians?	1 = Very favorable [=0]; 2 = Somewhat favorable [=0]; 3 = Somewhat unfavorable [=1]; 4 = Very unfavorable [=1]	Favorability
Pew Global Attitudes Project Spring 2008	On a different topic, please tell me if you have a very favorable, somewhat favorable, somewhat unfavorable or very unfavorable opinion of Jews?	1 = Very favorable [=0]; 2 = Somewhat favorable [=0]; 3 = Somewhat unfavorable [=1]; 4 = Very unfavorable [=1]	Favorability
Pew Global Attitudes Project Spring 2008	On a different topic, please tell me if you have a very favorable, somewhat favorable, somewhat unfavorable or very unfavorable opinion of Muslims?	1 = Very favorable [=0]; 2 = Somewhat favorable [=0]; 3 = Somewhat unfavorable [=1]; 4 = Very unfavorable [=1]	Favorability
Pew Global Attitudes Project Spring 2014; Pew Global Attitudes Project Spring 2015; Pew Global Attitudes Project Spring 2016	I'd like you to rate some different groups of people in (survey country) according to how you feel about them. For each group, please tell me whether your opinion is very favorable, mostly favorable, mostly unfavorable or very unfavorable. Jews.	1 = Very favorable [=0]; 2 = Mostly favorable [=0]; 3 = Mostly unfavorable [=1]; 4 = Very unfavorable [=1]	Favorability
Pew Global Attitudes Project Spring 2014; Pew Global Attitudes Project Spring 2015; Pew Global Attitudes Project Spring 2016	I'd like you to rate some different groups of people in (survey country) according to how you feel about them. For each group, please tell me whether your opinion is very favorable, mostly favorable, mostly unfavorable or very unfavorable. Muslims.	1 = Very favorable [=0]; 2 = Mostly favorable [=0]; 3 = Mostly unfavorable [=1]; 4 = Very unfavorable [=1]	Favorability
Pew Global Attitudes Project Spring 2019	I'd like you to rate some different groups of people in (survey country) according to how you feel about them. For each group, please tell me whether your opinion of them is very favorable, mostly favorable, mostly unfavorable or very unfavorable. How about Jews?	1 = Very favorable [=0]; 2 = Mostly favorable [=0]; 3 = Mostly unfavorable [=1]; 4 = Very unfavorable [=1]	Favorability

Table S12: (*continued*) List of unique question wordings and response-options.

Source(s)	Question text	Response options [recodes]	Question-type
Pew Global Attitudes Project Spring 2019	I'd like you to rate some different groups of people in (survey country) according to how you feel about them. For each group, please tell me whether your opinion of them is very favorable, mostly favorable, mostly unfavorable or very unfavorable. How about Muslim Bulgarians?	1 = Very favorable [=0]; 2 = Mostly favorable; 3 = Mostly unfavorable [=1]; 4 = Very unfavorable	Favorability
Pew Global Attitudes Project Spring 2019	I'd like you to rate some different groups of people in (survey country) according to how you feel about them. For each group, please tell me whether your opinion of them is very favorable, mostly favorable, mostly unfavorable or very unfavorable. How about Muslims?	1 = Very favorable [=0]; 2 = Mostly favorable; 3 = Mostly unfavorable [=1]; 4 = Very unfavorable	Favorability
Pew Religious Belief and National Belonging in Central and Eastern Europe	Would you be willing to accept Catholics as citizens of our country?	1 = Yes [=0]; 2 = No [=1]	Role: citizen
Pew Religious Belief and National Belonging in Central and Eastern Europe	Would you be willing to accept Catholics as members of your family?	1 = Yes [=0]; 2 = No [=1]	Role: family
Pew Religious Belief and National Belonging in Central and Eastern Europe	Would you be willing to accept Catholics as neighbors?	1 = Yes [=0]; 2 = No [=1]	Role: neighbor
Pew Religious Belief and National Belonging in Central and Eastern Europe	Would you be willing to accept Jews as neighbors?	1 = Yes [=0]; 2 = No [=1]	Role: neighbor
Pew Religious Belief and National Belonging in Central and Eastern Europe	Would you be willing to accept Jews as citizens of our country?	1 = Yes [=0]; 2 = No [=1]	Role: citizen
Pew Religious Belief and National Belonging in Central and Eastern Europe	Would you be willing to accept Jews as members of your family?	1 = Yes [=0]; 2 = No [=1]	Role: family
Pew Religious Belief and National Belonging in Central and Eastern Europe	Would you be willing to accept Muslims as citizens of our country?	1 = Yes [=0]; 2 = No [=1]	Role: citizen
Pew Religious Belief and National Belonging in Central and Eastern Europe	Would you be willing to accept Muslims as members of your family?	1 = Yes [=0]; 2 = No [=1]	Role: family
Pew Religious Belief and National Belonging in Central and Eastern Europe	Would you be willing to accept Muslims as neighbors?	1 = Yes [=0]; 2 = No [=1]	Role: neighbor
Pew Religious Belief and National Belonging in Central and Eastern Europe	Would you be willing to accept Orthodox Christians as citizens of our country?	1 = Yes [=0]; 2 = No [=1]	Role: citizen
Pew Religious Belief and National Belonging in Central and Eastern Europe	Would you be willing to accept Orthodox Christians as members of your family?	1 = Yes [=0]; 2 = No [=1]	Role: family
Pew Religious Belief and National Belonging in Central and Eastern Europe	Would you be willing to accept Orthodox Christians as neighbors?	1 = Yes [=0]; 2 = No [=1]	Role: neighbor
Pew: Islam and Christianity in Sub-Saharan Africa	And which comes closer to describing your view? I generally trust people who have different religious values than me, OR I generally do not trust people who have different religious values than me?	1 = I generally trust people who have different religious values than me [=0]; 2 = I generally do not trust people who have different religious values than me [=1]	Trust
Pew: Islam and Christianity in Sub-Saharan Africa	How comfortable would you be if a child of yours someday married a Christian?	1 = Very comfortable [=0]; 2 = Somewhat comfortable [=0]; 3 = Not too comfortable [=1]; 4 = Not at all comfortable [=1]	Role: family
Pew: Islam and Christianity in Sub-Saharan Africa	How comfortable would you be if a child of yours someday married a Muslim?	1 = Very comfortable [=0]; 2 = Somewhat comfortable [=0]; 3 = Not too comfortable [=1]; 4 = Not at all comfortable [=1]	Role: family

Table S12: (*continued*) List of unique question wordings and response-options.

Source(s)	Question text	Response options [recodes]	Question-type
Pew: The World's Muslims—Religion, Politics and Society	How comfortable would you be if a daughter of yours someday married a Buddhist?	1 = Very comfortable [=0]; 2 = Somewhat comfortable [=0]; 3 = Not too comfortable [=1]; 4 = Not at all comfortable [=1]	Role: family
Pew: The World's Muslims—Religion, Politics and Society	How comfortable would you be if a daughter of yours someday married a Christian?	1 = Very comfortable [=0]; 2 = Somewhat comfortable [=0]; 3 = Not too comfortable [=1]; 4 = Not at all comfortable [=1]	Role: family
Pew: The World's Muslims—Religion, Politics and Society	How comfortable would you be if a son of yours someday married a Buddhist?	1 = Very comfortable [=0]; 2 = Somewhat comfortable [=0]; 3 = Not too comfortable [=1]; 4 = Not at all comfortable [=1]	Role: family
Pew: The World's Muslims—Religion, Politics and Society	How comfortable would you be if a son of yours someday married a Christian?	1 = Very comfortable [=0]; 2 = Somewhat comfortable [=0]; 3 = Not too comfortable [=1]; 4 = Not at all comfortable [=1]	Role: family
Pew: The World's Muslims—Religion, Politics and Society	Suppose you heard that someone's daughter married a Christian. Would you totally approve, somewhat approve, somewhat disapprove or totally disapprove of such a marriage?	1 = Totally approve [=0]; 2 = Somewhat approve [=0]; 3 = Somewhat disapprove [=1]; 4 = Totally disapprove [=1]	Role: family
Pew: The World's Muslims—Religion, Politics and Society	Suppose you heard that someone's son married a Christian. Would you totally approve, somewhat approve, somewhat disapprove or totally disapprove of such a marriage?	1 = Totally approve [=0]; 2 = Somewhat approve [=0]; 3 = Somewhat disapprove [=1]; 4 = Totally disapprove [=1]	Role: family
Pulse of Europe	I'd like you to rate some different groups of people in (country) according to how you feel about them. For each group, please tell me whether your opinion is very favorable, mostly favorable, mostly unfavorable, or very unfavorable? Jews.	1 = Very favorable [=0]; 2 = Mostly favorable [=0]; 3 = Mostly unfavorable [=1]; Very unfavorable [=1]	Favorability
Pulse of Europe	I'd like you to rate some different groups of people in (country) according to how you feel about them. For each group, please tell me whether your opinion is very favorable, mostly favorable, mostly unfavorable, or very unfavorable? Muslim Bulgarians	1 = Very favorable [=0]; 2 = Mostly favorable [=0]; 3 = Mostly unfavorable [=1]; Very unfavorable [=1]	Favorability
Second European Union Minorities and Discrimination Survey	Using a scale from 0 to 10, please tell me how you would feel about having someone from one of the following groups as your neighbour? A person who has a different religion than yours.	0(1/2/3/4) = Uncomfortable [=1]; (5/6/7/8/9)10 = Comfortable [=0]	Role: neighbor
Second European Union Minorities and Discrimination Survey	Using a scale from 0 to 10, please tell me how you would feel about someone from your family being married to a person from one of the following groups? A person who has a different religion than yours.	0(1/2/3/4) = Uncomfortable [=1]; (5/6/7/8/9)10 = Comfortable [=0]	Role: family
The Youth, Emotional Energy, and Political Violence: The Cases of Egypt and Saudi Arabia	If it were possible, I'd rather have a job where I worked with people with the same religious views I have rather than with people with different views.	1 = Strongly agree [=1]; 2 = Agree [=1]; 3 Disagree [=0]; 4 = Strongly disagree [=0]	Role: work
World Values Survey Wave 2	On this list are various groups of people. Could you identify any that you would not like to have as neighbours? Hindus.	0 = Not mentioned [=0]; 1 = Mentioned [=1]	Role: neighbor
World Values Survey Wave 2; World Values Survey Wave 3; World Values Survey Wave 4; World Values Survey Wave 5	On this list are various groups of people. Could you identify any that you would not like to have as neighbours? Jews.	0 = Not mentioned [=0]; 1 = Mentioned [=1]	Role: neighbor
World Values Survey Wave 3; World Values Survey Wave 4; World Values Survey Wave 5	On this list are various groups of people. Could you identify any that you would not like to have as neighbours? Christians.	0 = Not mentioned [=0]; 1 = Mentioned [=1]	Role: neighbor
World Values Survey Wave 4	On this list are various groups of people. Could you identify any that you would not like to have as neighbours? Protestants.	0 = Not mentioned [=0]; 1 = Mentioned [=1]	Role: neighbor

Table S12: (*continued*) List of unique question wordings and response-options.

Source(s)	Question text	Response options [recodes]	Question-type
World Values Survey Wave 4; World Values Survey Wave 5	On this list are various groups of people. Could you identify any that you would not like to have as neighbours? Shia.	0 = Not mentioned [=0]; 1 = Mentioned [=1]	Role: neighbor
World Values Survey Wave 4; World Values Survey Wave 5	On this list are various groups of people. Could you identify any that you would not like to have as neighbours? Sunnis.	0 = Not mentioned [=0]; 1 = Mentioned [=1]	Role: neighbor
World Values Survey Wave 5; World Values Survey Wave 2; World Values Survey Wave 3; World Values Survey Wave 4; World Values Survey Wave 1	On this list are various groups of people. Could you identify any that you would not like to have as neighbours? Muslims.	0 = Not mentioned [=0]; 1 = Mentioned [=1]	Role: neighbor
World Values Survey Wave 5; World Values Survey Wave 3; World Values Survey Wave 6; World Values Survey Wave 7; World Values Survey Wave 4	On this list are various groups of people. Could you identify any that you would not like to have as neighbours? People of a different religion.	0 = Not mentioned [=0]; 1 = Mentioned [=1]	Role: neighbor

Table S13: List of surveys and coverage by country.

Country	Source(s)	Range of years covered	N
Albania	Friedrich-Ebert-Stiftung Youth Studies in Southeast Europe; World Values Survey Wave 3; World Values Survey Wave 4; Life in Transition Survey Round 2; Life in Transition Survey Round 3; Pew: The World's Muslims—Religion, Politics and Society	1998-2018	9,539
Algeria	World Values Survey Wave 4; World Values Survey Wave 6; International Social Survey Programme Religion IV; Pew: The World's Muslims—Religion, Politics and Society; Arab Barometer Wave 2; Arab Barometer Wave 4; Arab Barometer Wave 5; Arab Barometer Wave 6; Arab Opinion Index Wave 7	2002-2021	30,571
Andorra	World Values Survey Wave 5; World Values Survey Wave 7	2005-2018	1,289
Argentina	Pew Global Attitudes Project Spring 2008; Pew Global Attitudes Project Spring 2009; Pew Global Attitudes Project Spring 2010; World Values Survey Wave 2; World Values Survey Wave 3; World Values Survey Wave 4; World Values Survey Wave 5; World Values Survey Wave 6; World Values Survey Wave 7; LatinoBarometero 1998	1991-2017	17,351
Armenia	Caucasus Barometer 2019; Caucasus Barometer 2017; Caucasus Barometer 2015; Caucasus Barometer 2013; Caucasus Barometer 2012; Caucasus Barometer 2011; Caucasus Barometer 2010; Caucasus Barometer 2009; World Values Survey Wave 6; World Values Survey Wave 7; Life in Transition Survey Round 2; Life in Transition Survey Round 3; Pew Religious Belief and National Belonging in Central and Eastern Europe	2009-2021	67,098
Australia	Pew Global Attitudes Project Spring 2008; World Values Survey Wave 5; World Values Survey Wave 6; World Values Survey Wave 7; International Social Survey Programme Religion III	2005-2018	7,807
Austria	International Social Survey Programme Religion III; International Social Survey Programme Religion IV; Second European Union Minorities and Discrimination Survey; Eurobarometer Round 47.2; Eurobarometer Round 69.1; Eurobarometer Round 83.4; Eurobarometer Round 91.4	1997-2019	16,718
Azerbaijan	Caucasus Barometer 2013; Caucasus Barometer 2012; Caucasus Barometer 2011; Caucasus Barometer 2010; Caucasus Barometer 2009; World Values Survey Wave 3; World Values Survey Wave 6; Life in Transition Survey Round 2; Life in Transition Survey Round 3; Pew: The World's Muslims—Religion, Politics and Society	1997-2016	28,471
Bahrain	Arab Barometer Wave 1	2009	420
Bangladesh	World Values Survey Wave 3; World Values Survey Wave 4; World Values Survey Wave 7; Pew: The World's Muslims—Religion, Politics and Society	1996-2018	8,830
Belarus	World Values Survey Wave 3; World Values Survey Wave 6; Life in Transition Survey Round 2; Life in Transition Survey Round 3; Pew Religious Belief and National Belonging in Central and Eastern Europe	1996-2016	17,477
Belgium	International Social Survey Programme Religion III; Second European Union Minorities and Discrimination Survey; Eurobarometer Round 30; Eurobarometer Round 37.0; Eurobarometer Round 39.0; Eurobarometer Round 42; Eurobarometer Round 47.2; Eurobarometer Round 69.1; Eurobarometer Round 83.4; Eurobarometer Round 91.4	1988-2019	20,407
Benin	Afrobarometer Round 6; Afrobarometer Round 7	2014-2017	2,318
Bolivia	World Values Survey Wave 7; LatinoBarometero 1998	1998-2017	2,998
Bosnia	Friedrich-Ebert-Stiftung Youth Studies in Southeast Europe; World Values Survey Wave 3; World Values Survey Wave 4; Life in Transition Survey Round 2; Life in Transition Survey Round 3; Pew Religious Belief and National Belonging in Central and Eastern Europe; Pew: The World's Muslims—Religion, Politics and Society	1998-2018	17,939
Botswana	Pew: Islam and Christianity in Sub-Saharan Africa; Afrobarometer Round 6; Afrobarometer Round 7	2009-2017	3,671

Table S13: (*continued*) List of surveys and coverage by country.

Country	Source(s)	Range of years covered	N
Brazil	Pew Global Attitudes Project Spring 2008; Pew Global Attitudes Project Spring 2009; Pew Global Attitudes Project Spring 2010; Pew Global Attitudes Project Spring 2011; World Values Survey Wave 2; World Values Survey Wave 3; World Values Survey Wave 5; World Values Survey Wave 6; World Values Survey Wave 7; LatinoBarometero 1998	1991-2018	20,685
Bulgaria	Pew Global Attitudes Project Fall 2009; Pew Global Attitudes Project Spring 2019; Friedrich-Ebert-Stiftung Youth Studies in Southeast Europe; Pulse of Europe; World Values Survey Wave 3; World Values Survey Wave 5; International Social Survey Programme Religion IV; Life in Transition Survey Round 2; Life in Transition Survey Round 3; Pew Religious Belief and National Belonging in Central and Eastern Europe; European Values Study Wave 5; Second European Union Minorities and Discrimination Survey; Friedrich-Ebert-Stiftung Youth Studies in East Europe and Central Asia; Candidate Eurobarometer 2002; Eurobarometer Round 69.1; Eurobarometer Round 83.4; Eurobarometer Round 91.4	1991-2019	43,357
Burkina Faso	World Values Survey Wave 5; Afrobarometer Round 6; Afrobarometer Round 7	2007-2017	3,848
Burundi	Afrobarometer Round 6	2014	1,189
Cambodia	International Social Survey Programme Religion IV	2018	5,887
Cameroon	Pew: Islam and Christianity in Sub-Saharan Africa; Afrobarometer Round 6; Afrobarometer Round 7	2008-2018	5,062
Canada	Pew Global Attitudes Project Spring 2005; Pew Global Attitudes Project Spring 2009; World Values Survey Wave 2; World Values Survey Wave 4; World Values Survey Wave 5; World Values Survey Wave 7	1990-2020	14,135
Cape Verde	Afrobarometer Round 6; Afrobarometer Round 7	2014-2017	1,973
Chad	Pew: Islam and Christianity in Sub-Saharan Africa	2009	2,683
Chile	World Values Survey Wave 2; World Values Survey Wave 3; World Values Survey Wave 4; World Values Survey Wave 5; World Values Survey Wave 6; World Values Survey Wave 7; International Social Survey Programme Religion III; International Social Survey Programme Religion IV; LatinoBarometero 1998	1990-2018	15,488
China	Pew Global Attitudes Project Spring 2005; Pew Global Attitudes Project Spring 2010; Pew Global Attitudes Project Spring 2011; Pew Global Attitudes Project Spring 2006; World Values Survey Wave 2; World Values Survey Wave 5; World Values Survey Wave 6; World Values Survey Wave 7	1990-2019	6,473
Colombia	World Values Survey Wave 5; World Values Survey Wave 6; World Values Survey Wave 7; LatinoBarometero 1998	1998-2018	6,939
Congo - Kinshasa	Pew: Islam and Christianity in Sub-Saharan Africa	2009	2,364
Costa Rica	LatinoBarometero 1998	1998	1,872
Côte d'Ivoire	Afrobarometer Round 6; Afrobarometer Round 7	2014-2017	2,180
Croatia	Friedrich-Ebert-Stiftung Youth Studies in Southeast Europe; World Values Survey Wave 3; International Social Survey Programme Religion III; International Social Survey Programme Religion IV; Life in Transition Survey Round 2; Life in Transition Survey Round 3; Pew Religious Belief and National Belonging in Central and Eastern Europe; European Values Study Wave 5; Second European Union Minorities and Discrimination Survey; Eurobarometer Round 83.4; Eurobarometer Round 91.4	1996-2019	36,247
Cyprus	World Values Survey Wave 5; World Values Survey Wave 6; World Values Survey Wave 7; International Social Survey Programme Religion III; Life in Transition Survey Round 3; Second European Union Minorities and Discrimination Survey; Candidate Eurobarometer 2002; Eurobarometer Round 69.1; Eurobarometer Round 83.4; Eurobarometer Round 91.4	2002-2019	13,554

Table S13: (*continued*) List of surveys and coverage by country.

Country	Source(s)	Range of years covered	N
Czechia	Pew Global Attitudes Project Fall 2009; Pew Global Attitudes Project Spring 2019; World Values Survey Wave 2; International Social Survey Programme Religion III; International Social Survey Programme Religion IV; Life in Transition Survey Round 2; Life in Transition Survey Round 3; Pew Religious Belief and National Belonging in Central and Eastern Europe; European Values Study Wave 5; Second European Union Minorities and Discrimination Survey; Candidate Eurobarometer 2002; Eurobarometer Round 69.1; Eurobarometer Round 83.4; Eurobarometer Round 91.4	1990-2019	17,007
Czechoslovakia	Pulse of Europe	1991	387
Denmark	International Social Survey Programme Religion III; International Social Survey Programme Religion IV; Perception and Acceptance of Religious Diversity among the European Population; European Values Study Wave 5; Second European Union Minorities and Discrimination Survey; Eurobarometer Round 30; Eurobarometer Round 37.0; Eurobarometer Round 39.0; Eurobarometer Round 42; Eurobarometer Round 47.2; Eurobarometer Round 69.1; Eurobarometer Round 83.4; Eurobarometer Round 91.4	1988-2019	40,498
Djibouti	Pew: Islam and Christianity in Sub-Saharan Africa	2009	2,506
Dominican Republic	International Social Survey Programme Religion III	2008	8,691
Ecuador	World Values Survey Wave 6; World Values Survey Wave 7; LatinoBarometero 1998	1998-2018	3,991
Egypt	Pew Global Attitudes Project Spring 2008; Pew Global Attitudes Project Spring 2009; Pew Global Attitudes Project Spring 2010; Pew Global Attitudes Project Spring 2011; Pew Global Attitudes Project Spring 2006; World Values Survey Wave 4; World Values Survey Wave 7; Comparative Cross-National Study of Religious Fundamentalism, Developmental Idealism, Values, and Morality in the Middle East and North Africa; Comparative Panel Survey on the Dynamics of Change: Belief Formation and Political Engagement in Egypt, Tunisia, and Turkey Wave 2; The Youth, Emotional Energy, and Political Violence: The Cases of Egypt and Saudi Arabia; Pew: The World's Muslims—Religion, Politics and Society; Arab Barometer Wave 2; Arab Barometer Wave 4; Arab Barometer Wave 5; Arab Opinion Index Wave 7	2000-2020	38,944
El Salvador	World Values Survey Wave 3; LatinoBarometero 1998	1998-1999	2,929
Estonia	World Values Survey Wave 3; World Values Survey Wave 6; Life in Transition Survey Round 2; Life in Transition Survey Round 3; Pew Religious Belief and National Belonging in Central and Eastern Europe; Second European Union Minorities and Discrimination Survey; Candidate Eurobarometer 2002; Eurobarometer Round 69.1; Eurobarometer Round 83.4; Eurobarometer Round 91.4; CROss-National Online Survey Wave 1	1996-2019	20,008
Eswatini	Afrobarometer Round 6; Afrobarometer Round 7	2015-2018	2,275
Ethiopia	World Values Survey Wave 5; World Values Survey Wave 7; Pew: Islam and Christianity in Sub-Saharan Africa	2007-2020	5,513
Finland	World Values Survey Wave 3; World Values Survey Wave 5; International Social Survey Programme Religion III; International Social Survey Programme Religion IV; European Values Study Wave 5; Second European Union Minorities and Discrimination Survey; Eurobarometer Round 47.2; Eurobarometer Round 69.1; Eurobarometer Round 83.4; Eurobarometer Round 91.4	1996-2019	23,098

Table S13: (*continued*) List of surveys and coverage by country.

Country	Source(s)	Range of years covered	N
France	Pew Global Attitudes Project Spring 2004; Pew Global Attitudes Project Spring 2005; Pew Global Attitudes Project Spring 2008; Pew Global Attitudes Project Spring 2009; Pew Global Attitudes Project Fall 2009; Pew Global Attitudes Project Spring 2010; Pew Global Attitudes Project Spring 2011; Pew Global Attitudes Project Spring 2014; Pew Global Attitudes Project Spring 2015; Pew Global Attitudes Project Spring 2016; Pew Global Attitudes Project Spring 2019; Pew Global Attitudes Project Spring 2006; Pulse of Europe; World Values Survey Wave 5; International Social Survey Programme Religion III; International Social Survey Programme Religion IV; A Cross-National Survey of Muslim Attitudes Wave 1; A Cross-National Survey of Muslim Attitudes Wave 2; Perception and Acceptance of Religious Diversity among the European Population; Life in Transition Survey Round 2; Second European Union Minorities and Discrimination Survey; Eurobarometer Round 30; Eurobarometer Round 37.0; Eurobarometer Round 39.0; Eurobarometer Round 42; Eurobarometer Round 47.2; Eurobarometer Round 69.1; Eurobarometer Round 83.4; Eurobarometer Round 91.4	1988-2019	61,215
Gabon	Afrobarometer Round 6; Afrobarometer Round 7	2015-2017	2,110
Gambia	Afrobarometer Round 7	2018	1,187
Georgia	Caucasus Barometer 2019; Caucasus Barometer 2017; Caucasus Barometer 2015; Caucasus Barometer 2013; Caucasus Barometer 2012; Caucasus Barometer 2011; Caucasus Barometer 2010; Caucasus Barometer 2009; World Values Survey Wave 3; World Values Survey Wave 5; World Values Survey Wave 6; International Social Survey Programme Religion IV; Life in Transition Survey Round 2; Life in Transition Survey Round 3; Pew Religious Belief and National Belonging in Central and Eastern Europe	1996-2020	67,757
Germany	Pew Global Attitudes Project Spring 2004; Pew Global Attitudes Project Spring 2005; Pew Global Attitudes Project Spring 2008; Pew Global Attitudes Project Spring 2009; Pew Global Attitudes Project Fall 2009; Pew Global Attitudes Project Spring 2010; Pew Global Attitudes Project Spring 2011; Pew Global Attitudes Project Spring 2014; Pew Global Attitudes Project Spring 2015; Pew Global Attitudes Project Spring 2016; Pew Global Attitudes Project Spring 2019; Pew Global Attitudes Project Spring 2006; Pulse of Europe; World Values Survey Wave 3; World Values Survey Wave 5; World Values Survey Wave 6; World Values Survey Wave 7; International Social Survey Programme Religion III; International Social Survey Programme Religion IV; A Cross-National Survey of Muslim Attitudes Wave 1; A Cross-National Survey of Muslim Attitudes Wave 2; Perception and Acceptance of Religious Diversity among the European Population; Life in Transition Survey Round 2; Life in Transition Survey Round 3; European Values Study Wave 5; Second European Union Minorities and Discrimination Survey; Eurobarometer Round 30; Eurobarometer Round 37.0; Eurobarometer Round 39.0; Eurobarometer Round 42; Eurobarometer Round 47.2; Eurobarometer Round 69.1; Eurobarometer Round 83.4; Eurobarometer Round 91.4	1988-2019	76,285
Ghana	World Values Survey Wave 5; World Values Survey Wave 6; International Social Survey Programme Religion IV; Pew: Islam and Christianity in Sub-Saharan Africa; Afrobarometer Round 6; Afrobarometer Round 7	2007-2018	18,851
Greece	Pew Global Attitudes Project Spring 2014; Pew Global Attitudes Project Spring 2016; Pew Global Attitudes Project Spring 2019; World Values Survey Wave 7; Life in Transition Survey Round 3; Pew Religious Belief and National Belonging in Central and Eastern Europe; Second European Union Minorities and Discrimination Survey; Eurobarometer Round 30; Eurobarometer Round 37.0; Eurobarometer Round 39.0; Eurobarometer Round 42; Eurobarometer Round 47.2; Eurobarometer Round 69.1; Eurobarometer Round 83.4; Eurobarometer Round 91.4	1988-2019	41,235
Guatemala	World Values Survey Wave 5; World Values Survey Wave 7; LatinoBarometero 1998	1998-2020	3,841
Guinea	Afrobarometer Round 6; Afrobarometer Round 7	2015-2017	2,354
Guinea-Bissau	Pew: Islam and Christianity in Sub-Saharan Africa	2009	1,782
Haiti	World Values Survey Wave 6	2016	1,700
Honduras	LatinoBarometero 1998	1998	1,768

Table S13: (*continued*) List of surveys and coverage by country.

Country	Source(s)	Range of years covered	N
Hungary	Pew Global Attitudes Project Fall 2009; Pew Global Attitudes Project Spring 2016; Pew Global Attitudes Project Spring 2019; Pulse of Europe; World Values Survey Wave 5; International Social Survey Programme Religion III; International Social Survey Programme Religion IV; Life in Transition Survey Round 2; Life in Transition Survey Round 3; Pew Religious Belief and National Belonging in Central and Eastern Europe; Second European Union Minorities and Discrimination Survey; Candidate Eurobarometer 2002; Eurobarometer Round 69.1; Eurobarometer Round 83.4; Eurobarometer Round 91.4	1991-2019	37,168
Iceland	International Social Survey Programme Religion IV; European Values Study Wave 5	2017-2019	5,269
India	Pew Global Attitudes Project Spring 2005; Pew Global Attitudes Project Spring 2008; Pew Global Attitudes Project Spring 2009; Pew Global Attitudes Project Spring 2010; Pew Global Attitudes Project Spring 2011; Pew Global Attitudes Project Spring 2006; World Values Survey Wave 2; World Values Survey Wave 3; World Values Survey Wave 4; World Values Survey Wave 5; World Values Survey Wave 6	1990-2012	47,128
Indonesia	Pew Global Attitudes Project Spring 2005; Pew Global Attitudes Project Spring 2008; Pew Global Attitudes Project Spring 2009; Pew Global Attitudes Project Spring 2010; Pew Global Attitudes Project Spring 2011; Pew Global Attitudes Project Spring 2006; World Values Survey Wave 4; World Values Survey Wave 5; World Values Survey Wave 7; International Social Survey Programme Religion III; International Social Survey Programme Religion IV; A Cross-National Survey of Muslim Attitudes Wave 2; Pew: The World's Muslims—Religion, Politics and Society	2001-2018	36,701
Iran	World Values Survey Wave 4; World Values Survey Wave 5; World Values Survey Wave 7; Pew: The World's Muslims—Religion, Politics and Society	2000-2020	14,531
Iraq	World Values Survey Wave 4; World Values Survey Wave 5; World Values Survey Wave 6; World Values Survey Wave 7; Comparative Cross-National Study of Religious Fundamentalism, Developmental Idealism, Values, and Morality in the Middle East and North Africa; Pew: The World's Muslims—Religion, Politics and Society; Arab Barometer Wave 2; Arab Barometer Wave 5; Arab Barometer Wave 6; Arab Opinion Index Wave 7	2004-2021	44,236
Ireland	International Social Survey Programme Religion III; Second European Union Minorities and Discrimination Survey; Eurobarometer Round 30; Eurobarometer Round 37.0; Eurobarometer Round 39.0; Eurobarometer Round 42; Eurobarometer Round 47.2; Eurobarometer Round 69.1; Eurobarometer Round 83.4; Eurobarometer Round 91.4	1988-2019	26,594
Israel	Pew Global Attitudes Project Spring 2009; Pew Global Attitudes Project Spring 2011; International Social Survey Programme Religion III; International Social Survey Programme Religion IV	2009-2018	14,369
Italy	Pew Global Attitudes Project Fall 2009; Pew Global Attitudes Project Spring 2014; Pew Global Attitudes Project Spring 2015; Pew Global Attitudes Project Spring 2016; Pew Global Attitudes Project Spring 2019; World Values Survey Wave 5; International Social Survey Programme Religion III; International Social Survey Programme Religion IV; Life in Transition Survey Round 2; Life in Transition Survey Round 3; Second European Union Minorities and Discrimination Survey; Eurobarometer Round 30; Eurobarometer Round 37.0; Eurobarometer Round 39.0; Eurobarometer Round 42; Eurobarometer Round 47.2; Eurobarometer Round 69.1; Eurobarometer Round 83.4; Eurobarometer Round 91.4	1988-2019	50,363
Japan	Pew Global Attitudes Project Spring 2008; Pew Global Attitudes Project Spring 2009; Pew Global Attitudes Project Spring 2010; Pew Global Attitudes Project Spring 2011; Pew Global Attitudes Project Spring 2006; World Values Survey Wave 2; World Values Survey Wave 6; World Values Survey Wave 7; International Social Survey Programme Religion III; International Social Survey Programme Religion IV	1990-2019	7,803

Table S13: (*continued*) List of surveys and coverage by country.

Country	Source(s)	Range of years covered	N
Jordan	Pew Global Attitudes Project Spring 2005; Pew Global Attitudes Project Spring 2008; Pew Global Attitudes Project Spring 2009; Pew Global Attitudes Project Spring 2010; Pew Global Attitudes Project Spring 2011; Pew Global Attitudes Project Spring 2006; World Values Survey Wave 4; World Values Survey Wave 5; World Values Survey Wave 6; World Values Survey Wave 7; International Social Survey Programme Religion IV; Comparative Cross-National Study of Religious Fundamentalism, Developmental Idealism, Values, and Morality in the Middle East and North Africa; Pew: The World's Muslims—Religion, Politics and Society; Arab Barometer Wave 1; Arab Barometer Wave 2; Arab Barometer Wave 4; Arab Barometer Wave 5; Arab Barometer Wave 6; Arab Opinion Index Wave 7	2001-2021	52,621
Kazakhstan	World Values Survey Wave 6; World Values Survey Wave 7; Life in Transition Survey Round 2; Life in Transition Survey Round 3; Pew Religious Belief and National Belonging in Central and Eastern Europe; Pew: The World's Muslims—Religion, Politics and Society; Friedrich-Ebert-Stiftung Youth Studies in East Europe and Central Asia	2010-2018	15,903
Kenya	Pew Global Attitudes Project Spring 2009; Pew Global Attitudes Project Spring 2010; Pew Global Attitudes Project Spring 2011; World Values Survey Wave 7; International Social Survey Programme Religion III; International Social Survey Programme Religion IV; Pew: Islam and Christianity in Sub-Saharan Africa; Afrobarometer Round 6; Afrobarometer Round 7	2008-2021	23,461
Kosovo	Friedrich-Ebert-Stiftung Youth Studies in Southeast Europe; Life in Transition Survey Round 2; Life in Transition Survey Round 3; Pew: The World's Muslims—Religion, Politics and Society; Friedrich-Ebert-Stiftung Youth Studies in East Europe and Central Asia	2000-2017	9,613
Kuwait	Arab Opinion Index Wave 7	2019	1,452
Kyrgyzstan	World Values Survey Wave 4; World Values Survey Wave 6; World Values Survey Wave 7; Life in Transition Survey Round 2; Life in Transition Survey Round 3; Pew: The World's Muslims—Religion, Politics and Society; Friedrich-Ebert-Stiftung Youth Studies in East Europe and Central Asia	2000-2020	11,602
Latvia	World Values Survey Wave 3; International Social Survey Programme Religion III; Life in Transition Survey Round 2; Life in Transition Survey Round 3; Pew Religious Belief and National Belonging in Central and Eastern Europe; Second European Union Minorities and Discrimination Survey; Candidate Eurobarometer 2002; Eurobarometer Round 69.1; Eurobarometer Round 83.4; Eurobarometer Round 91.4	1996-2019	29,756
Lebanon	Pew Global Attitudes Project Spring 2005; Pew Global Attitudes Project Spring 2008; Pew Global Attitudes Project Spring 2009; Pew Global Attitudes Project Spring 2010; Pew Global Attitudes Project Spring 2011; World Values Survey Wave 6; World Values Survey Wave 7; Comparative Cross-National Study of Religious Fundamentalism, Developmental Idealism, Values, and Morality in the Middle East and North Africa; Pew: The World's Muslims—Religion, Politics and Society; Arab Barometer Wave 1; Arab Barometer Wave 2; Arab Barometer Wave 4; Arab Barometer Wave 5; Arab Barometer Wave 6; Arab Opinion Index Wave 7	2005-2021	46,119
Lesotho	Afrobarometer Round 6; Afrobarometer Round 7	2014-2017	2,315
Liberia	Pew: Islam and Christianity in Sub-Saharan Africa; Afrobarometer Round 6; Afrobarometer Round 7	2009-2018	5,260
Libya	World Values Survey Wave 6; World Values Survey Wave 7; Arab Barometer Wave 5; Arab Barometer Wave 6	2014-2022	9,032
Lithuania	Pew Global Attitudes Project Fall 2009; Pew Global Attitudes Project Spring 2011; Pew Global Attitudes Project Spring 2019; Pulse of Europe; International Social Survey Programme Religion IV; Life in Transition Survey Round 2; Life in Transition Survey Round 3; Pew Religious Belief and National Belonging in Central and Eastern Europe; Second European Union Minorities and Discrimination Survey; Candidate Eurobarometer 2002; Eurobarometer Round 69.1; Eurobarometer Round 83.4; Eurobarometer Round 91.4	1991-2019	41,585
Luxembourg	Second European Union Minorities and Discrimination Survey; Eurobarometer Round 30; Eurobarometer Round 37.0; Eurobarometer Round 39.0; Eurobarometer Round 42; Eurobarometer Round 47.2; Eurobarometer Round 69.1; Eurobarometer Round 83.4; Eurobarometer Round 91.4	1988-2019	7,215

Table S13: (*continued*) List of surveys and coverage by country.

Country	Source(s)	Range of years covered	N
Madagascar	Afrobarometer Round 6; Afrobarometer Round 7	2014-2018	2,190
Malawi	International Social Survey Programme Religion IV; Afrobarometer Round 6; Afrobarometer Round 7	2014-2018	12,344
Malaysia	World Values Survey Wave 5; World Values Survey Wave 6; World Values Survey Wave 7; International Social Survey Programme Religion IV; A Cross-National Survey of Muslim Attitudes Wave 1; A Cross-National Survey of Muslim Attitudes Wave 2; Pew: The World's Muslims—Religion, Politics and Society	2006-2018	19,095
Mali	World Values Survey Wave 5; Pew: Islam and Christianity in Sub-Saharan Africa; Afrobarometer Round 6; Afrobarometer Round 7	2007-2017	5,467
Malta	Second European Union Minorities and Discrimination Survey; Candidate Eurobarometer 2002; Eurobarometer Round 69.1; Eurobarometer Round 83.4; Eurobarometer Round 91.4	2002-2019	6,798
Mauritania	Arab Opinion Index Wave 7	2020	1,726
Mauritius	Afrobarometer Round 6; Afrobarometer Round 7	2014-2017	2,319
Mexico	Pew Global Attitudes Project Spring 2008; Pew Global Attitudes Project Spring 2009; Pew Global Attitudes Project Spring 2010; Pew Global Attitudes Project Spring 2011; World Values Survey Wave 2; World Values Survey Wave 3; World Values Survey Wave 4; World Values Survey Wave 5; World Values Survey Wave 6; World Values Survey Wave 7; International Social Survey Programme Religion III; LatinoBarometero 1998	1990-2018	29,461
Moldova	World Values Survey Wave 3; World Values Survey Wave 4; World Values Survey Wave 5; Life in Transition Survey Round 2; Life in Transition Survey Round 3; Pew Religious Belief and National Belonging in Central and Eastern Europe	1996-2016	24,040
Mongolia	World Values Survey Wave 7; International Social Survey Programme Religion IV; Life in Transition Survey Round 2; Life in Transition Survey Round 3	2010-2021	9,056
Montenegro	Friedrich-Ebert-Stiftung Youth Studies in Southeast Europe; World Values Survey Wave 4; Life in Transition Survey Round 2; Life in Transition Survey Round 3	2000-2018	6,142
Morocco	Pew Global Attitudes Project Spring 2005; World Values Survey Wave 5; World Values Survey Wave 6; World Values Survey Wave 7; Pew: The World's Muslims—Religion, Politics and Society; Afrobarometer Round 6; Afrobarometer Round 7; Arab Barometer Wave 4; Arab Barometer Wave 5; Arab Barometer Wave 6; Arab Opinion Index Wave 7	2005-2021	20,909
Mozambique	Pew: Islam and Christianity in Sub-Saharan Africa; Afrobarometer Round 6; Afrobarometer Round 7	2009-2018	6,655
Myanmar	World Values Survey Wave 7	2020	1,111
Namibia	Afrobarometer Round 6; Afrobarometer Round 7	2014-2017	2,322
Nepal	International Social Survey Programme Religion IV	2018-2019	7,295
Netherlands	Pew Global Attitudes Project Spring 2005; Pew Global Attitudes Project Spring 2016; Pew Global Attitudes Project Spring 2019; World Values Survey Wave 5; World Values Survey Wave 6; International Social Survey Programme Religion III; Perception and Acceptance of Religious Diversity among the European Population; European Values Study Wave 5; Second European Union Minorities and Discrimination Survey; Eurobarometer Round 30; Eurobarometer Round 37.0; Eurobarometer Round 39.0; Eurobarometer Round 42; Eurobarometer Round 47.2; Eurobarometer Round 69.1; Eurobarometer Round 83.4; Eurobarometer Round 91.4	1988-2019	24,750
New Zealand	World Values Survey Wave 6; World Values Survey Wave 7; International Social Survey Programme Religion III; International Social Survey Programme Religion IV	2008-2020	9,345

Table S13: (*continued*) List of surveys and coverage by country.

Country	Source(s)	Range of years covered	N
Nicaragua	World Values Survey Wave 7; LatinoBarometero 1998	1998-2020	2,807
Niger	A Cross-National Survey of Muslim Attitudes Wave 1; A Cross-National Survey of Muslim Attitudes Wave 2; Pew: The World's Muslims—Religion, Politics and Society; Afrobarometer Round 6; Afrobarometer Round 7	2011-2018	5,644
Nigeria	Pew Global Attitudes Project Spring 2008; Pew Global Attitudes Project Spring 2009; Pew Global Attitudes Project Spring 2010; Pew Global Attitudes Project Spring 2006; World Values Survey Wave 2; World Values Survey Wave 3; World Values Survey Wave 4; World Values Survey Wave 6; World Values Survey Wave 7; International Social Survey Programme Religion IV; A Cross-National Survey of Muslim Attitudes Wave 1; A Cross-National Survey of Muslim Attitudes Wave 2; Pew: Islam and Christianity in Sub-Saharan Africa; Afrobarometer Round 6; Afrobarometer Round 7	1990-2018	36,825
North Macedonia	Friedrich-Ebert-Stiftung Youth Studies in Southeast Europe; World Values Survey Wave 4; Life in Transition Survey Round 2; Life in Transition Survey Round 3	2000-2018	7,506
Norway	World Values Survey Wave 3; World Values Survey Wave 5; International Social Survey Programme Religion III; International Social Survey Programme Religion IV; Eurobarometer Round 37.0; Eurobarometer Round 39.0; Eurobarometer Round 42	1992-2019	8,540
Pakistan	Pew Global Attitudes Project Spring 2004; Pew Global Attitudes Project Spring 2005; Pew Global Attitudes Project Spring 2008; Pew Global Attitudes Project Spring 2009; Pew Global Attitudes Project Spring 2010; Pew Global Attitudes Project Spring 2011; Pew Global Attitudes Project Spring 2006; World Values Survey Wave 4; World Values Survey Wave 6; World Values Survey Wave 7; Comparative Cross-National Study of Religious Fundamentalism, Developmental Idealism, Values, and Morality in the Middle East and North Africa; Pew: The World's Muslims—Religion, Politics and Society	2001-2018	43,177
Palestine	Pew Global Attitudes Project Spring 2009; Pew Global Attitudes Project Spring 2011; World Values Survey Wave 6; Pew: The World's Muslims—Religion, Politics and Society; Arab Barometer Wave 2; Arab Barometer Wave 4; Arab Barometer Wave 5; Arab Opinion Index Wave 7	2009-2019	22,598
Panama	LatinoBarometero 1998	1998	1,917
Paraguay	LatinoBarometero 1998	1998	1,173
Peru	World Values Survey Wave 4; World Values Survey Wave 5; World Values Survey Wave 6; World Values Survey Wave 7; LatinoBarometero 1998	1998-2018	7,087
Philippines	World Values Survey Wave 3; World Values Survey Wave 4; World Values Survey Wave 6; World Values Survey Wave 7; International Social Survey Programme Religion III; International Social Survey Programme Religion IV	1996-2019	14,794
Poland	Pew Global Attitudes Project Spring 2005; Pew Global Attitudes Project Spring 2008; Pew Global Attitudes Project Spring 2009; Pew Global Attitudes Project Fall 2009; Pew Global Attitudes Project Spring 2010; Pew Global Attitudes Project Spring 2011; Pew Global Attitudes Project Spring 2014; Pew Global Attitudes Project Spring 2015; Pew Global Attitudes Project Spring 2016; Pew Global Attitudes Project Spring 2019; Pulse of Europe; World Values Survey Wave 5; World Values Survey Wave 6; International Social Survey Programme Religion III; Life in Transition Survey Round 2; Life in Transition Survey Round 3; Pew Religious Belief and National Belonging in Central and Eastern Europe; European Values Study Wave 5; Second European Union Minorities and Discrimination Survey; Candidate Eurobarometer 2002; Eurobarometer Round 69.1; Eurobarometer Round 83.4; Eurobarometer Round 91.4	1991-2019	58,564
Portugal	International Social Survey Programme Religion III; Perception and Acceptance of Religious Diversity among the European Population; Second European Union Minorities and Discrimination Survey; Eurobarometer Round 30; Eurobarometer Round 37.0; Eurobarometer Round 39.0; Eurobarometer Round 42; Eurobarometer Round 47.2; Eurobarometer Round 69.1; Eurobarometer Round 83.4; Eurobarometer Round 91.4	1988-2019	21,432

Table S13: (*continued*) List of surveys and coverage by country.

Country	Source(s)	Range of years covered	N
Qatar	World Values Survey Wave 6; Arab Opinion Index Wave 7	2010-2020	1,357
Romania	Friedrich-Ebert-Stiftung Youth Studies in Southeast Europe; World Values Survey Wave 3; World Values Survey Wave 5; World Values Survey Wave 6; World Values Survey Wave 7; Life in Transition Survey Round 2; Life in Transition Survey Round 3; Pew Religious Belief and National Belonging in Central and Eastern Europe; Second European Union Minorities and Discrimination Survey; Friedrich-Ebert-Stiftung Youth Studies in East Europe and Central Asia; Candidate Eurobarometer 2002; Eurobarometer Round 69.1; Eurobarometer Round 83.4; Eurobarometer Round 91.4	1998-2019	39,621
Russia	Pew Global Attitudes Project Spring 2004; Pew Global Attitudes Project Spring 2005; Pew Global Attitudes Project Spring 2008; Pew Global Attitudes Project Spring 2009; Pew Global Attitudes Project Fall 2009; Pew Global Attitudes Project Spring 2010; Pew Global Attitudes Project Spring 2011; Pew Global Attitudes Project Spring 2019; Pew Global Attitudes Project Spring 2006; Pulse of Europe; World Values Survey Wave 2; World Values Survey Wave 3; World Values Survey Wave 5; World Values Survey Wave 6; World Values Survey Wave 7; International Social Survey Programme Religion III; International Social Survey Programme Religion IV; Life in Transition Survey Round 2; Life in Transition Survey Round 3; Pew Religious Belief and National Belonging in Central and Eastern Europe; European Values Study Wave 5; Pew: The World's Muslims—Religion, Politics and Society	1982-2019	50,911
Rwanda	World Values Survey Wave 5; World Values Survey Wave 6; Pew: Islam and Christianity in Sub-Saharan Africa	2007-2012	4,552
São Tomé & Príncipe	Afrobarometer Round 6; Afrobarometer Round 7	2015-2018	2,142
Saudi Arabia	World Values Survey Wave 4; The Youth, Emotional Energy, and Political Violence: The Cases of Egypt and Saudi Arabia; Arab Barometer Wave 2; Arab Opinion Index Wave 7	2003-2020	5,959
Senegal	A Cross-National Survey of Muslim Attitudes Wave 1; A Cross-National Survey of Muslim Attitudes Wave 2; Pew: Islam and Christianity in Sub-Saharan Africa; Afrobarometer Round 6; Afrobarometer Round 7	2009-2017	5,754
Serbia	Friedrich-Ebert-Stiftung Youth Studies in Southeast Europe; World Values Survey Wave 4; World Values Survey Wave 5; World Values Survey Wave 7; Life in Transition Survey Round 2; Life in Transition Survey Round 3; Pew Religious Belief and National Belonging in Central and Eastern Europe; Friedrich-Ebert-Stiftung Youth Studies in East Europe and Central Asia	2000-2018	23,356
Sierra Leone	Afrobarometer Round 6; Afrobarometer Round 7	2015-2018	2,351
Singapore	World Values Survey Wave 6; World Values Survey Wave 7; International Social Survey Programme Religion IV	2012-2019	15,202
Slovakia	Pew Global Attitudes Project Fall 2009; Pew Global Attitudes Project Spring 2019; World Values Survey Wave 2; International Social Survey Programme Religion III; International Social Survey Programme Religion IV; Life in Transition Survey Round 2; Life in Transition Survey Round 3; European Values Study Wave 5; Second European Union Minorities and Discrimination Survey; Candidate Eurobarometer 2002; Eurobarometer Round 69.1; Eurobarometer Round 83.4; Eurobarometer Round 91.4	1990-2019	31,611
Slovenia	Friedrich-Ebert-Stiftung Youth Studies in Southeast Europe; World Values Survey Wave 3; World Values Survey Wave 5; World Values Survey Wave 6; International Social Survey Programme Religion III; International Social Survey Programme Religion IV; Life in Transition Survey Round 2; Life in Transition Survey Round 3; European Values Study Wave 5; Second European Union Minorities and Discrimination Survey; Friedrich-Ebert-Stiftung Youth Studies in East Europe and Central Asia; Candidate Eurobarometer 2002; Eurobarometer Round 69.1; Eurobarometer Round 83.4; Eurobarometer Round 91.4; CROss-National Online Survey Wave 1	1995-2019	22,360
South Africa	Pew Global Attitudes Project Spring 2008; World Values Survey Wave 4; World Values Survey Wave 5; World Values Survey Wave 6; International Social Survey Programme Religion III; International Social Survey Programme Religion IV; Pew: Islam and Christianity in Sub-Saharan Africa; Afrobarometer Round 6; Afrobarometer Round 7	2001-2019	48,260

Table S13: (*continued*) List of surveys and coverage by country.

Country	Source(s)	Range of years covered	N
South Korea	Pew Global Attitudes Project Spring 2008; Pew Global Attitudes Project Spring 2009; Pew Global Attitudes Project Spring 2010; World Values Survey Wave 1; World Values Survey Wave 2; World Values Survey Wave 4; World Values Survey Wave 5; World Values Survey Wave 6; World Values Survey Wave 7; International Social Survey Programme Religion III; International Social Survey Programme Religion IV	1982-2018	14,468
Spain	Pew Global Attitudes Project Spring 2005; Pew Global Attitudes Project Spring 2008; Pew Global Attitudes Project Spring 2009; Pew Global Attitudes Project Fall 2009; Pew Global Attitudes Project Spring 2010; Pew Global Attitudes Project Spring 2011; Pew Global Attitudes Project Spring 2014; Pew Global Attitudes Project Spring 2015; Pew Global Attitudes Project Spring 2016; Pew Global Attitudes Project Spring 2019; Pew Global Attitudes Project Spring 2006; World Values Survey Wave 2; World Values Survey Wave 3; World Values Survey Wave 4; World Values Survey Wave 5; World Values Survey Wave 6; International Social Survey Programme Religion III; International Social Survey Programme Religion IV; LatinoBarometero 1998; European Values Study Wave 5; Second European Union Minorities and Discrimination Survey; Eurobarometer Round 30; Eurobarometer Round 37.0; Eurobarometer Round 39.0; Eurobarometer Round 42; Eurobarometer Round 47.2; Eurobarometer Round 69.1; Eurobarometer Round 83.4; Eurobarometer Round 91.4	1988-2019	60,835
Sri Lanka	International Social Survey Programme Religion III; International Social Survey Programme Religion IV	2008-2018	6,648
Sudan	Afrobarometer Round 7; Arab Barometer Wave 2; Arab Barometer Wave 5; Arab Opinion Index Wave 7	2010-2019	9,021
Suriname	International Social Survey Programme Religion IV	2019-2020	3,770
Sweden	Pew Global Attitudes Project Spring 2016; Pew Global Attitudes Project Spring 2019; World Values Survey Wave 2; World Values Survey Wave 3; World Values Survey Wave 4; World Values Survey Wave 5; World Values Survey Wave 6; International Social Survey Programme Religion III; International Social Survey Programme Religion IV; Life in Transition Survey Round 2; European Values Study Wave 5; Second European Union Minorities and Discrimination Survey; Eurobarometer Round 47.2; Eurobarometer Round 69.1; Eurobarometer Round 83.4; Eurobarometer Round 91.4	1990-2019	26,653
Switzerland	World Values Survey Wave 2; World Values Survey Wave 3; World Values Survey Wave 5; International Social Survey Programme Religion III; International Social Survey Programme Religion IV; European Values Study Wave 5	1989-2018	22,028
Taiwan	World Values Survey Wave 5; World Values Survey Wave 6; World Values Survey Wave 7; International Social Survey Programme Religion III; International Social Survey Programme Religion IV	2006-2019	12,042
Tajikistan	World Values Survey Wave 7; Life in Transition Survey Round 2; Life in Transition Survey Round 3; Pew: The World's Muslims—Religion, Politics and Society; Friedrich-Ebert-Stiftung Youth Studies in East Europe and Central Asia	2010-2020	9,384
Tanzania	Pew Global Attitudes Project Spring 2008; World Values Survey Wave 4; International Social Survey Programme Religion III; Pew: Islam and Christianity in Sub-Saharan Africa; Afrobarometer Round 6; Afrobarometer Round 7	2001-2017	13,636
Thailand	World Values Survey Wave 5; World Values Survey Wave 6; World Values Survey Wave 7; International Social Survey Programme Religion IV; Pew: The World's Muslims—Religion, Politics and Society	2007-2019	16,699
Togo	Afrobarometer Round 6; Afrobarometer Round 7	2014-2017	2,264
Trinidad & Tobago	World Values Survey Wave 5; World Values Survey Wave 6	2006-2011	1,852

Table S13: (*continued*) List of surveys and coverage by country.

Country	Source(s)	Range of years covered	N
Tunisia	World Values Survey Wave 6; World Values Survey Wave 7; Comparative Cross-National Study of Religious Fundamentalism, Developmental Idealism, Values, and Morality in the Middle East and North Africa; Comparative Panel Survey on the Dynamics of Change: Belief Formation and Political Engagement in Egypt, Tunisia, and Turkey Wave 1; Pew: The World's Muslims—Religion, Politics and Society; Afrobarometer Round 6; Afrobarometer Round 7; Arab Barometer Wave 2; Arab Barometer Wave 4; Arab Barometer Wave 5; Arab Barometer Wave 6; Arab Opinion Index Wave 7	2011-2021	37,036
Turkey	Pew Global Attitudes Project Spring 2004; Pew Global Attitudes Project Spring 2005; Pew Global Attitudes Project Spring 2008; Pew Global Attitudes Project Spring 2009; Pew Global Attitudes Project Spring 2010; Pew Global Attitudes Project Spring 2011; Pew Global Attitudes Project Spring 2006; World Values Survey Wave 2; World Values Survey Wave 3; World Values Survey Wave 4; World Values Survey Wave 5; World Values Survey Wave 6; World Values Survey Wave 7; International Social Survey Programme Religion III; International Social Survey Programme Religion IV; Comparative Cross-National Study of Religious Fundamentalism, Developmental Idealism, Values, and Morality in the Middle East and North Africa; Comparative Panel Survey on the Dynamics of Change: Belief Formation and Political Engagement in Egypt, Tunisia, and Turkey Wave 1; Comparative Panel Survey on the Dynamics of Change: Belief Formation and Political Engagement in Egypt, Tunisia, and Turkey Wave 2; Life in Transition Survey Round 2; Life in Transition Survey Round 3; Pew: The World's Muslims—Religion, Politics and Society; Candidate Eurobarometer 2002	1990-2019	65,841
Uganda	World Values Survey Wave 4; Pew: Islam and Christianity in Sub-Saharan Africa; Afrobarometer Round 6; Afrobarometer Round 7	2001-2017	7,282
UK	Pew Global Attitudes Project Spring 2004; Pew Global Attitudes Project Spring 2005; Pew Global Attitudes Project Spring 2008; Pew Global Attitudes Project Spring 2009; Pew Global Attitudes Project Fall 2009; Pew Global Attitudes Project Spring 2010; Pew Global Attitudes Project Spring 2011; Pew Global Attitudes Project Spring 2014; Pew Global Attitudes Project Spring 2015; Pew Global Attitudes Project Spring 2016; Pew Global Attitudes Project Spring 2019; Pew Global Attitudes Project Spring 2006; World Values Survey Wave 5; International Social Survey Programme Religion III; International Social Survey Programme Religion IV; A Cross-National Survey of Muslim Attitudes Wave 1; A Cross-National Survey of Muslim Attitudes Wave 2; Life in Transition Survey Round 2; Second European Union Minorities and Discrimination Survey; Eurobarometer Round 30; Eurobarometer Round 37.0; Eurobarometer Round 39.0; Eurobarometer Round 42; Eurobarometer Round 47.2; Eurobarometer Round 69.1; Eurobarometer Round 83.4; Eurobarometer Round 91.4; CROss-National Online Survey Wave 1	1988-2019	49,037
Ukraine	Pew Global Attitudes Project Fall 2009; Pew Global Attitudes Project Spring 2011; Pew Global Attitudes Project Spring 2014; Pew Global Attitudes Project Spring 2019; Pulse of Europe; World Values Survey Wave 3; World Values Survey Wave 5; World Values Survey Wave 6; World Values Survey Wave 7; International Social Survey Programme Religion III; Life in Transition Survey Round 2; Life in Transition Survey Round 3; Pew Religious Belief and National Belonging in Central and Eastern Europe; Friedrich-Ebert-Stiftung Youth Studies in East Europe and Central Asia	1991-2020	36,629
Uruguay	World Values Survey Wave 3; World Values Survey Wave 5; World Values Survey Wave 6; International Social Survey Programme Religion III; LatinoBarometero 1998	1996-2011	6,892
US	Pew Global Attitudes Project Spring 2004; Pew Global Attitudes Project Spring 2005; Pew Global Attitudes Project Spring 2008; Pew Global Attitudes Project Spring 2009; Pew Global Attitudes Project Spring 2010; Pew Global Attitudes Project Spring 2011; Pew Global Attitudes Project Spring 2006; World Values Survey Wave 4; World Values Survey Wave 7; World Values Survey Wave 2; World Values Survey Wave 3; World Values Survey Wave 5; World Values Survey Wave 6; International Social Survey Programme Religion III; International Social Survey Programme Religion IV	1990-2019	34,078
Uzbekistan	World Values Survey Wave 6; Life in Transition Survey Round 2; Life in Transition Survey Round 3; Pew: The World's Muslims—Religion, Politics and Society; Friedrich-Ebert-Stiftung Youth Studies in East Europe and Central Asia	2010-2016	9,846
Venezuela	World Values Survey Wave 3; World Values Survey Wave 4; World Values Survey Wave 7; International Social Survey Programme Religion III; LatinoBarometero 1998	1996-2021	6,145

Table S13: (*continued*) List of surveys and coverage by country.

Country	Source(s)	Range of years covered	N
Vietnam	World Values Survey Wave 4; World Values Survey Wave 5; World Values Survey Wave 7; International Social Survey Programme Religion IV	2001-2020	5,339
Yemen	World Values Survey Wave 6; Arab Barometer Wave 1; Arab Barometer Wave 5	2007-2018	6,412
Zambia	World Values Survey Wave 5; Pew: Islam and Christianity in Sub-Saharan Africa; Afrobarometer Round 6; Afrobarometer Round 7	2007-2017	5,565
Zimbabwe	World Values Survey Wave 4; World Values Survey Wave 6; World Values Survey Wave 7; Afrobarometer Round 6; Afrobarometer Round 7	2001-2020	7,439
